# Positive feedback regulation between USP8 and Hippo/YAP axis drives triple-negative breast cancer progression

**DOI:** 10.1038/s41419-025-08356-8

**Published:** 2026-01-21

**Authors:** Xin Li, Penghe Yang, Tianshi Wang, Peng Su, Chenmiao Zhang, Shen Fangyu, Huijie Yang, Jian Zhu, Xiaodong Tan, Ting Zhuang

**Affiliations:** 1 Xinxiang Key Laboratory of Tumor Migration and Invasion Precision Medicine, School of Medical Technology, Xinxiang Medical University, Xinxiang, Henan Province PR China; 2 Department of Surgical Oncology and General Surgery, The First Hospital of China Medical University, Shenyang, Liaoning Province PR China; 3 Henan Key Laboratory of Immunology and Targeted Therapy, School of Medical Technology, Xinxiang Medical University, Xinxiang, Henan Province PR China; 4 Department of General Surgery, The Second Hospital, Cheeloo College of Medicine, Shandong University, Jinan, Shandong Province PR China; 5 Department of Pathology, Qilu Hospital, Cheeloo College of Medicine, Shandong University, Jinan, Shandong Province PR China; 6 Henan Collaborative Innovation Center of Molecular Diagnosis and Laboratory Medicine, School of Medical Technology, Xinxiang Medical University, Xinxiang, Henan Province PR China; 7 Henan International Joint Laboratory of Immunology and Model Animals, School of Medical Technology, Xinxiang Medical University, Xinxiang, Henan Province PR China; 8 Department of General Surgery, Shengjing Hospital of China Medical University, Shenyang, Liaoning Province PR China

**Keywords:** Cancer, Cell biology

## Abstract

The hyper-activation of the Hippo/YAP axis was observed in triple-negative breast cancer (TNBC), which was crucial for tumor progression. The over-activation of YAP in TNBC remains unexplained, despite the continued functionality of the inhibitory phospho-cascade. Recently, studies revealed that the ubiquitin modifications of YAP also play important roles in the Hippo/YAP axis and cancer progression. In order to understand the potential mechanisms of ubiquitination and deubiquitination process in YAP function, we carried out siRNA screening for critical deubiquitinases in TNBC. Via the deubiquitinases (DUB) library, we identified Ubiquitin Specific Peptidase 8 (USP8) as an important effector in YAP function and TNBC progression. Inhibition of USP8 hampered TNBC progression via Hippo signaling. Clinical data revealed that USP8 expression correlated with YAP protein level and poor survival in TNBC patients. Biochemical evaluations revealed that USP8 has the ability to connect with YAP and suppress K48-linked polyubiquitination, thereby enhancing the stability of YAP. Interestingly, YAP directly binds to the USP8 promoter region, enhancing its transcription in TNBC. Our study revealed a forward feedback loop between USP8 and Hippo signaling in TNBC, indicating USP8 as a potential therapeutic drug targets in TNBC.

## Introduction

Breast cancer is the most common women malignancies worldwide, while triple negative breast cancer (TNBC) is the most aggressive subtype [1, 2]. Compared with luminal type or HER2 type breast cancers, which showed distinct molecular markers for target therapy, TNBC is a group of breast cancers, which is a lack of effective therapeutic targets [3, 4]. Recent genome-wide association and biological studies have identified frequent abnormalities in Hippo signaling in TNBC samples. While manipulating on Hippo pathway could effectively change the biological behavior of TNBC, making it an promising targeting for TNBC therapy [5].

Hippo pathway is an evolutionally conserved pathway, which was firstly identified from *Drosophila* [6]. As a self-restricted pathway, Hippo pathway plays important functions in organ size control, tissue regeneration and homeostasis [7]. The core components of the Hippo signaling contain a series of phosphorylation kinases, including Set20-like kinase 1/2 (MST1/2), large tumor suppressor 1/2 (LATS1/2) and transcriptional co-factors YAP and TAZ [8]. When Hippo signaling is activated, MST1/2 facilitates the phosphorylation of LATS1/2, which subsequently promotes the phosphorylation of YAP/TAZ [9]. The phosphorylated YAP/TAZ could be retained in the cytosol and get degraded by a group of ubiquitin ligases [10]. However, if Hippo signaling is turned off, the un-phosphorylated YAP/TAZ could trans-locate into the nucleus and co-activate several transcriptional factors, such as TEADs, to activate a series of transcriptional programs [11].

The biological link between TNBC and the Hippo pathway has been established for decades [12]. For example, YAP expression is elevated in TNBC and is linked to poor overall survival [13]. The activation of YAP in TNBC could induce the expression of a set of genes in cell proliferation and anti-apoptosis in cell line assays [14]. As the important co-activator of the Hippo pathway, YAP could also cooperate with AP-1 family members Fra-1 to initiate the oncogenic transcriptional programs in TNBC cells [15], while YAP deletion could hamper the carcinogenic process in MMTV-PyMT mice models [16]. The reason for YAP hyper-activation remains uncertain, despite the continued functionality of the inhibitory kinase cascade, including MST1/2 and LATS1/2. Recent research suggests that modifications like methylation and ubiquitination may regulate the activity and stability of YAP in TNBC tumorigenesis [17, 18].

The interplay between E3 ubiquitin ligases and deubiquitinases regulates the stability of YAP protein and its transcriptome [19]. Through DUBs (Deubiquitinases) siRNA screening on TNBC cells, we discovered USP8 as a critical modulator to facilitate YAP function and TNBC progression. Inhibiting USP8 may be an effective approach for targeting Hippo-driven TNBC.

## Results

### USP8 serves as a crucial regulator of the Hippo signaling pathway in TNBC

**Fig. 1:**
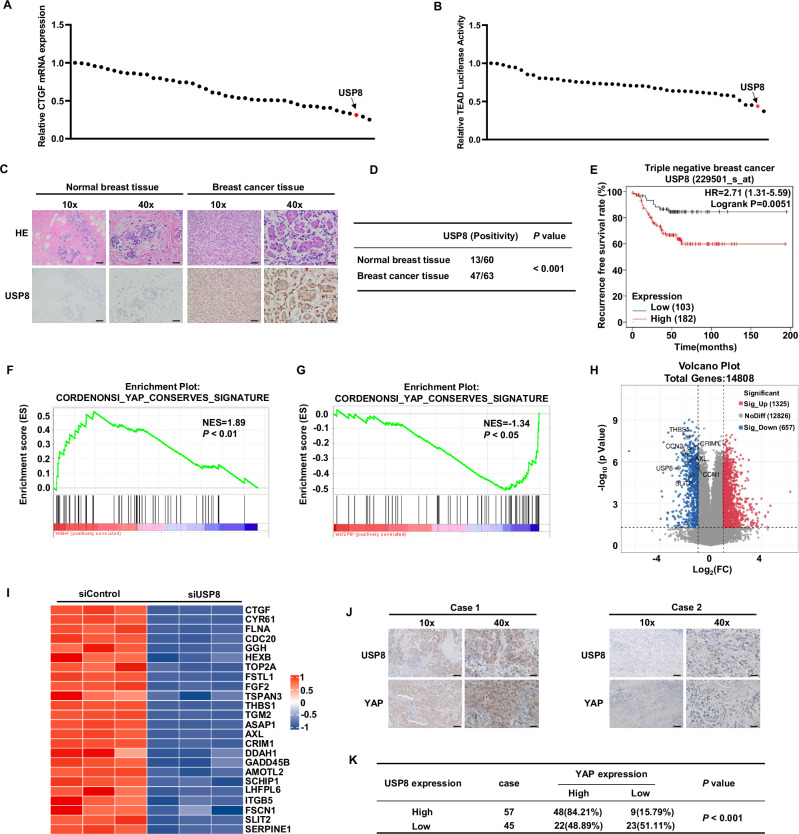
USP8 serves as a crucial regulator of the Hippo signaling pathway in TNBC. **A** After the genes of the deubiquitinase library were knocked out in BT549 cells, the expression level of YAP downstream target gene CTGF was detected by real-time PCR. **B** After the gene of the deubiquitinating enzyme library was knocked down in BT549 cells, luciferase reporter gene assay was used to detect the transcriptional activity of the transcription factor TEAD4. **C**, **D** Immunohistochemical analysis of USP8 expression in triple-negative and normal breast cancer tissue specimens. Statistical analysis of USP8 expression in 60 cases of normal breast tissue and 63 breast cancer samples. Scale bars, 100 μM (10X), 400 μM (40X). **E** Kaplan–Meier survival analysis of recurrence-free survival in triple-negative breast cancer patients with high and low expression of USP8 in KMPLOT database. **F** Analysis of the TCGA database using GSEA revealed a positive link between USP8 expression and YAP-targeted genes. **G** Enrichment analysis of gene sets from RNA-Seq data of siControl or siUSP8 in TNBC cell lines. **H** The volcano plot showed the changes in the expression of downstream target genes of the Hippo signaling pathway after treatment of TNBC cell lines with siControl or siUSP8. **I** Heatmap analysis showed the correlation between USP8 and YAP downstream target genes in RNA-Seq data. **J**, **K** Immunohistochemistry (IHC) staining of USP8 and YAP expression in TNBC specimens. Evaluation of statistical data on the association between USP8 and YAP expression in 102 breast cancer specimens. Scale bars, 100 μM (10X), 400 μM (40X). Unpaired two-tailed Student’s *t* tests were used to determine all P-values. *P < 0.05; **P < 0.01; ***P < 0.001 according to Student’s *t* test.

To identify novel deubiquitinases (DUBs) that regulate the Hippo signaling pathway, we utilized a DUBs siRNA library for screening in BT549 cells. Transfection was performed in BT549 cells, and the expression of CTGF, a classic downstream target gene of the Hippo pathway, as well as the transcriptional activity of TEAD, were monitored as indicators of Hippo signaling activity (Fig. S1A, B). Our screening identified known DUBs like USP7 and OTUB1, and uncovered others, such as USP8, that significantly influenced CTGF expression and the Hippo signaling pathway’s transcriptional activity (Fig. 1A, B). We further analyzed the expression of USP8 in TNBC by immunohistochemistry, which showed significantly elevated expression of USP8 in human TNBC (Fig. 1C, D). We explored the prognostic impact of USP8 in TNBC patients using the KM-plot database (https://kmplot.com/analysis/). The KM-plot analysis revealed a correlation between USP8 and low survival rates in TNBC (Fig. 1E). Gene set enrichment analysis (GSEA) of TNBC data in the TCGA database showed that there was a positive correlation between USP8 and Hippo signature genes. (Fig. 1F). Additionally, we performed RNA sequencing analysis after knocking down USP8 in BT549 cells. GSEA indicated that the absence of USP8 suppressed Hippo signaling activity (Fig. 1G). Volcano plots showed that USP8 deficiency downregulated several classic downstream target genes of the Hippo pathway. (Fig. 1H). Heatmaps demonstrated that the expression of Hippo target genes in BT549 cells was suppressed upon USP8 depletion (Fig. 1I). Furthermore, we conducted an analysis of USP8 and YAP expression in TNBC and found a positive correlation between their expressions in 102 clinical samples (Fig. 1J, K). Collectively, our findings suggest that USP8 is likely a positive regulator of the Hippo signaling pathway in TNBC.

### Knocking down USP8 inhibits the progression of TNBC

**Fig. 2:**
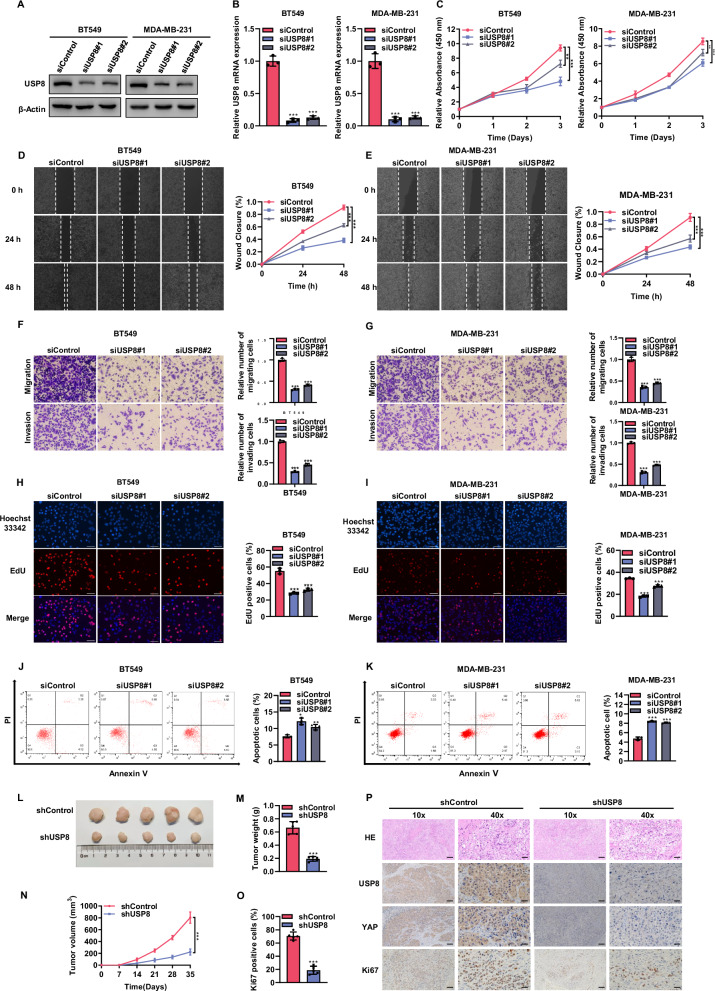
Knocking down USP8 inhibits the progression of TNBC. **A** Western blot showed the expression level of USP8 protein in BT549 or MDA-MB-231 cell lines transfected with siControl or two independent siUSP8 for 48 h. For internal control purposes, β-actin was employed. **B** RT-qPCR results of USP8 mRNA expression levels in BT549 or MDA-MB-231 cells transfected with siControl or siUSP8 for 48 h. **C** CCK8 assay was employed to determine the viability of BT549 or MDA-MB-231 cells received treatment with siControl or siUSP8 for 36 h at the indicated time point. **D**, **E** Wound healing assay was used to determine the migration ability of BT549 or MDA-MB-231 cell lines treated with siControl or siUSP8 for 36 h. The right panel shows the quantitative results of cell migration. **F**, **G** The Transwell assay was employed to assess the migration and invasion capabilities of BT549 or MDA-MB-231 cell lines treated with siControl or siUSP8 for 36 h. The right panel shows the quantitative results of cell migration and invasion. **H**, **I** EdU assay was used to determine the cell proliferation ability of BT549 or MDA-MB-231 cell lines treated with siControl or siUSP8 for 36 h. The right panel shows the quantitative results of cell proliferation. **J**, **K** Flow cytometry was used to determine the apoptosis level of BT549 or MDA-MB-231 cell lines treated with siControl or siUSP8 for 36 h. The quantitative outcomes of apoptosis are displayed in the right panel. **L**–**N** Four-week-old BALB/c female nude mice were subcutaneously injected with BT549 cells that stably expressed shControl and shUSP8. After 35 days, the mice were sacrificed, and the xenograft tumors were extracted. The images of tumors (**L**), their corresponding weight (**M**), and volume (**N**) are presented. **O**, **P** IHC staining shows the expression levels of USP8, YAP, and Ki67 in xenografts. The panel shows the quantitative results of Ki67. Scale bars, 100 μm (10X), 400 μm (40X). The results are displayed as mean ± SD, N = 3. *P < 0.05; **P < 0.01; ***P < 0.001.

We conducted a further investigation into the impact of USP8 on the phenotypic characteristics of TNBC. Western blot and RT-qPCR results demonstrated that USP8 was effectively silenced by two independent siRNAs (Fig. 2A, B). The CCK8 assay indicated that the suppression of USP8 led to a decrease in the proliferation of BT549 and MDA-MB-231 cells (Fig. 2C). In wound healing assays, we observed that the depletion of USP8 slowed the wound closure rate of BT549 and MDA-MB-231 cells (Fig. 2D, E). Transwell assays demonstrated that inhibiting USP8 decreased the migration and invasion abilities of BT549 and MDA-MB-231 cells (Fig. 2F, G). Moreover, EdU incorporation experiments indicated that the absence of USP8 decreased the number of EdU positive cells in BT549 and MDA-MB-231 cells (Fig. 2H, I). Flow cytometry (FACS) analysis indicated a rise in apoptotic cell numbers in BT549 and MDA-MB-231 cells after USP8 knockdown (Fig. 2J, K). Furthermore, we explored the in vivo role of USP8. Xenograft models demonstrated that the silence of USP8 significantly inhibited tumor growth (Fig. 2L, N). Immunohistochemistry results demonstrated reduced expression levels of YAP and Ki67 in tumors with the silence of USP8 (Fig. 2O, P). Collectively, these data indicate that USP8 plays a significant role in promoting the progression of TNBC.

### DUB-IN-2 inhibits the cell phenotype of TNBC cells

**Fig. 3:**
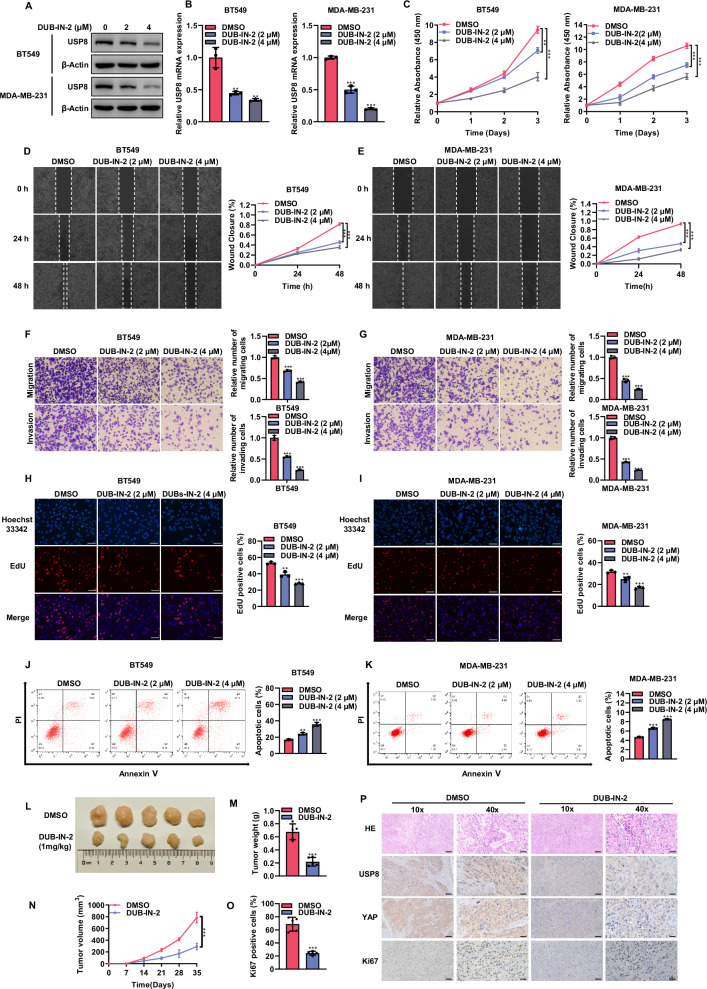
DUB-IN-2 inhibits the cell phenotype of TNBC cells. **A** Western blot showed the expression level of USP8 protein in BT549 or MDA-MB-231 cell lines treated with DMSO or different concentrations of DUBs-IN-2 for 8 h. To ensure internal control, β-actin was utilized. **B** RT-qPCR results of USP8 mRNA expression levels in BT549 or MDA-MB-231 cell lines treated with DMSO or different concentrations of DUBs-IN-2 for 8 h. **C** To evaluate cell viability, the CCK8 assay was conducted on BT549 or MDA-MB-231 cell lines exposed to DMSO or DUB-IN-2 for 12 h at the designated time. **D**, **E** Wound healing assay was used to determine the migration ability of BT549 or MDA-MB-231 cell lines treated with DMSO or DUB-IN-2 for 12 h. The right panel shows the quantitative results of cell migration. **F**, **G** Transwell assay was utilized to measure the migration and invasion proficiency of BT549 or MDA-MB-231 cell lines treated with DMSO or DUB-IN-2 for 12 h. The right panel shows the quantitative results of cell migration and invasion. **H**, **I** EdU assay was conducted to examine the proliferation of BT549 or MDA-MB-231 cell lines treated with DMSO or DUB-IN-2 for 12 h. The right panel shows the quantitative results of cell proliferation. **J**, **K** Flow cytometry was used to determine the apoptosis level of BT549 or MDA-MB-231 cell lines treated with DMSO or DUB-IN-2 for 12 h. The right panel shows the quantitative results of apoptosis. **L**–**N** BT549 cells were subcutaneously inoculated into 4-week-old BALB/c female nude mice treated with DMSO or DUB-IN-2 (1 mg/kg). Mice were euthanized 35 days post-injection, and xenograft tumors were excised. Displayed are representative tumor images (**L**), tumor weight (**M**), and tumor volume (**N**). **O**, **P** IHC staining shows the expression levels of USP8, YAP, and Ki67 in xenografts treated with DUB-IN-2. The panel shows the quantitative results of Ki67. Scale bars, 100 μm (10X), 400 μm (40X). All Data are shown as mean ± SD, N = 3. *P < 0.05; **P < 0.01; ***P < 0.001.

Small molecule inhibitors targeting the enzymatic activity of deubiquitinating enzymes (DUBs) have been developed and are entering preclinical studies or clinical trials [20, 21]. Recent studies have also indicated that DUB-IN-2 is an effective inhibitor of USP8 [22, 23]. Consequently, we conducted a series of experiments to investigate the impact of DUB-IN-2 on the phenotype of TNBC cells. Western blot and RT-qPCR validated the inhibitory effect of DUB-IN-2 on USP8 (Fig. 3A, B). The CCK8 assay results showed that DUB-IN-2 effectively suppressed the proliferation of BT549 and MDA-MB-231 cells (Fig. 3C). Additionally, this inhibitor slowed the wound closure rate of BT549 and MDA-MB-231 cells (Fig. 3D, E). Transwell assays revealed that DUB-IN-2 suppressed the migration and invasion abilities of BT549 and MDA-MB-231 cells (Fig. 3F, G). EdU incorporation experiments demonstrated a decrease in EdU-positive cells in BT549 and MDA-MB-231 cells following treatment with DUB-IN-2 (Fig. 3H, I). Additionally, we studied the impact of DUB-IN-2 on cell mortality. Flow cytometry results showed an increase in the number of apoptotic TNBC cells following treatment with the inhibitor (Fig. 3J, K). The absolute increase in cell apoptosis is biologically weak in USP8 inhibition, although it is statistically significant. We further evaluated the effect of USP8 on apoptosis under chemotherapy conditions. With the treatment of PTX, USP8 inhibition significantly increase the proportion of apoptotic cells. This might indicate USP8 could synergize with chemotherapy for TNBC treatment, which makes great importance for biological significance and clinical value (Fig. S2A, B). To evaluate the efficacy of DUB-IN-2, we implemented a xenograft mouse model. The findings indicated that DUB-IN-2 reduced the tumorigenic potential of breast cancer (Fig. 3L, N) and immunohistochemistry showed decreased Ki67 and YAP expression (Fig. 3O, P).

### Knockdown of USP8 leads to decrease Hippo signaling pathway activity in TNBC

**Fig. 4:**
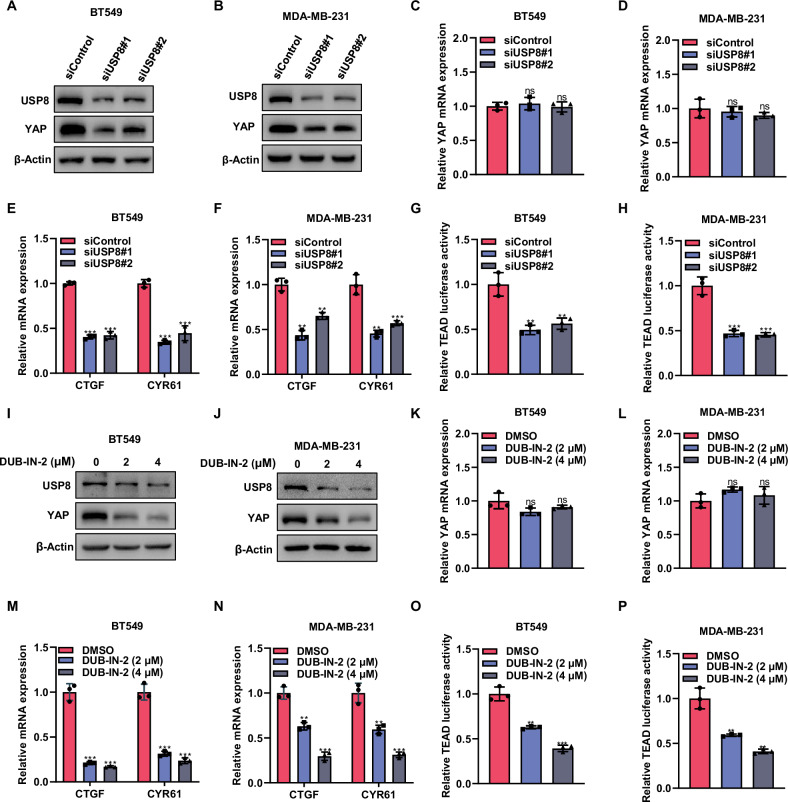
Knockdown of USP8 leads to decrease Hippo signaling pathway activity in TNBC. **A**, **B** Western blot showed the expression of USP8 and YAP protein in BT549 or MDA-MB-231 cells treated with siControl or siUSP8. As an internal standard, β-actin was utilized. **C**–**F** RT-qPCR showed the mRNA expression levels of YAP and downstream target genes CTGF and CYR61 in BT549 or MDA-MB-231 cells treated with siControl or two independent siRNAs. **G**, **H** Luciferase reporter assay of TEAD transcriptional activity in BT549 or MDA-MB-231cells treated with siControl or siUSP8. **I**, **J** Western blot showed the expression of USP8 and YAP protein in BT549 or MDA-MB-231 cells treated with DMSO or different concentrations of DUB-IN-2. β-actin functioned as the internal control. **K**–**N** RT-qPCR showed the mRNA expression levels of YAP and downstream target genes CTGF and CYR61 in BT549 or MDA-MB-231 cells treated with DMSO or different concentrations of DUB-IN-2. **O**, **P** Luciferase reporter assay of TEAD transcriptional activity in BT549 or MDA-MB-231cells treated with DMSO or different concentrations of DUB-IN-2. All Data are shown as mean ± SD, N = 3. *P < 0.05; **P < 0.01; ***P < 0.001.

Given the central role of YAP in the Hippo signaling pathway, we further explored the effects of USP8 on YAP protein. USP8 depletion decreased YAP protein expression without altering YAP mRNA levels (Fig. 4A–D). This implies that USP8 might modulate YAP through post-translational mechanisms. Moreover, qPCR data showed that the silencing of USP8 suppressed the expression of downstream target genes of YAP, such as CTGF and CYR61 (Fig. 4E, F). Additionally, luciferase assays indicated that the inhibition of USP8 also reduced Hippo signaling activity (Fig. 4G, H). Similar results were obtained when using a USP8 inhibitor. After treatment with DUB-IN-2, the protein expression levels of YAP in BT549 and MDA-MB-231 cells decreased (Fig. 4I, L). The downstream target genes of YAP also showed decreased expression (Fig. 4M, N), and the activity level of the Hippo pathway was reduced (Fig. 4O, P). The findings indicate a connection between USP8 and Hippo signaling pathway activity in triple-negative breast cancer.

### Overexpression of YAP reduces the effects of USP8 suppression in TNBC

**Fig. 5:**
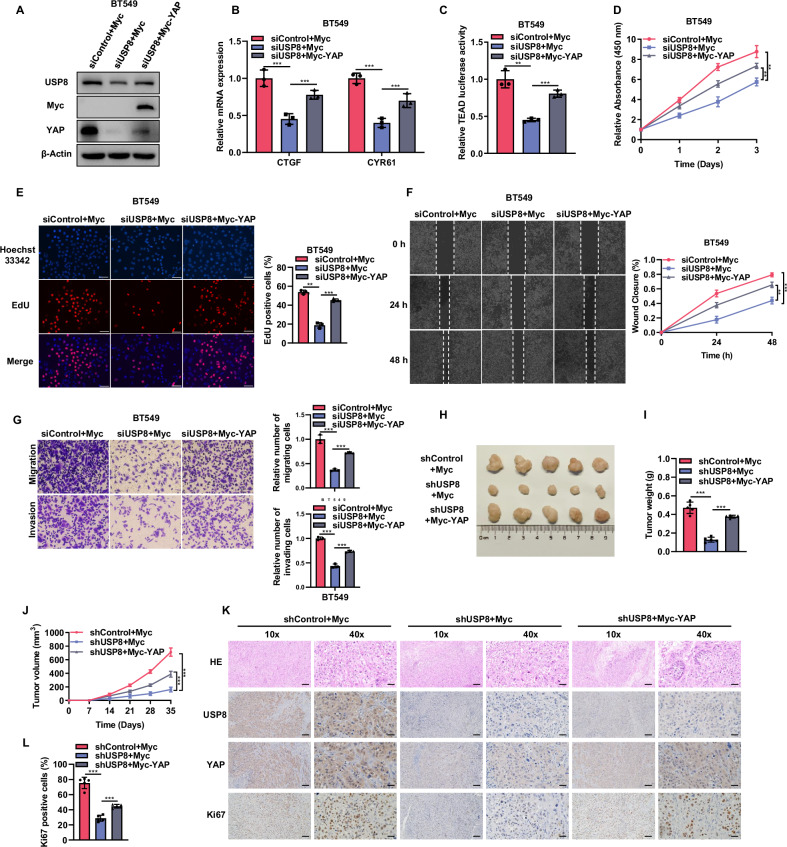
Overexpression of YAP reduces the effects of USP8 suppression in TNBC. **A** Western blot showed that the expression levels of USP8 and YAP proteins in BT549 cells transfected with Myc or Myc-YAP plasmids after treatment with siUSP8. The internal control in the experiment was β-actin. **B** RT-qPCR showed the mRNA expression levels of CTGF and CYR61 in BT549 cells transfected with Myc or Myc-YAP plasmid after treatment with siUSP8. **C** Luciferase reporter assay of the transcriptional activity of TEAD transfected with Myc or Myc-YAP plasmid after BT549 cells were treated with siUSP8. **D** The CCK8 assay determined the viability of BT549 cells transfected with Myc or Myc-YAP after siUSP8 treatment at a specific time. **E** The proliferation ability of BT549 cells transfected with Myc or Myc-YAP plasmid after siUSP8 treatment was assessed by EdU. The right panel shows the quantitative results of cell proliferation. **F** The migration ability of BT549 cells transfected with Myc or Myc-YAP plasmid after siUSP8 treatment was detected using a wound healing assay. The quantitative findings of cell proliferation are presented in the right panel. **G** The Transwell assay was employed to assess the migration and invasion capabilities of BT549 cells transfected with Myc or Myc-YAP plasmid following siUSP8 treatment. The right panel shows the quantitative results of cell migration and invasion. **H**–**J** BT549 cells stably expressing shControl and shUSP8 or YAP were subcutaneously inoculated into 4-week-old BALB/c female nude mice. Mice were euthanized 35 days post-injection, and the xenograft tumors were excised. Images of representative tumors (**H**), along with their weight (**I**) and volume (**J**), are displayed. **K**, **L** IHC staining shows the expression levels of USP8, YAP, and Ki67 in xenografts. The panel shows the quantitative results of Ki67. Scale bars, 100 μm (10X), 400 μm (40X). Data are presented as mean ± SD, with N = 3. *P < 0.05; **P < 0.01; ***P < 0.001.

We further conducted rescue experiments to determine whether USP8 promotes the progression of TNBC through YAP. According to Western blot analysis, YAP overexpression can counteract the reduction in its expression levels caused by USP8 knockdown. (Fig. 5A). qPCR data and luciferase assay indicated that overexpression of YAP could rescue the decreased Hippo pathway activity caused by USP8 silencing (Fig. 5B, C). CCK8 and EdU incorporation experiments revealed that the depletion of USP8 suppresses breast cancer cell proliferation, which can be partially counteracted by YAP overexpression (Fig. 5D, E). In wound healing assays, overexpression of YAP restored the wound closure rate of BT549 cells (Fig. 5F). Transwell assays showed that overexpression of YAP could also rescue the reduced migratory and invasive capabilities of BT549 cells caused by USP8 deficiency (Fig. 5G). In xenograft mouse models, the depletion of USP8 inhibited tumor growth, an effect that could be rescued by overexpressing YAP (Fig. 5H–J). Immunohistochemistry of tumor specimens showed that overexpression of YAP partially rescued the reduced expression of YAP and Ki67 caused by USP8 depletion (Fig. 5K, L). These data suggest that USP8 promotes the progression of breast cancer through YAP.

### USP8 maintains YAP protein stability by interacting with YAP in TNBC

**Fig. 6:**
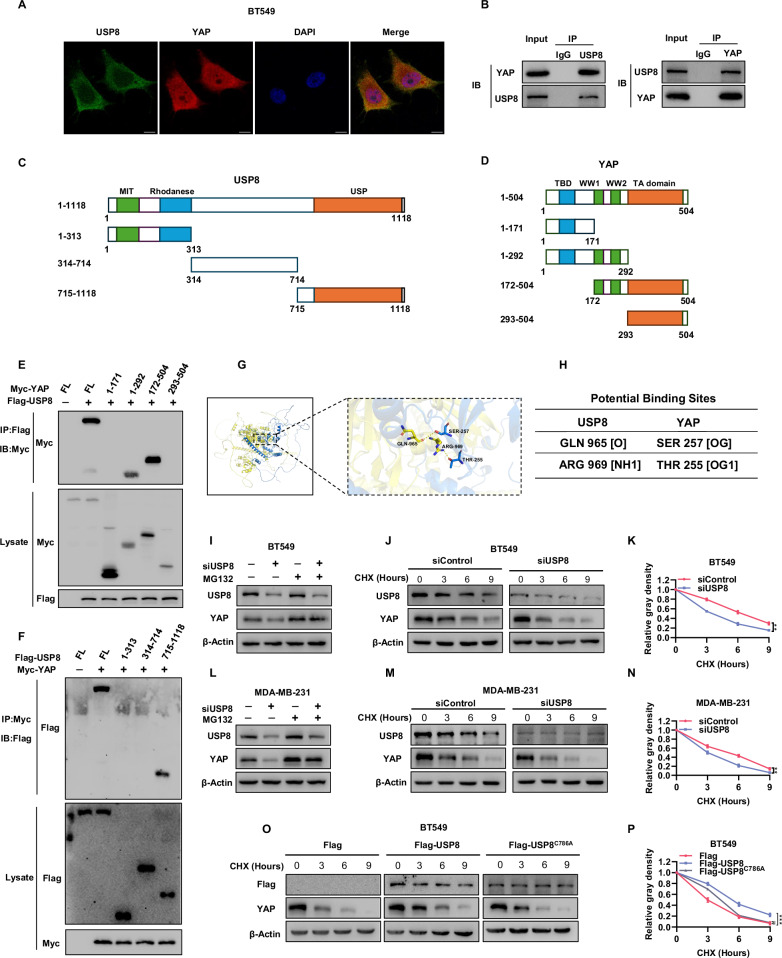
USP8 maintains YAP protein stability by interacting with YAP in TNBC. **A** Immunofluorescence staining showed the localization of USP8 (green) and YAP (red) in BT549 cells. DAPI (blue) was used to stain the nuclei, and the scale bars indicate 10 μm. **B** An endogenous association between USP8 and YAP was demonstrated through immunoprecipitation analysis. To investigate endogenous interaction between USP8 and YAP, BT549 cell lysates were precipitated using anti-YAP or anti-USP8 antibodies, and the resulting precipitate was analyzed through immunoblotting. **C** Diagram illustrating the protein structure of UPS8 and its deletion mutants (residues 1–313, 314–714, 715–1118) utilized in the co-immunoprecipitation assay. **D** A schematic diagram of the YAP protein structure and its deletion mutants (residues 1–171, 1–292, 172–504, 293–504) used for the CO-IP assay. **E** Co-immunoprecipitation revealed that the interaction of the WW domain of YAP with USP8. **F** Co-immunoprecipitation demonstrated that YAP interaction necessitates the USP domain of USP8. **G** Surface diagram of the docking model and their interfacing residues between USP8 and YAP protein (YAP, blue; USP8, yellow; hydrogen bond interaction, dotted line). **H** The two sites of USP8 are mutated to alanine, and the table shows the predicted binding sites of the two proteins. **I**, **L** Western blot analysis showed that the expression level of YAP protein in BT549 and MDA-MB-231 cells. Cells were transfected with siControl or siUSP8 and then treated with MG132. **J**, **K**, **M**, **N** Western blotting was used to detect the protein levels of USP8 and YAP. For the specified time, BT549 and MDA-MB-231 cells received treatment with 100 μM cyclohexylamine (CHX). The YAP protein expression was measured with ImageJ software and illustrated in the right panel. **O**, **P** Evaluation of YAP half-life in BT549 cells following transfection with the specified plasmids. The right panel graphically displays the YAP protein expression as estimated by ImageJ software.

Subsequently, we further investigated the localization of USP8 and YAP in TNBC cells. Immunofluorescence results showed that USP8 is primarily localized in the cytoplasm, while YAP is localized in both the cytoplasm and nucleus (Fig. 6A). Further endogenous co-immunoprecipitation experiments indicated that USP8 can interact with YAP in BT549 cells (Fig. 6B). USP8 is composed of the MIT domain, Rhodanase domain, and USP domain (Fig. 6C), while the YAP protein consists of the TBD domain, WW domain, and TA domain (Fig. 6D). We created deletion mutants of both USP8 and YAP to better understand the domains involved in their interaction. The study revealed that the USP domain of USP8 interacts with the WW domain of YAP. (Fig. 6E, F). Given the confirmed interaction between USP8 and YAP, we used rigid protein-protein docking to predict the binding sites of the interacting domains (Fig. 6G). As predicted, USP8 binds to YAP through residues 956 and 969 of USP8 and residues 257 and 255 of YAP (Fig. 6H). Given the established interaction between USP8 and YAP, and the observed reduction in YAP protein levels upon USP8 depletion, we hypothesized that USP8 regulates YAP protein via the ubiquitin-proteasome pathway. Interestingly, we discovered that USP8 depletion decreases YAP protein levels, an effect mitigated by MG132 in BT549 and MDA-MB-231 cells. (Fig. 6I, L). Protein stability assays with cycloheximide, which inhibits protein synthesis, demonstrated that USP8 depletion decreases the half-life of YAP protein in BT549 and MDA-MB-231 cells. (Fig. 6J, K, M, N). The necessity of USP8’s enzymatic activity for maintaining YAP protein levels was demonstrated by overexpressing the USP8 catalytic activity mutant (C786A) in BT549 cells. (Fig. 6O, P). The data indicate that USP8’s deubiquitinating enzyme activity is crucial for stabilizing YAP protein.

### USP8 stabilizes YAP by reducing K48 poly-ubiquitination

**Fig. 7:**
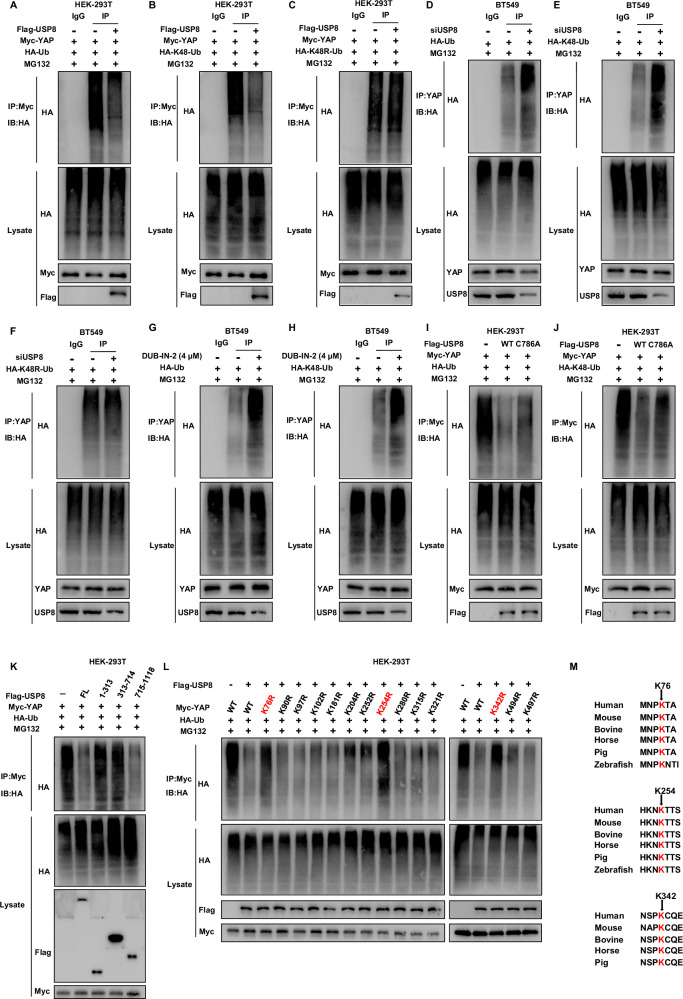
USP8 stabilizes YAP by reducing K48 poly-ubiquitination. **A** Overexpression of USP8 reduces the ubiquitination of YAP protein. The ubiquitination status of YAP was assessed via immunoblotting utilizing specific antibodies. **B**, **C** Overexpression of USP8 reduces the ubiquitination of K48-linked YAP protein. The level of K48/K48R-specific ubiquitination of YAP was assessed through immunoblotting using designated antibodies. **D** Knockdown of USP8 increases the total ubiquitination of YAP protein. The total ubiquitination level of YAP was assessed by immunoblotting. Subsequently, the cells were analyzed using designated antibodies. **E**, **F** Knockdown of USP8 increases the ubiquitination of K48-linked YAP protein. Immunoblotting was used to determine the K48/K48R-specific ubiquitination level of YAP. **G** The USP8 inhibitor DUB-IN-2 increases the total ubiquitination of YAP protein. The ubiquitination level of YAP was measured through immunoblotting. **H** The USP8 inhibitor DUB-IN-2 increases the ubiquitination of K48-linked YAP protein. To determine the K48-specific ubiquitination level of YAP, immunoblotting was performed. **I** USP8 WT, but not the enzyme-inactivating USP8 C786A mutant, reduces the total ubiquitination of YAP protein. To measure the total ubiquitination of YAP, immunoblotting was performed on HEK-293T cells. **J** USP8 WT, but not the enzyme-inactivating USP8 C786A mutant, reduces the K48-linked ubiquitination of YAP protein. The level of K48-specific ubiquitination of YAP was assessed through immunoblotting. **K** The USP8 USP domain (714–1118) decreases the ubiquitination of the YAP protein. The overall level of YAP ubiquitination was determined via immunoblotting. The cells were subjected to immunoblotting with designated antibodies. **L** USP8 can ubiquitinate YAP at the K76, K254, and K342 sites of the YAP protein. The ubiquitination assay showed that YAP was ubiquitinated at multiple predetermined sites. **M** Cross-species analysis of K76, K254 and K342 on YAP protein was performed in multiple species.

We further investigated the effect of USP8 on YAP ubiquitination. Initially, we employed ubiquitination-based co-immunoprecipitation experiments to demonstrate that USP8 inhibits the overall polyubiquitination levels and K48-linked ubiquitination of YAP (Fig. 7A, B), but does not affect the K63-linked ubiquitination of YAP (Fig. S3A). Moreover, the K48R dominant-negative mutant of ubiquitin is capable of reducing the impact of USP8 on YAP ubiquitination (Fig. 7C), while K63R can still deubiquitinate YAP (Fig. S3B). Subsequently, we examined the impact of USP8 on YAP polyubiquitination in BT549 cells. The results showed that the depletion of USP8 increased the overall polyubiquitination and K48-linked ubiquitination levels of YAP (Fig. 7D, E), but did not alter the K63-linked ubiquitination levels (Fig. S3C). Concurrently, the ubiquitin dominant-negative mutant (K48R) can weaken the effect of USP8 on YAP ubiquitination (Fig. 7F), while K63R can still deubiquitinate YAP (Fig. S3D). Building on prior research highlighting DUB-IN-2’s selective inhibition of USP8, we employed DUB-IN-2 to evaluate its effect on YAP polyubiquitination. Endogenous co-immunoprecipitation coupled with ubiquitin signal immunoblotting indicated that treatment of BT549 cells with the drug increased the overall polyubiquitination and K48-linked ubiquitination levels of YAP (Fig. 7G, H), but did not change its K48R dominant ubiquitination levels (Fig. S3E). The K63-linked ubiquitination cannot inhibit the effect of USP8 on YAP ubiquitination (Fig. S3F), while K63R can still deubiquitinate YAP (Fig. S3G). Furthermore, we examined the effect of wild-type and USP8 catalytic activity mutant (C786A) on YAP polyubiquitination. The study demonstrated that USP8’s catalytic activity is essential for K48-linked ubiquitination of YAP. (Fig. 7I, J and S3H–J). Concurrently, domain-based ubiquitination analysis indicated that USP8 requires its USP domain to deubiquitinate YAP (Fig. 7K and S3K, L). Since the YAP protein contains 14 lysine sites, we generated these mutants. Ubiquitination experiments showed that USP8 can inhibit the polyubiquitination of YAP protein at the K76, K254, and K342 sites (Fig. 7L). Moreover, among these sites, the K76, K254, and K342 sites are evolutionarily conserved from zebrafish to humans (Fig. 7M). Collectively, these results indicate that USP8 inhibits K48-linked polyubiquitination of YAP but has minimal impact on K63-linked polyubiquitination.

### A positive feedback mechanism exists between USP8 and YAP, with YAP enhancing the expression of USP8

**Fig. 8:**
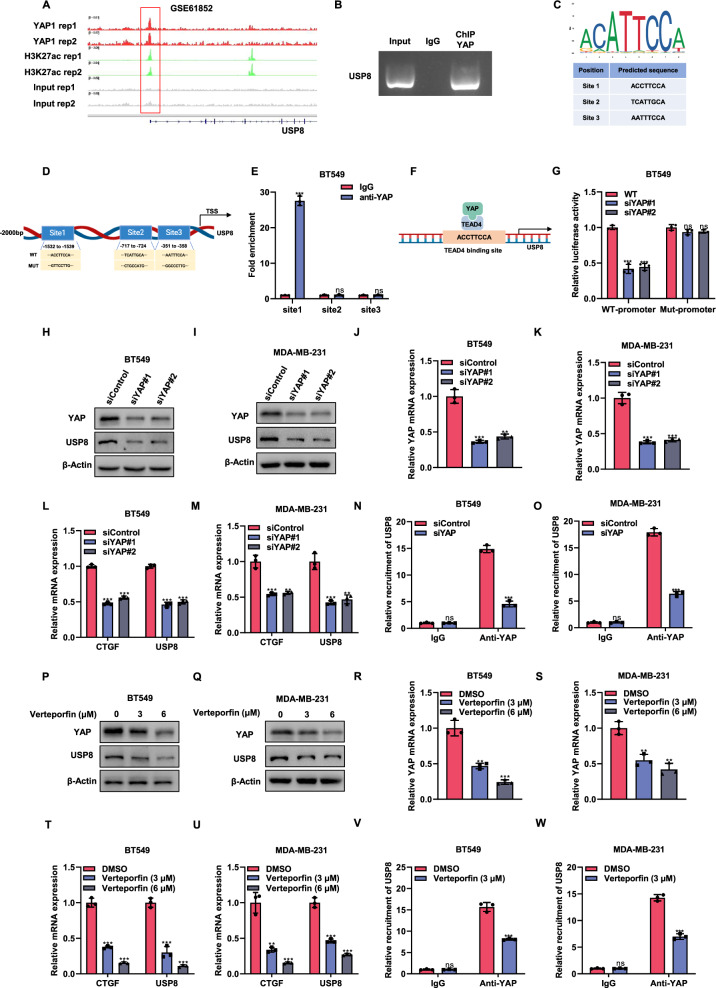
A positive feedback mechanism exists between USP8 and YAP, with YAP enhancing the expression of USP8. **A** The analysis of YAP binding to the USP8 promoter region via ChIP-seq employed GEO data with accession numbers GSE61852. **B** ChIP-PCR analysis in BT549 cells shows that YAP was concentrated at the USP8 promoter. **C** Predicted TEAD4 binding sequences and sites in the USP8 promoter according JASPAR. **D** Schematic of the hypothesized TEAD-binding site in the USP8 promoter and the primers employed for chromatin immunoprecipitation. The sequences shown include the wild-type (WT) and two mutated (Mut) USP8 promoter luciferase constructs. **E** ChIP-qPCR was performed on BT549 cells with an anti-YAP antibody and an IgG control. **F** The schematic diagram of YAP and TEAD4 complex binding to the USP8 promoter. **G** Luciferase reporter gene assay for USP8 promoter activity in WT and YAP knockdown cells transfected with wild-type or mutant TEAD binding sites. **H**, **I** Western blot showed the expression of USP8 and YAP protein in BT549 or MDA-MB-231 cells treated with siControl or siYAP. The internal control in the experiment was β-Actin. **J**–**M** RT-qPCR showed the mRNA expression level of USP8, YAP and CTGF in BT549 cells or MDA-MB-231 cells treated with siControl or two independent siRNAs. **N**, **O** The binding of YAP to the USP8 promoter was detected through a ChIP assay. YAP depletion in BT549 and MDA-MB-231 cells led to decreased binding to the USP8 gene, as demonstrated by ChIP-qPCR assays. **P**, **Q** Western blot showed the expression of USP8 and YAP protein in BT549 or MDA-MB-231 cells treated with DMSO or different concentrations of Verteporfin. β-Actin served as the internal control. **R**–**U** RT-qPCR showed the mRNA expression level of USP8, YAP and CTGF in BT549 or MDA-MB-231 cells treated with DMSO or different concentrations of Verteporfin. **V**, **W** Enhanced binding of YAP to the USP8 promoter was observed in BT549 and MDA-MB-231 cells following a 6-h treatment with Verteporfin, as demonstrated by ChIP-qPCR analysis. All data are presented as mean ± SD, with N = 3. *P < 0.05; **P < 0.01; ***P < 0.001.

A number of studies examine the genomic binding sites of YAP in breast cancer, highlighting its pivotal role in breast cancer progression [24]. Analysis of YAP ChIP sequencing data (GSE61852) revealed a significant peak of binding in the US8 promoter region, demonstrating that YAP also plays a role in the regulation of USP8 expression (Fig. 8A). ChIP experiments were conducted to validate the interaction between YAP protein and the USP8 gene promoter region (Fig. 8B). YAP functions as a transcriptional co-activator by interacting with TEAD family transcription factors to regulate target gene expression, as it cannot directly bind to DNA. We investigated the potential transcriptional regulation of USP8 by TEAD first, using the JASPAR database, we predicted three TEAD4 binding sites (site 1, site 2, site 3) in the promoter region of USP8 (Fig. 8C). We designed three pairs of primers based on the predicted binding sites, and ChIP-qPCR showed that YAP can bind to site 1 of the USP8 promoter (Fig. 8D, E). Luciferase reporter assays demonstrated that reducing YAP levels led to a decrease in luciferase activity of the USP8 promoter with the wild-type binding site 1, whereas the mutated site 1 showed no such reduction (Fig. 8F, G). Moreover, reducing YAP in BT549 and MDA-MB-231 cells resulted in lower protein and mRNA levels of USP8. (Fig. 8H–M). To further confirm YAP-driven transcriptional activation of USP8, YAP was silenced in BT549 and MDA-MB-231 cells. ChIP-qPCR confirmed that the depletion of YAP reduced the binding of YAP to the USP8 gene (Fig. 8N, O). Previous studies have shown that the small molecule inhibitor verteporfin can inhibit the function of YAP. After treatment with verteporfin, the protein and mRNA levels of USP8 in BT549 and MDA-MB-231 cells were reduced (Fig. 8P–U). ChIP-qPCR experiments showed a reduced binding of YAP to the USP8 gene (Fig. 8V, W). The data imply that YAP regulates USP8 expression through transcription, resulting in a positive feedback mechanism.

### Inhibition of USP8 enhances the sensitivity of TNBC cells to chemotherapeutic agents

**Fig. 9:**
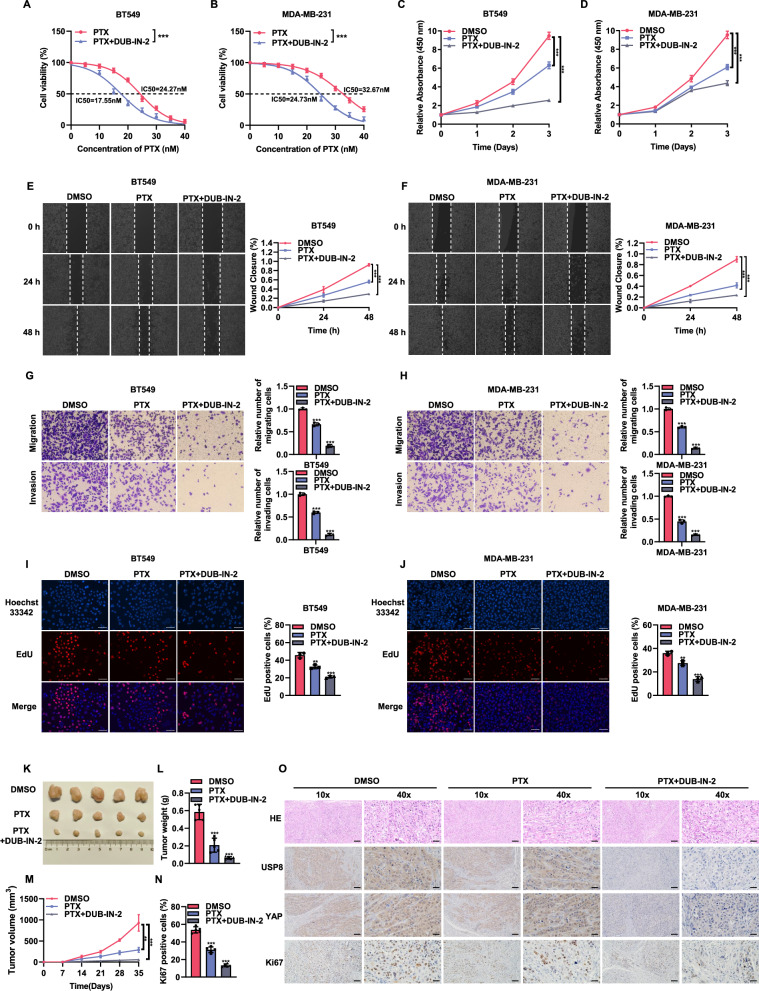
Inhibition of USP8 enhances the sensitivity of TNBC cells to chemotherapeutic agents. **A**, **B** DUB-IN-2 can enhance the sensitivity to PTX treatment. BT549 and MDA-MB-231 cells underwent a 24-h treatment with varying concentrations of PTX, followed by an MTT assay. The IC50 values for PTX were presented for each group. **C**, **D** Treatment of cells with the combination of DUB-IN-2 and PTX can inhibit cell proliferation. CCK8 assay was used to determine the viability of BT549 or MDA-MB-231 cells transfected with DMSO, PTX or DUB-IN-2 at the indicated time point. **E**, **F** Treatment of cells with the combination of DUB-IN-2 and PTX can inhibit cell migration. Wound healing assay was used to determine the migration ability of BT549 or MDA-MB-231 treated with DMSO, PTX or DUB-IN-2. The right panel shows the quantitative results of cell proliferation. **G**, **H** Treatment of cells with the combination of DUB-IN-2 and PTX can inhibit cell migration and invasion. Transwell assay was used to detect the migration and invasion ability of BT549 or MDA-MB-231cell line treated with DMSO, PTX or DUB-IN-2. **I**, **J** Treatment of cells with the combination of DUB-IN-2 and PTX can inhibit cell proliferation. The EdU assay was employed to assess the proliferation capacity of BT549 or MDA-MB-231 treated with DMSO, PTX or DUB-IN-2. The right panel shows the quantitative results of cell proliferation. **K**–**M** Treatment of cells with the combination of DUB-IN-2 and PTX can inhibit cell proliferation in vivo. BT549 cells were subcutaneously inoculated into 4-week-old BALB/c female nude mice treated with DMSO and PTX or DUB-IN-2. Mice were euthanized 35 days post-injection, and the xenograft tumors were excised. Images of representative tumors (**K**), along with their weight (**L**) and volume (**M**), are displayed. **N**, **O** The expression levels of USP8, YAP, and Ki67 in xenograft models treated with PTX or DUB-IN-2 were visualized using IHC staining. The panel shows the quantitative results of Ki67. Scale bars, 100 μm (10X), 400 μm (40X). Data are represented as the average ± SD, based on N = 3 independent experiments. *P < 0.05; **P < 0.01; ***P < 0.001.

In the clinical setting, the mainstay of treatment for TNBC patients is chemotherapy [25]. Specifically, paclitaxel, a microtubule stabilizer, is a first-line chemotherapeutic agent for TNBC patients in the clinic [26]. Nevertheless, many TNBC patients experience resistance, resulting in treatment failure [27]. Therefore, we investigated the combined use of the small molecule inhibitor DUB-IN-2 and paclitaxel to explore their impact on the phenotype of TNBC. As observed in the IC50 determination, the use of DUB-IN-2 reduced the inhibitory IC50 of paclitaxel (Fig. 9A, B). CCK8 experiments indicated that the use of the inhibitor DUB-IN-2 enhanced the inhibitory effect of paclitaxel on BT549 and MDA-MB-231 cells (Fig. 9C, D). In wound healing experiments, the inhibitory effect of the combined treatment on the wound closure rate of breast cancer cells was more pronounced (Fig. 9E, F). Transwell experiments showed that the combined use of paclitaxel and the inhibitor reduced the migratory and invasive capabilities of breast cancer cells (Fig. 9G, H). EdU incorporation experiments indicated a decrease in the number of EdU positive cells in breast cancer cells treated with paclitaxel and DUB-IN-2 (Fig. 9I, J). We employed a xenograft mouse model to examine the combined effects of paclitaxel and DUB-IN-2 on breast cancer aggressiveness. The results showed that the combined use of paclitaxel and DUB-IN-2 more effectively inhibited tumor growth in vivo (Fig. 9K, M) and also reduced the expression of Ki67 in tumors (Fig. 9N, O). The above data indicate that the inhibition of USP8 enhances the sensitivity of TNBC cells to chemotherapeutic drugs.

**Fig. 10:**
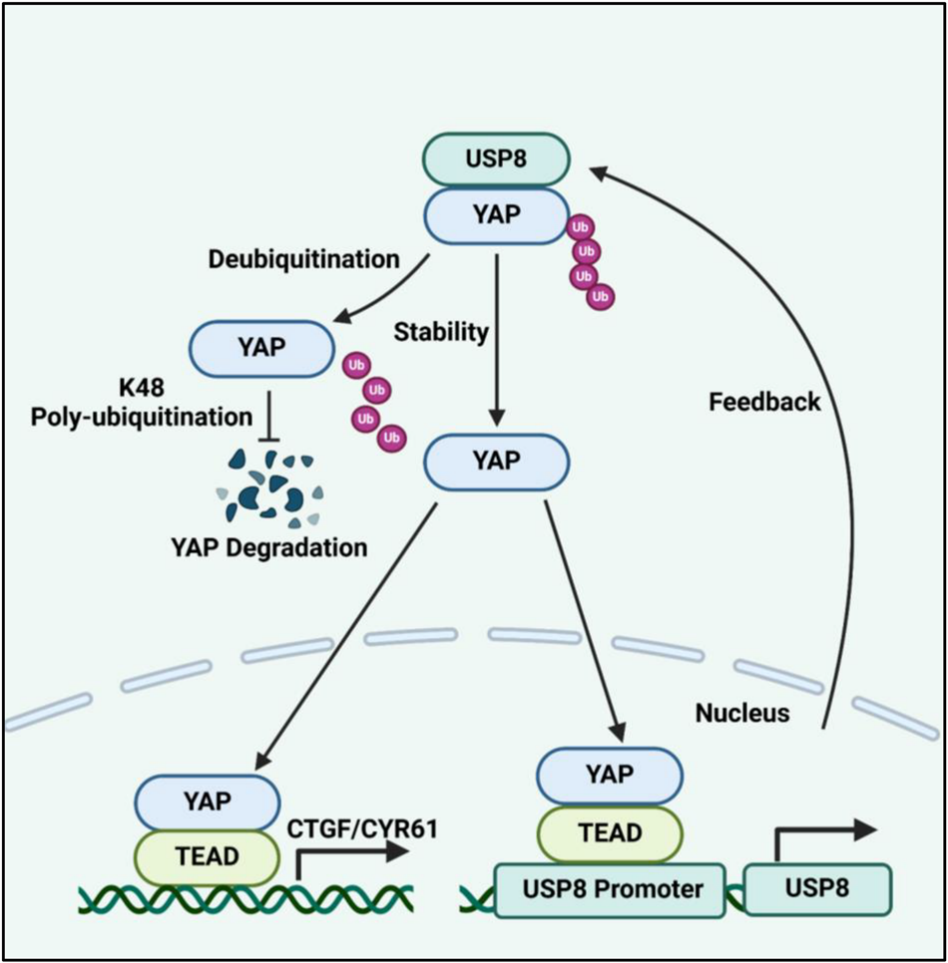
A hypothetical model of USP8 regulated Hippo signaling mechanism and the positive feedback loop between USP8 and YAP in TNBC. The activation of USP8 enhances Hippo signaling activity and promotes the progression of breast cancer by deubiquitinating YAP K48 poly-ubiquitination. Furthermore, YAP is capable of inducing USP8 expression, which points to a positive feedback loop with Hippo signaling.

Collectively, our work demonstrates that USP8 promotes TNBC progression by acting on the Hippo/YAP axis. USP8 activation deubiquitinates YAP at K48-linked ubiquitination, leading to enhanced Hippo signaling and accelerated breast cancer progression. Furthermore, YAP can induce the expression of USP8, suggesting a positive feedback loop operates between USP8 and the Hippo/YAP axis (Fig. 10).

## Discussion

Our current study identified an unexpected regulation loop between USP8 and Hippo/YAP axis in promoting TNBC progression (Fig. 10). In TNBC samples, USP8 expression was positively associated with YAP protein levels and inversely related to overall survival in patients. By preventing K48-linked poly-ubiquitination and degradation of YAP, USP8 could stabilize the YAP protein, promoting the progression of YAP-driven TNBC. On the other hand, YAP could bind to the promoter region of the USP8 gene and facilitate its transcription. The regulation loop revealed a novel insight how the Hippo/YAP axis coordinated with USP8 in promoting TNBC progression. USP8 may act as both an upstream regulator and a downstream target of Hippo signaling in TNBC, positioning it as a crucial modulator. Inhibiting USP8 may represent a potential therapeutic strategy for treating TNBC progression.

USP8 (Ubiquitin Specific Proteases 8) belongs to the family of ubiquitin-specific proteases, which is composed of 1118 amino acids with the hydrolysis domain in the C-terminus [28]. Quite a few studies showed that USP8 could deubiquitinate and stabilize several targets. For example, USP8 may stabilize the type II TGF-β receptor, thereby enhancing TGF-SMAD axis activity and promoting tumor invasion [29]. Besides, USP8 could regulate mitophagy process via modulating Parkin activity, while pharmacological targeting USP8 could rescue the phenotype of Parkinson’s disease [30, 31]. USP8 also play important role in several human malignancies via modulating several oncogenic proteins, including MET and EGFR [32, 33]. Interestingly, our unbiased DUB screening also revealed that USP8 is also an important modulator in the Hippo pathway in TNBC, which also add knowledge for its role in cancer biology, while targeting USP8 could block several oncogenic pathways and be of great significance in cancer therapeutics. However, the therapeutic targeting of USP8 presents a double-edged sword. As a crucial regulator of EGFR sorting and degradation, USP8 inhibition potently suppresses EGFR signaling (a promising strategy for EGFR-driven cancers) but may simultaneously induce epidermal and gastrointestinal toxicities, such as rash, itching, dry skin, oral mucositis, diarrhea, and anorexia. Moreover, given its role in modulating multiple receptor tyrosine kinases (RTKs), USP8 inhibition could disrupt hormonal feedback circuits, including cortisol and ACTH axes, and may lead to additional off-target effects such as immune suppression, headaches, and hematological toxicity. These adverse effects are not unique to USP8 inhibition but are also commonly observed with other targeted therapies (e.g., TKIs), immunotherapies, and chemotherapy.

In this study, we used DUB-IN-2, an irreversible inhibitor that covalently binds the catalytic cysteine of USP8, to probe its function. Although structural similarities among deubiquitinases raise the possibility of off-target inhibition, our functional validation using USP8 depletion and overexpression confirms that USP8 authentically regulates the Hippo pathway in TNBC. While the final verification of clinical application value still requires support from future clinical trials, multiple key experimental results in Fig. 9 indicate that the sensitizing effect mediated by DUB-IN-2 has potential clinical significance: the combination therapy of paclitaxel (PTX) and DUB-IN-2 can enhance the sensitivity of PTX treatment. Ultimately, the translational promise and safety profile of USP8 inhibition must be rigorously evaluated in future clinical trials to determine its potential for clinical application.

For decades, the crucial roles of the Hippo/YAP pathway in TNBC have been identified. Given that most TNBC samples exhibit over-activation of the Hippo/YAP axis, blocking the YAP/TEAD interaction appears to be a potential therapeutic approach for cancers driven by this pathway [34, 35]. Pharmaceutical drugs like verteporfin and Super-TDU, intended to inhibit the Hippo/YAP axis, did not succeed in clinical translation during various pre-clinical studies on Hippo-driven cancer [36]. Various factors may explain the ineffectiveness of verteporfin and Super-TDU [36, 37]. A critical concern is cell membrane penetration, as inhibitors must traverse the membrane to obstruct cytosolic protein interactions [38, 39]. Thus, we shift our strategy into identifying novel modulator of Hippo/YAP axis in TNBC. Interestingly, as the specific inhibitor of USP8, DUB-IN-2 is capable of preventing TNBC from proliferating and metastasizing in both live models and controlled lab conditions. Importantly, DUB-IN-2 could synergize with paclitaxel to inhibit TNBC cells, which indicate that targeting USP8 could be a potential tactic to combat chemotherapy resistance in TNBC patients. Interestingly, our examination of high-resolution IHC images revealed that in the YAP high-expression group, the staining, although intense, frequently displayed a diffuse cytoplasmic and nuclear distribution rather than exclusive nuclear localization. This pattern suggests that YAP abundance alone may not fully correlate with transcriptional activity, and that its nuclear translocation and activation are likely regulated by additional mechanisms. In this context, we identified USP8 as a key regulator of the Hippo pathway. The specific USP8 inhibitor DUB-IN-2 effectively suppressed TNBC proliferation and metastasis in both in vitro and in vivo models. Importantly, DUB-IN-2 synergized with paclitaxel to enhance anti-tumor efficacy, indicating that targeting USP8 may represent a viable therapeutic tactic to overcome chemotherapy resistance in TNBC patients.

We discovered a new positive feedback loop between USP8 and the Hippo/YAP axis in TNBC progression, suggesting that inhibiting USP8 may effectively target the Hippo/YAP axis and combat chemotherapy resistance in TNBC patients.

## Materials and methods

### Cell lines and cell culture

The Human triple negative breast cancer BT549, MDA-MB-231 and the human embryonic kidney HEK-293T cells were acquired from American Type Culture Collection (ATCC). MDA-MB-231 and HEK-293T cells were cultured in Dulbecco’s modified Eagle’s medium (6124056, Gibco). BT549 cells were cultured in RPMI-1640 medium (R8758, Sigma-Aldrich). All cell lines were supplemented with 10% fetal bovine serum (FBS,10270-106, Gibco) and 1% penicillin/streptomycin (C0223, Beyotime). They were maintained at 37 °C and passaged and trypsinized according to the recommendations of ATCC. To ensure the authenticity of all cell lines used in this study, Short Tandem Repeat analysis was performed with the PowerPlex 21 system.

### Plasmids and siRNA

Plasmid transfection was carried out using Lipofectamine 2000(1662298, Invitrogen). The plasmids HA-Ub, HA-K48/K48R, HA-K63/K63R originated from our earlier studies. The Myc-YAP, truncated mutants of YAP (amino acid 1–171, 1–292, 172–504, 293–504), lysine mutants of YAP (K76R, K90R, K97R, K102R, K181R, K204R, K252R, K254R, K280R, K315R, K321R, K342R, K494R, and K497R), and TEAD reporter gene plasmid were all generously provided by Zhou XF [40]. Flag-USP8 and mutant USP8 (amino acid 1–313, 314–714, 715–1118) plasmids were obtained from MiaoLingBio, China. We used small interfering RNA (siRNA) to silence specific genes, employing Lipofectamine RNA iMAX (13778150, Invitrogen) for siRNA transfection. The shRNA targeting USP8 (shUSP8) was subcloned into the pLKO.1 vector.

### Reagents

The reagents used were DUB-IN-2 (MCE, HY-50737A), MG132 (MCE, HY-15695), Cycloheximide (C7698, Sigma), and Verteporfin (MCE, HY-B0146).

### Screening of the DUB siRNA library

The human deubiquitinating enzyme (ON-TARGET plus) used in this study were sourced from the Dharmacon siRNA library (GU-104705). BT549 cells were transfected with various siDUBs for 48 h. RNA was then extracted and reverse transcribed into cDNA. The expression levels of the YAP canonical downstream gene CTGF by RT-qPCR. Meanwhile, after transfection with siDUBs for 48 h, BT549 cells were subjected to a dual-luciferase reporter assay to examine TEAD transcriptional activity, thereby evaluating the potential regulatory effects of DUBs on the Hippo signaling pathway.

### RNA extraction and real-time quantitative PCR

RNA isolater Total RNA Extraction Reagent (Vazyme, R401-01) was used to extract total RNA, and cDNA synthesis was performed with HiScript II Q RT SuperMix (Vazyme, R223-01). Real-time quantitative PCR was conducted on a 7500 real-time fluorescence quantitative PCR system (Applied Biosystems, Singapore) using gene-specific primers.

### Western blotting

After collecting the cells, cells were lysed using RIPA buffer (Beyotime, P0013). The protein samples were separated using SDS-PAGE and then transferred onto a PVDF membrane, which was blocked with 5% skim milk at room temperature for an hour. The membrane was washed thrice with PBST and then incubated with the appropriate primary antibody at 4 °C overnight. The membrane was treated with the secondary antibody for 1 hour following three washes with PBST. The blot was visualized using an ECL detection kit (MeilunBio, MA0186-1). The following is the information about antibodies used in immunoblot: anti-USP8 rabbit mAb (8782S), anti-YAP rabbit mAb (14074S) and anti-Flag mouse mAb (14793S) were purchased from Cell Signaling Technology and diluted 1:2000. Anti-β-actin rabbit mAb (20536-1-AP) were purchased from Proteintech and diluted 1:5000. Anti-YAP mouse mAb (sc-101199) was purchased from Santa Cruz Biotechnology and diluted 1:1000. Myc-tag rabbit mAb (AE070) was purchased from ABclonal and diluted 1:1000. Anti-Flag mouse mAb (F1804-200UG) was purchased from Sigma and diluted 1:2000. Anti-HA mouse mAb (90513) was purchased from Biolegend.

### Luciferase assay

BT549 or MDA-MB-231 cells were seeded into 48-well plates. Following transfection with siUSP8, BT549 or MDA-MB-231 cells were co-transfected with luciferase reporter and Renilla plasmids using Lipofectamine 2000. After a 24-h incubation period, cells were harvested and lysed for the subsequent luciferase assay using the Dual-Luciferase® Reporter Assay System (E1090, Promega).

### CCK8 assay

For cell viability assays, BT549 or MDA-MB-231 cells were transfected with siControl or siUSP8 in 12-well plates. After 24 h, 4000 cells were collected and re-seeded in triplicate into 96-well plates. For drug sensitivity analysis, 5000 cells were seeded in 96-well plates and incubated overnight, followed by treatment with specified concentrations of the drug. The CCK8 assay kit (TargetMol, C0005) was used to evaluate cell viability at various time points, and the absorbance of the cells was measured at a wavelength of 450 nm.

### EdU assay

The EdU assay kit (RIBOBIO, C10310-1) was used to evaluate cell proliferation. BT549 and MDA-MB-231 cells were seeded into 96-well plates following the respective treatments and incubated overnight. The cells were treated with 50 µM EdU for 2 h and then fixed with 4% paraformaldehyde. The staining process followed the manufacturer’s guidelines, using Hoechst 33342 to stain nucleic acids. Cell proliferation rates were determined with ImageJ.

### Wound healing assay

Place BT549 or MDA-MB-231 cells in a 6-well plate and process as needed until cells are fully integrated. With a 200 µl yellow pipette tip, create a straight line on cell monolayer. At the specified time intervals, measure the wound area and compare it to the initial size on the first day.

### Transwell assays

In the cell migration assay, 50,000 cells were placed in a serum-free medium and introduced into the upper chamber without a matrix. In the cell invasion assay, 50,000 cells were placed in a serum-free medium and introduced into the upper chamber lined with Matrigel. The lower chamber was filled with medium containing 20% fetal bovine serum. Cells in the lower chamber were fixed with methanol and stained with 0.2% crystal violet following a 12–14 h incubation.

### Flow cytometric analyses

In experiments on apoptosis, the FITC Annexin V Apoptosis Detection Kit (BD Pharmingen, 556547) was used to stain BT549 and MDA-MB-231 cells with Annexin V and PI. Following transfection with siUSP8, cells were collected and subjected to enzymatic dissociation using EDTA-free trypsin (03-045-1B, BI) to achieve a single-cell suspension. The cells were then washed three times with PBS and stained with the Annexin V-PI staining kit to detect apoptotic cells. A FACScan (Millipore) was used to measure fluorescence intensity, which was then analyzed using FlowJo 7.6 software.

### Co-immunoprecipitation assay

Cell proteins were collected and lysed on ice for 30 min using an IP lysis buffer supplemented with protease inhibitors. The supernatant, obtained after centrifugation at 12,000 × *g* for 15 min at 4 °C, was incubated overnight at 4 °C with the appropriate antibody or control IgG. The samples were mixed with protein A/G agarose and left to incubate for 2 h at 4 °C. The beads underwent three PBS washes, followed by a 10-min boil at 100 °C to reverse crosslinking, prior to SDS-PAGE immunoblotting analysis.

### Molecular docking analysis

Utilize GRAMM-X (http://gramm.compbio.ku.edu/) for rigid protein docking of USP8 and YAP to investigate their interaction. The protein domains of USP8 and YAP are sourced from the Protein Data Bank (PDB) database (http://www.rcsb.org/). Use PyMOL (Version 2.4) along with PDBePISA (https://www.ebi.ac.uk/pdbe/pisa/) to analyze protein–protein interactions and perform additional visualization studies.

### Poly-ubiquitination detection assay

For instance. To illustrate K48-linked polyubiquitination, 293T cells were co-transfected with Flag/Flag-USP8, HA-K48-Ub and Myc-YAP plasmids for 24 h, followed by treatment with 10 μM MG132 for 6 h before protein extraction. Subsequently, protein extracts were precleared with 40 μL of protein A/G for 3 h. The proteins were left with the anti-Myc antibody for the duration of the night, then incubated with protein A/G beads at 4 °C for an hour. Finally, anti-HA antibody was used in immunoblotting to identify the levels of K48 polyubiquitinated YAP.

### Protein stability assay

Cells were transfected with siControl/siUSP8 or Flag/Flag-USP8 wild-type/Flag-USP8 mutant plasmids. Following CHX treatment for the given time periods, cells were harvested and subjected to immunoblotting analysis using the particular antibodies. The quantification of YAP protein density is conducted using ImageJ software.

### Immunofluorescence assay

BT549 and MDA-MB-231 cells were fixed for 10 min using 4% paraformaldehyde (Beyotime, P0099). Permeabilized with 0.25% Triton X-100 (Solarbio, T8200) at room temperature for 5 min. Subsequently, it was blocked with 5% bovine serum albumin (BSA, Beyotime, ST025) at room temperature for one hour. Rabbit anti-USP8 antibody and mouse anti-YAP antibodies were used overnight at 4 °C, followed by Goat Anti-Rabbit IgG (AF488) and Goat Anti-Mouse IgG (AF594) for 1 h at room temperature in dark. Cells were washed three times with PBS and then stained with DAPI (Beyotime, C1006) to make the nucleus visible. A confocal laser scanning microscope was used to capture images, and ImageJ was utilized for further analysis.

### Immunohistochemistry

Tissue samples were treated with 4% paraformaldehyde, set in paraffin, and then sectioned into thin slices with a semi-automatic microtome. Immunohistochemistry (IHC) staining was conducted with an IHC kit (ORIGENE, PV-9000). Primary antibodies against USP8 (Cell Signaling Technology, 8782S), YAP (Cell Signaling Technology, 14074S) and Ki67 (Origene, ZM-0166) were utilized. After treating with primary and secondary antibodies, DAB was used to reveal immune complexes, and hematoxylin was applied to stain the cell nuclei.

### Xenograft mouse models

In the in vivo tumorigenesis test, 4 million cancer cells were mixed in 200 microliters of PBS and injected under the skin into the mammary fat pads of 4-week-old female BALB/c mice. The tumorigenic potential was evaluated by observing mice for tumor development over a span of about 5 weeks. In the drug treatment study, once the average tumor volume reached 100 mm^3^, mice received intraperitoneal injections of DMSO, DUB-IN-2, or PTX (10 mg/kg in DMSO) every 3 days, totaling 6 injections. The formula for calculating tumor volume is as follows:(length × width^2^)/2. All mouse experiments were conducted in accordance with the guidelines approved by the Animal Care Commission of Xinxiang Medical University.

### Chromatin immunoprecipitation (ChIP) assay

ChIP analysis was performed on BT549 and MDA-MB-231 cell lines. Cells were fixed for 30 min to facilitate crosslinking, followed by neutralization with 0.1375 mol/L glycine. After washing and scraping into cold PBS/1 mmol/L PMSF, the cells were centrifuged and treated with SDS lysis buffer. Chromatin was sonicated for 10 min with 30-s intervals. Subsequent detection steps utilized a ChIP assay kit (Millipore, 17–295). In this ChIP experiment, a rabbit polyclonal antibody against YAP (Cell Signaling Technology, 14074S) was utilized. DNA extraction was performed using a Qiagen DNA extraction kit (Qiagen, Cat. no. 28106).

### Publicly available clinical data analysis

The genomic data commons (GDC) data portal (https://portal.gdc.cancer.gov/) provides USP8 RNA-seq data for tumors of triple-negative breast cancer. Prism 8.0 (GraphPad) was used to analyze and compute the collected data. Gene sets for CORDENONSI YAP CONSERVED SIGNATURE were utilized and obtained from the GSEA Molecular Signatures Database. Heatmap plot was performed using the Xiantao online (https://www.xiantao.love/). The KMPLOT database was used to examine the link between USP8 expression and clinical outcomes.

### RNA-seq and analysis

We employed GSEA to evaluate the enrichment of YAP-positive regulatory genes between the siControl and siUSP8 groups. We performed enrichment analysis using Metascape’s Hallmark gene sets to investigate the pathways linked to differentially expressed genes (DEGs). In addition, a volcano plot for DEGs was created using the OmicStudio tool, applying a threshold of *P* < 0.05 and a fold change > 1.5.

### Statistics

In this study, publicly available data were analyzed using statistical tools like Student’s *t* tests and Pearson correlation coefficients. The data are shown as the average ± standard deviation (SD), with statistical significance indicated by *P* < 0.05 (*), *P* < 0.01 (**), and *P* < 0.001 (***).

## Supplementary information

Supplementary Figure 1

Supplementary Figure 2

Supplementary Figure 3

Supplementary Figure legends

Original Western Blot

Original Data

Supplementary Materials

## Data Availability

The supplementary materials contain the original siRNA screening data, as well as the raw data for Western blot and qRT-PCR. The supplementary materials provide information on cell line authentication and primer sequences.

## References

[1] Foulkes WD, Smith IE, Reis-Filho JS. Triple-negative breast cancer. N Engl J Med. 2010;363:1938–48. 21067385 10.1056/NEJMra1001389

[2] Plasilova ML, Hayse B, Killelea BK, Horowitz NR, Chagpar AB, Lannin DR. Features of triple-negative breast cancer: analysis of 38,813 cases from the national cancer database. Medicine. 2016;95:e4614. 27583878 10.1097/MD.0000000000004614 PMC5008562

[3] Irvin JrWJ, Carey LA. What is triple-negative breast cancer? Eur J Cancer. 2008;44:2799–805. 19008097 10.1016/j.ejca.2008.09.034

[4] Jamdade VS, Sethi N, Mundhe NA, Kumar P, Lahkar M, Sinha N. Therapeutic targets of triple-negative breast cancer: a review. Br J Pharmacol. 2015;172:4228–37. 26040571 10.1111/bph.13211 PMC4556464

[5] Zhang J, Yao S, Hu Q, Zhu Q, Liu S, Lunetta KL, et al. Genetic variations in the Hippo signaling pathway and breast cancer risk in African American women in the AMBER consortium. Carcinogenesis. 2016;37:951–6. 27485598 10.1093/carcin/bgw077 PMC5035397

[6] Bossuyt W, Chen C-L, Chen Q, Sudol M, McNeill H, Pan D, et al. An evolutionary shift in the regulation of the Hippo pathway between mice and flies. Oncogene. 2014;33:1218–28. 23563179 10.1038/onc.2013.82 PMC4613760

[7] Chibly AM, Aure MH, Patel VN, Hoffman MP. Salivary gland function, development, and regeneration. Physiol Rev. 2022;102:1495–552. 35343828 10.1152/physrev.00015.2021 PMC9126227

[8] Li S, Li Q, Zhu Y, Hu W. GDF15 induced by compressive force contributes to osteoclast differentiation in human periodontal ligament cells. Exp Cell Res. 2020;387:111745. 31765611 10.1016/j.yexcr.2019.111745

[9] Kodaka M, Hata Y. The mammalian Hippo pathway: regulation and function of YAP1 and TAZ. Cell Mol Life Sci. 2015;72:285–306. 25266986 10.1007/s00018-014-1742-9 PMC11113917

[10] Piccolo S, Dupont S, Cordenonsi M. The biology of YAP/TAZ: Hippo signaling and beyond. Physiol Rev. 2014;94:1287–312. 25287865 10.1152/physrev.00005.2014

[11] Piccolo FM, Kastan NR, Haremaki T, Tian Q, Laundos TL, De Santis R, et al. Role of YAP in early ectodermal specification and a Huntington’s disease model of human neurulation. Elife. 2022;11:e73075. 35451959 10.7554/eLife.73075 PMC9033270

[12] Maugeri-Saccà M, De Maria R. Hippo pathway and breast cancer stem cells. Crit Rev Oncol Hematol. 2016;99:115–22. 26725175 10.1016/j.critrevonc.2015.12.004

[13] min Kim H, Kim SK, Jung WH, Koo JS. Metaplastic carcinoma show different expression pattern of YAP compared to triple-negative breast cancer. Tumor Biol. 2015;36:1207–12. 10.1007/s13277-014-2735-x 25344213

[14] Song H, Wu T, Xie D, Li D, Hua K, Hu J, et al. WBP2 downregulation inhibits proliferation by blocking YAP transcription and the EGFR/PI3K/Akt signaling pathway in triple negative breast cancer. Cell Physiol Biochem. 2018;48:1968–82. 30092563 10.1159/000492520

[15] Casalino L, Talotta F, Cimmino A, Verde P. The Fra-1/AP-1 oncoprotein: from the “undruggable” transcription factor to therapeutic targeting. Cancers. 2022;14:1480. 35326630 10.3390/cancers14061480 PMC8946526

[16] Calvo F, Ege N, Grande-Garcia A, Hooper S, Jenkins RP, Chaudhry SI, et al. Mechanotransduction and YAP-dependent matrix remodelling is required for the generation and maintenance of cancer-associated fibroblasts. Nat Cell Biol. 2013;15:637–46. 23708000 10.1038/ncb2756 PMC3836234

[17] Luo J, Zou H, Guo Y, Tong T, Chen Y, Xiao Y, et al. The oncogenic roles and clinical implications of YAP/TAZ in breast cancer. Br J Cancer. 2023;128:1611–24. 36759723 10.1038/s41416-023-02182-5 PMC10133323

[18] Zhang Y, Fan Y, Jing X, Zhao L, Liu T, Wang L, et al. OTUD5-mediated deubiquitination of YAP in macrophage promotes M2 phenotype polarization and favors triple-negative breast cancer progression. Cancer Lett. 2021;504:104–15. 33587979 10.1016/j.canlet.2021.02.003

[19] Zou X, Levy-Cohen G, Blank M. Molecular functions of NEDD4 E3 ubiquitin ligases in cancer. Biochim Biophys Acta Rev Cancer. 2015;1856:91–106. 10.1016/j.bbcan.2015.06.005 26116757

[20] Harrigan JA, Jacq X, Martin NM, Jackson SP. Deubiquitylating enzymes and drug discovery: emerging opportunities. Nat Rev Drug Discov. 2018;17:57–78. 28959952 10.1038/nrd.2017.152 PMC7097658

[21] Lange SM, Armstrong LA, Kulathu Y. Deubiquitinases: from mechanisms to their inhibition by small molecules. Mol Cell. 2022;82:15–29. 34813758 10.1016/j.molcel.2021.10.027

[22] Xiong W, Gao X, Zhang T, Jiang B, Hu M-M, Bu X, et al. USP8 inhibition reshapes an inflamed tumor microenvironment that potentiates the immunotherapy. Nat Commun. 2022;13:1700. 35361799 10.1038/s41467-022-29401-6 PMC8971425

[23] Tang J, Long G, Hu K, Xiao D, Liu S, Xiao L, et al. Targeting USP8 inhibits O-GlcNAcylation of SLC7A11 to promote ferroptosis of hepatocellular carcinoma via stabilization of OGT. Adv Sci. 2023;10:2302953. 10.1002/advs.202302953 PMC10667802 37867237

[24] Zhu C, Li L, Zhang Z, Bi M, Wang H, Su W, et al. A non-canonical role of YAP/TEAD is required for activation of estrogen-regulated enhancers in breast cancer. Mol Cell. 2019;75:791–806. 31303470 10.1016/j.molcel.2019.06.010 PMC6707877

[25] Bianchini G, De Angelis C, Licata L, Gianni L. Treatment landscape of triple-negative breast cancer—expanded options, evolving needs. Nat Rev Clin Oncol. 2022;19:91–113. 34754128 10.1038/s41571-021-00565-2

[26] Liao L, Zhang Y-L, Deng L, Chen C, Ma X-Y, Andriani L, et al. Protein phosphatase 1 subunit PPP1R14B stabilizes STMN1 to promote progression and paclitaxel resistance in triple-negative breast cancer. Cancer Res. 2023;83:471–84. 36484700 10.1158/0008-5472.CAN-22-2709 PMC9896024

[27] Nedeljković M, Damjanović A. Mechanisms of chemotherapy resistance in triple-negative breast cancer—how we can rise to the challenge. Cells. 2019;8:957. 31443516 10.3390/cells8090957 PMC6770896

[28] Daviet L, Colland F. Targeting ubiquitin specific proteases for drug discovery. Biochimie. 2008;90:270–83. 17961905 10.1016/j.biochi.2007.09.013

[29] Xie F, Zhou X, Li H, Su P, Liu S, Li R, et al. USP8 promotes cancer progression and extracellular vesicle-mediated CD8+ T cell exhaustion by deubiquitinating the TGF-β receptor TβRII. EMBO J. 2022;41:e108791. 35811497 10.15252/embj.2021108791 PMC9379553

[30] Durcan TM, Fon EA. USP8 and PARK2/parkin-mediated mitophagy. Autophagy. 2015;11:428–9. 25700639 10.1080/15548627.2015.1009794 PMC4502724

[31] Durcan TM, Tang MY, Pérusse JR, Dashti EA, Aguileta MA, McLelland GL, et al. USP 8 regulates mitophagy by removing K 6-linked ubiquitin conjugates from parkin. EMBO J. 2014;33:2473–91. 25216678 10.15252/embj.201489729 PMC4283406

[32] Jiao D, Chen Y, Liu X, Tang X, Chen J, Liu Y, et al. Targeting MET endocytosis or degradation to overcome HGF-induced gefitinib resistance in EGFR-sensitive mutant lung adenocarcinoma. Biochem Biophys Res Commun. 2023;682:371–80. 37844446 10.1016/j.bbrc.2023.10.037

[33] Zhu Y, Xu J, Hu W, Wang F, Zhou Y, Gong W, et al. Inhibiting USP8 overcomes hepatocellular carcinoma resistance via suppressing receptor tyrosine kinases. Aging. 2021;13:14999. 34081623 10.18632/aging.203061 PMC8221339

[34] Chapeau EA, Sansregret L, Galli GG, Chène P, Wartmann M, Mourikis TP, et al. Direct and selective pharmacological disruption of the YAP-TEAD interface by IAG933 inhibits Hippo-dependent and RAS-MAPK-altered cancers. Nat Cancer. 2024;5:1102–20. 38565920 10.1038/s43018-024-00754-9 PMC11286534

[35] Pobbati AV, Hong W. A combat with the YAP/TAZ-TEAD oncoproteins for cancer therapy. Theranostics. 2020;10:3622. 32206112 10.7150/thno.40889 PMC7069086

[36] Zeng R, Dong J. The Hippo signaling pathway in drug resistance in cancer. Cancers. 2021;13:318. 33467099 10.3390/cancers13020318 PMC7830227

[37] Dey A, Varelas X, Guan K-L. Targeting the Hippo pathway in cancer, fibrosis, wound healing and regenerative medicine. Nat Rev Drug Discov. 2020;19:480–94. 32555376 10.1038/s41573-020-0070-z PMC7880238

[38] Yang NJ, Hinner MJ. Getting across the cell membrane: an overview for small molecules, peptides, and proteins. Site-specific protein labeling. Methods and protocols. 2015;1266:29–53. 10.1007/978-1-4939-2272-7_3 PMC4891184 25560066

[39] Cho W, Stahelin RV. Membrane-protein interactions in cell signaling and membrane trafficking. Annu Rev Biophys Biomol Struct. 2005;34:119–51. 15869386 10.1146/annurev.biophys.33.110502.133337

[40] Zhou X, Li Y, Wang W, Wang S, Hou J, Zhang A, et al. Regulation of Hippo/YAP signaling and esophageal squamous carcinoma progression by an E3 ubiquitin ligase PARK2. Theranostics. 2020;10:9443. 32863938 10.7150/thno.46078 PMC7449928

