# Supplementary figures and images for: Positive feedback regulation between USP8 and Hippo/YAP axis drives triple-negative breast cancer progression
Source: Cell Death Dis. 2026 Jan 21;17(1):98. doi: 10.1038/s41419-025-08356-8 (PMC12830590; doi:10.1038/s41419-025-08356-8)

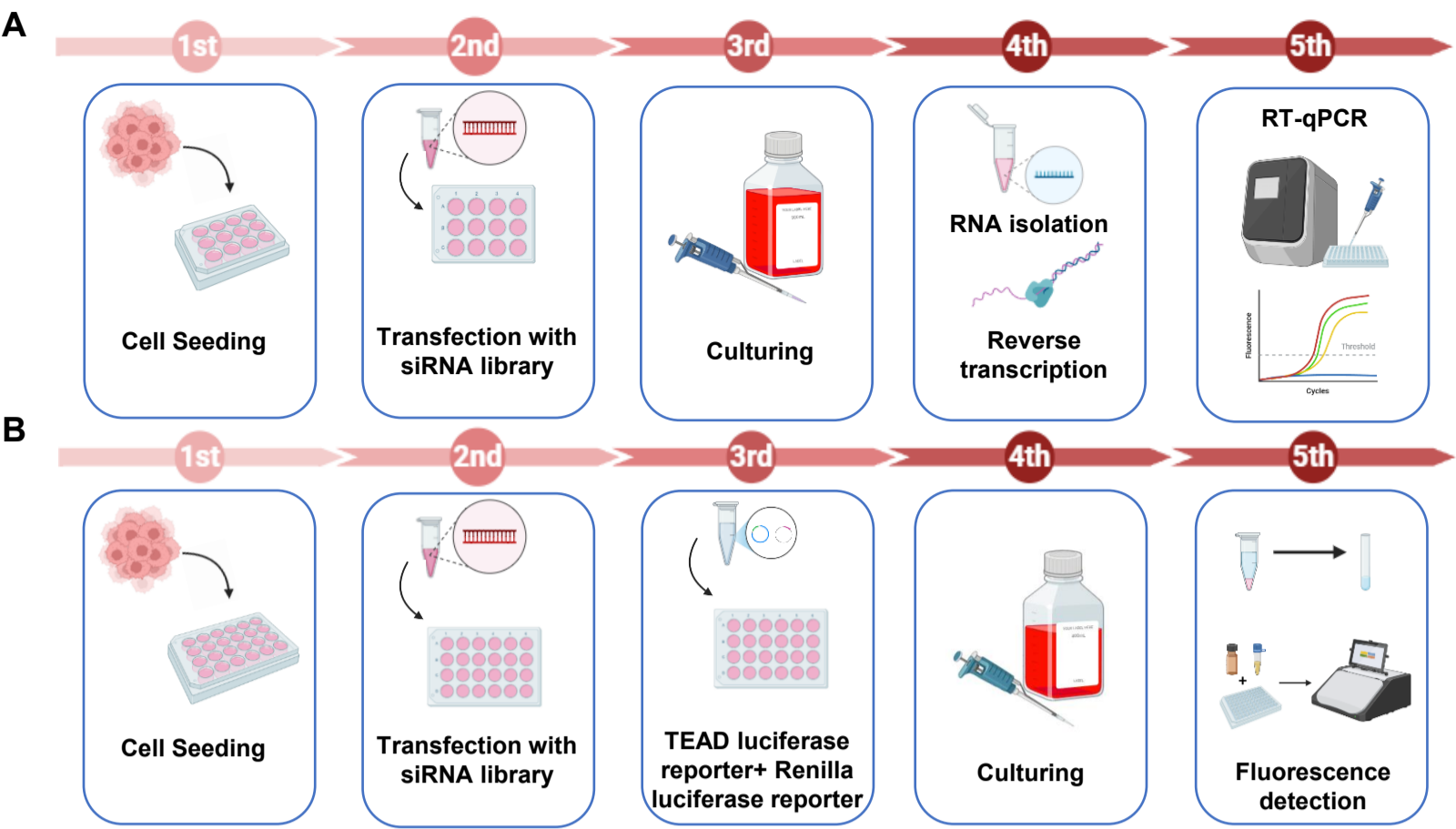

Supplement: Supplementary file 1 — Supplementary Figure 1 [file 41419_2025_8356_MOESM1_ESM.pdf]

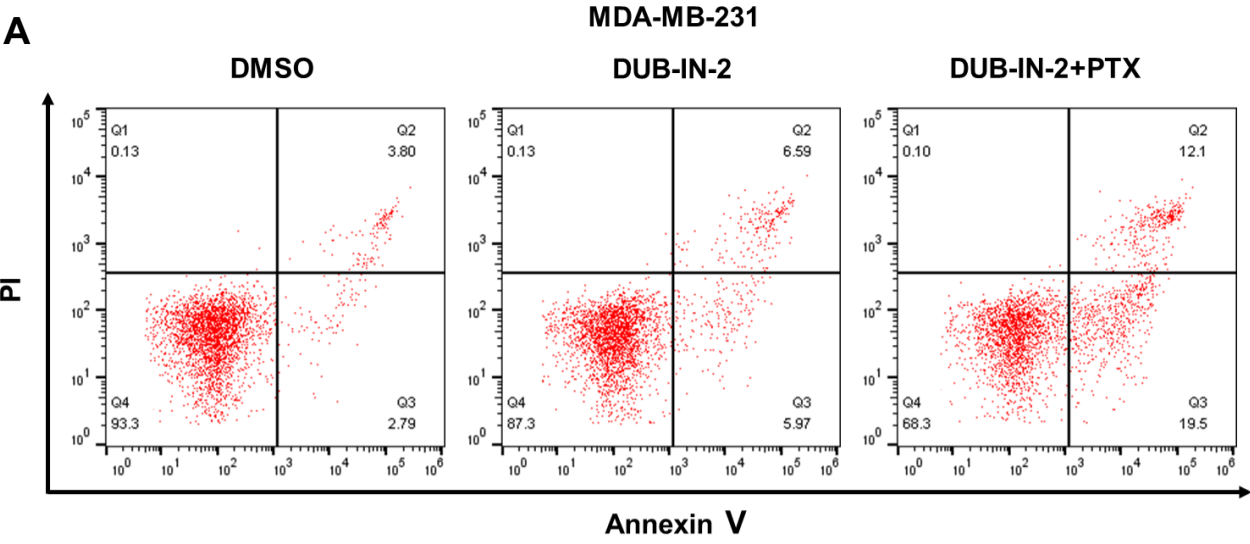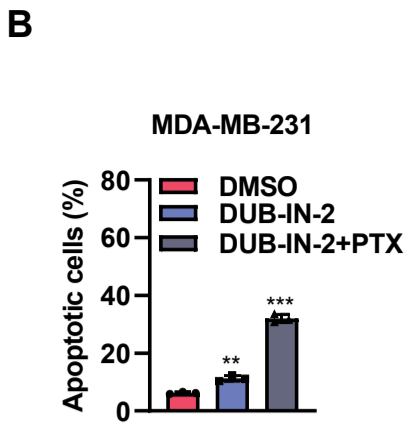

Supplement: Supplementary file 2 — Supplementary Figure 2 [file 41419_2025_8356_MOESM2_ESM.pdf]

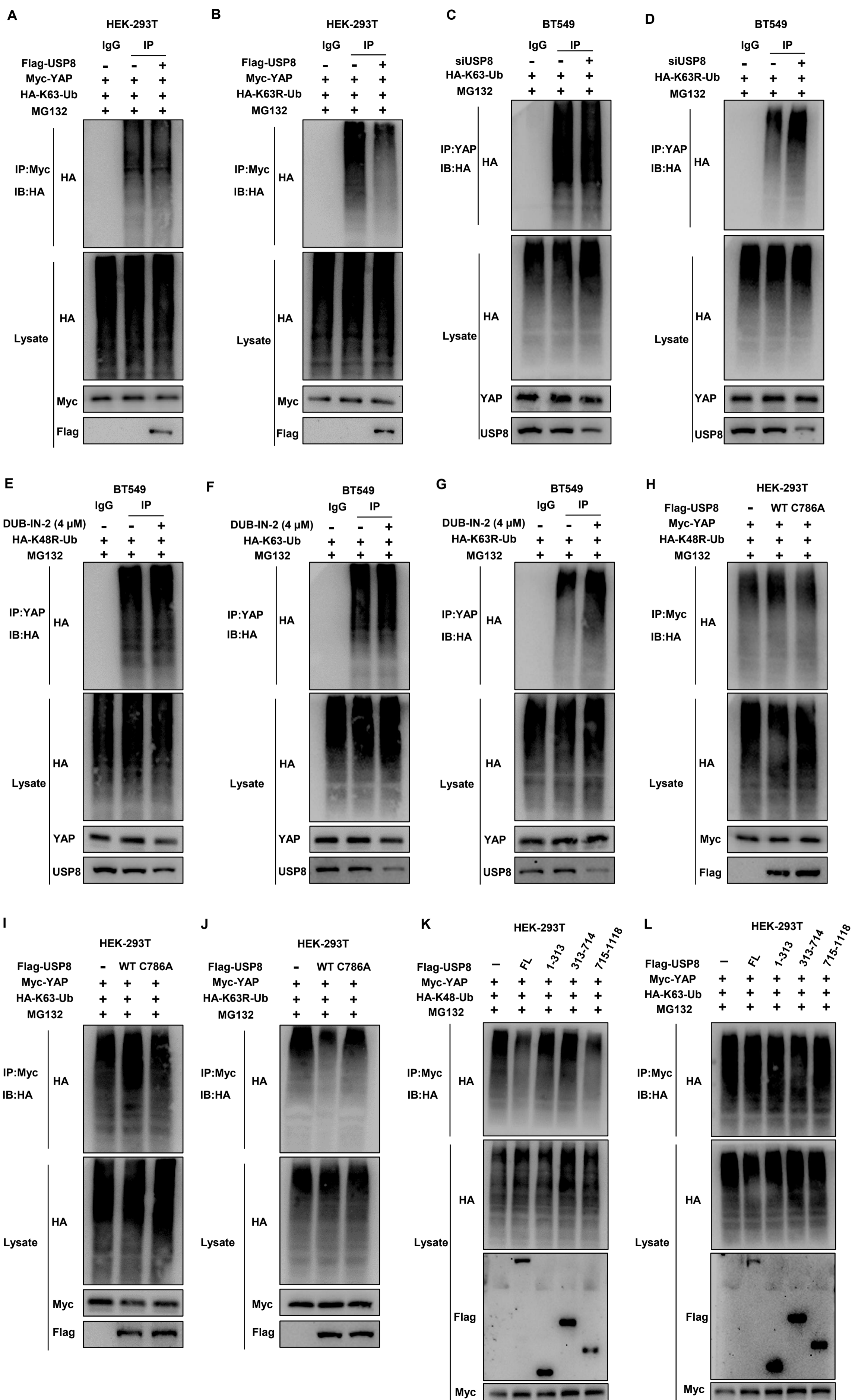

Supplement: Supplementary file 3 — Supplementary Figure 3 [file 41419_2025_8356_MOESM3_ESM.pdf]
