# Supplementary material for: Positive feedback regulation between USP8 and Hippo/YAP axis drives triple-negative breast cancer progression
Source: Cell Death Dis. 2026 Jan 21;17(1):98. doi: 10.1038/s41419-025-08356-8 (PMC12830590; doi:10.1038/s41419-025-08356-8)
Supplement: Supplementary file 4 — Supplementary Figure legends [file 41419_2025_8356_MOESM4_ESM.docx]

**Supplementary Figure Legends**

**Supplementary Figure 1. Deubiquitinating Enzyme Screening Flowchart**

A: This flowchart illustrates the screening procedure for essential deubiquitinating enzymes. Following the transfection of the deubiquitinating enzyme library into BT549 cells for 36 hours, gene expression levels were measured by real-time PCR.

B: The flowchart shows the screening process of key deubiquitinating enzymes. After the deubiquitination enzyme library was transfected into BT549 cells for 24 h, the TEAD luciferase reporter gene and the renilla luciferase reporter gene were transfected for 24 h, and the luciferase reporter gene assay was employed to measure the transcriptional activity of associated genes.

**Supplementary Figure 2. Combined treatment with PTX and USP8 inhibitors significantly increases the proportion of apoptotic cells.**

A: Flow cytometry was used to determine the apoptosis level of MDA-MB-231 cell lines treated with DMSO or DUB-IN-2 or DUB-IN-2+PTX for 12 hours.

B: The quantitative results of apoptosis.

**Supplementary Figure 3. USP8 stabilizes YAP by reducing K48 poly-ubiquitination**

A-B: The level of K63/K63R-specific ubiquitination of YAP was assessed through immunoblotting with relevant antibodies.

C-D: The level of K63/K63R-specific ubiquitination of YAP was assessed through immunoblotting using appropriate antibodies.

E-G: Immunoblotting was used to measure the degree of YAP's K48 and K63/K63R-specific ubiquitination in BT549 cells. The subsequent analysis was performed using specific antibodies.

H-J: The immunoblotting was utilized to ascertain the levels of YAP ubiquitination specific to K48R and K63/K63R.

K: Utilizing immunoblotting, the level of K48-specific ubiquitination on YAP was determined.

L: The level of K63-specific ubiquitination on YAP was established through immunoblotting.
