# Supplementary material for: Positive feedback regulation between USP8 and Hippo/YAP axis drives triple-negative breast cancer progression
Source: Cell Death Dis. 2026 Jan 21;17(1):98. doi: 10.1038/s41419-025-08356-8 (PMC12830590; doi:10.1038/s41419-025-08356-8)

Figure 2A

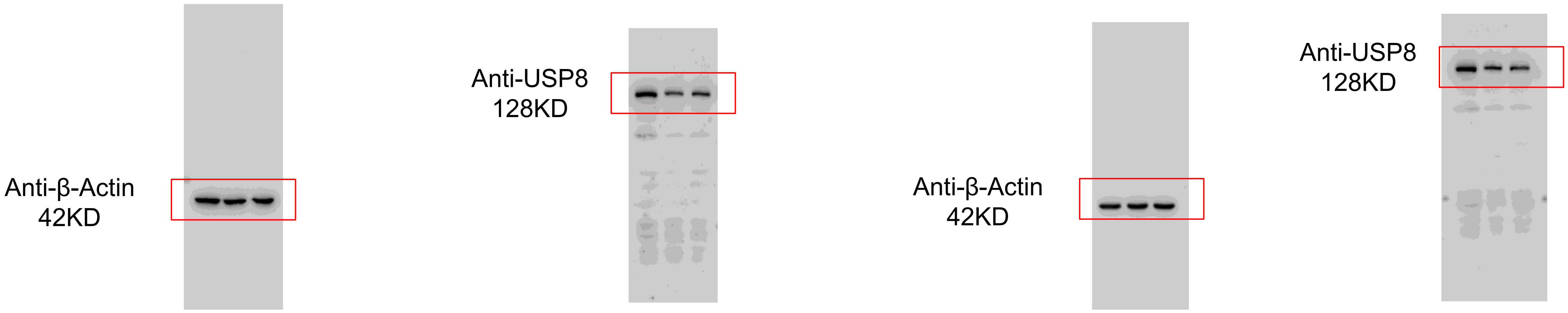
Anti-β-Actin
42KD
Anti-USP8
128KD
Anti-β-Actin
42KD
Anti-USP8
128KD

Figure 3A

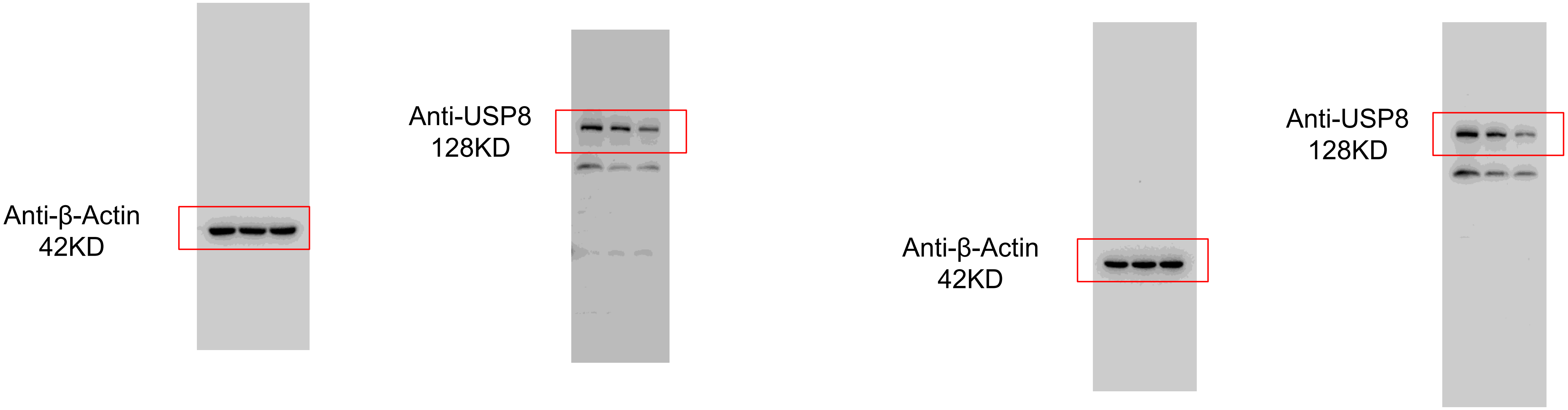
Anti-β-Actin
42KD
Anti-USP8
128KD
Anti-β-Actin
42KD
Anti-USP8
128KD

Figure 4A

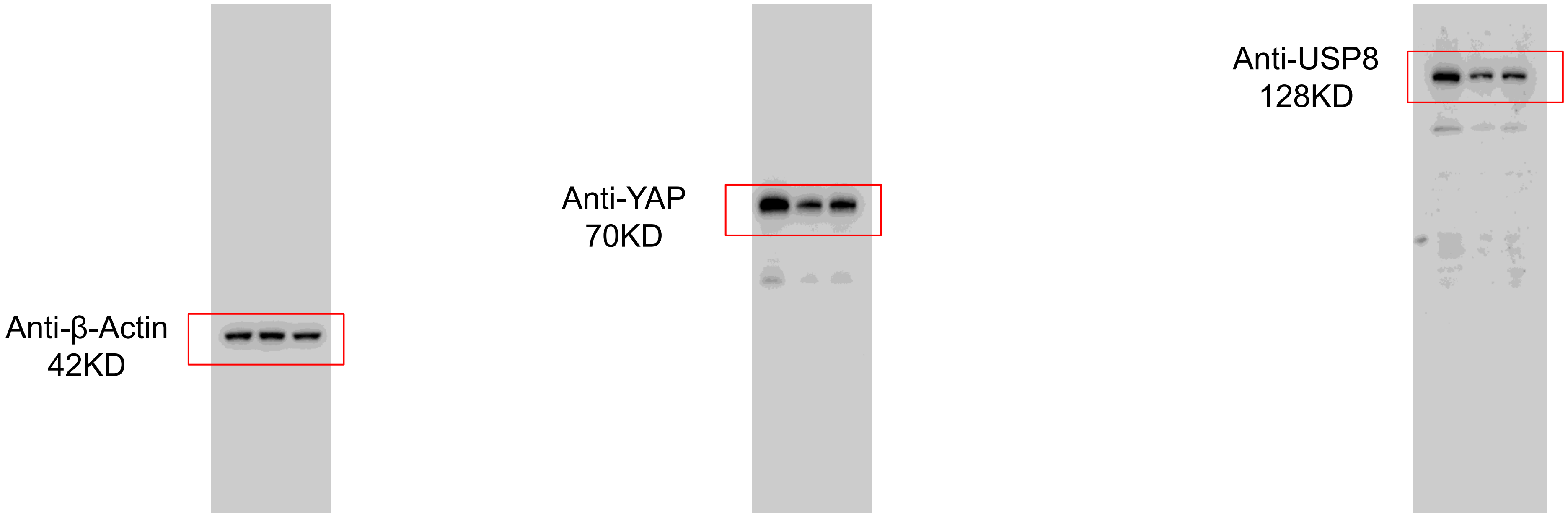

Figure 4B

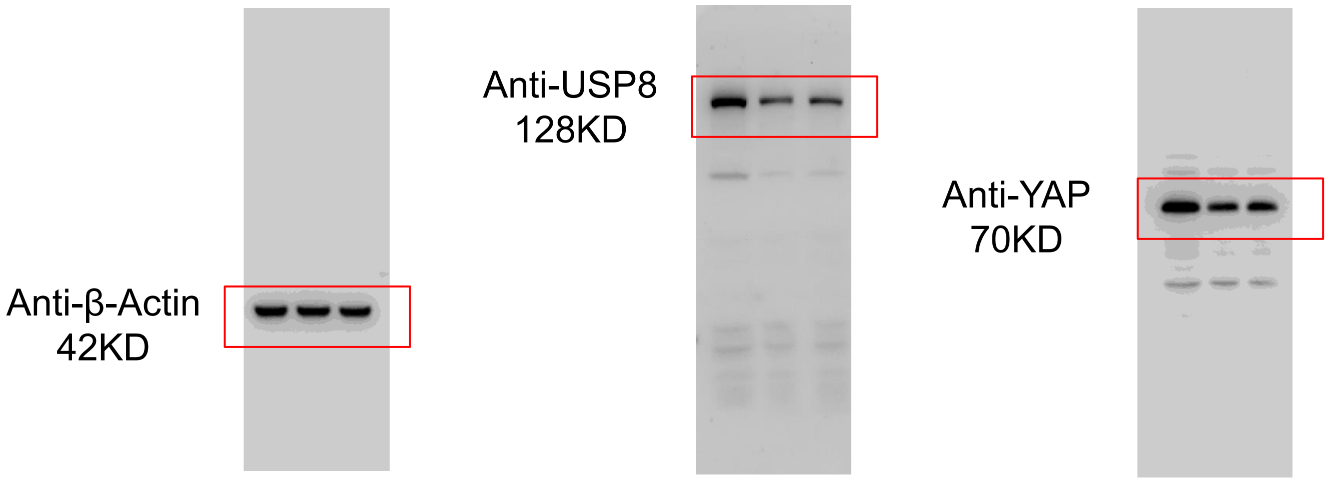

Figure 4I

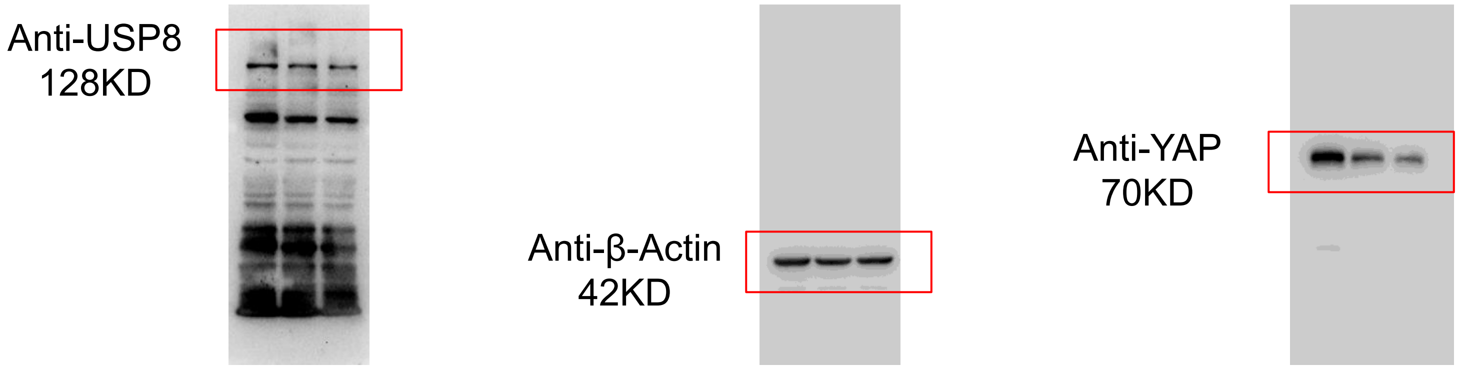

Figure 4J

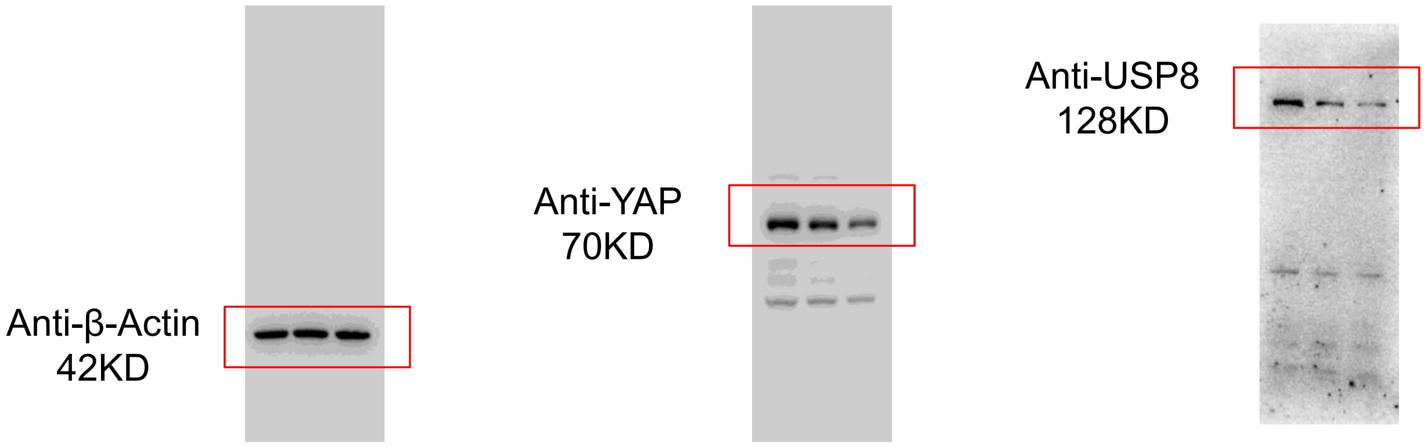

Figure 5A

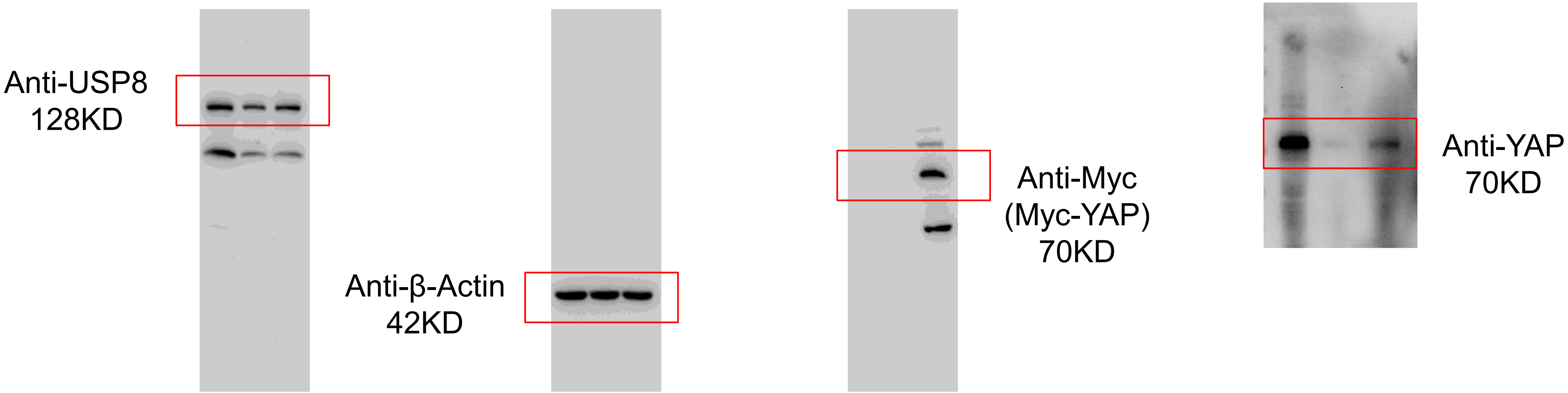
Anti-USP8
128KD
Anti-β-Actin
42KD
Anti-Myc
(Myc-YAP)
70KD
Anti-YAP
70KD

Figure 6B

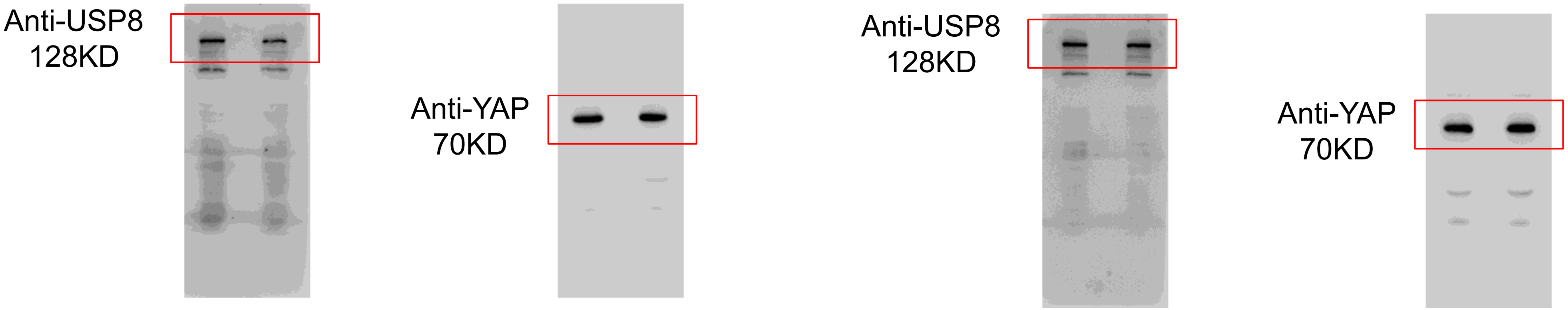

Figure 6E

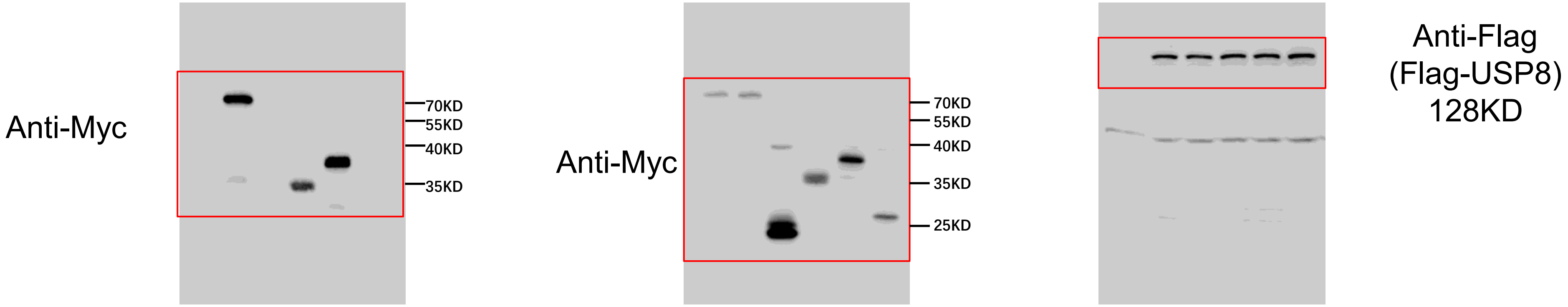
Anti-Myc
70KD
55KD
40KD
35KD
Anti-Myc
70KD
55KD
40KD
35KD
25KD
Anti-Flag
(Flag-USP8)
128KD

Figure 6F

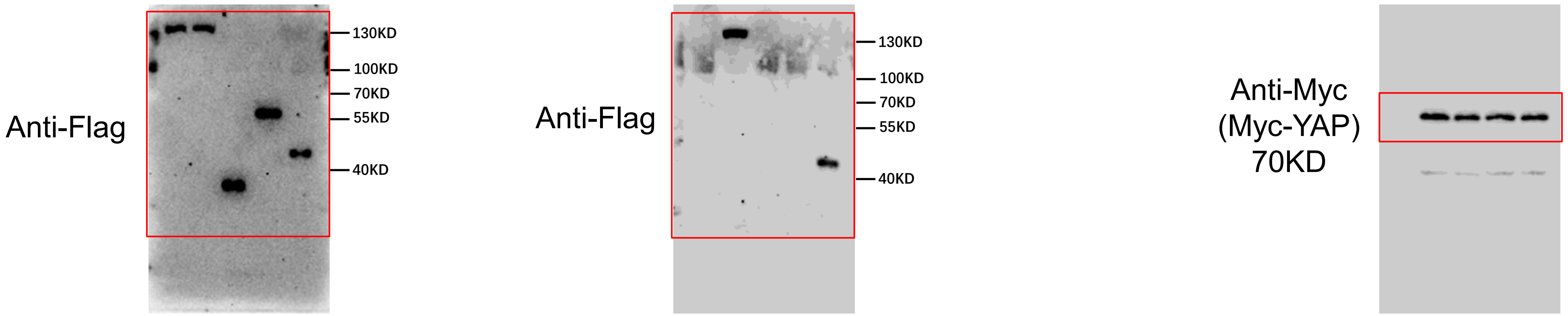
Anti-Flag
130KD
100KD
70KD
55KD
40KD
Anti-Flag
130KD
100KD
70KD
55KD
40KD
Anti-Myc
(Myc-YAP)
70KD

Figure 6I

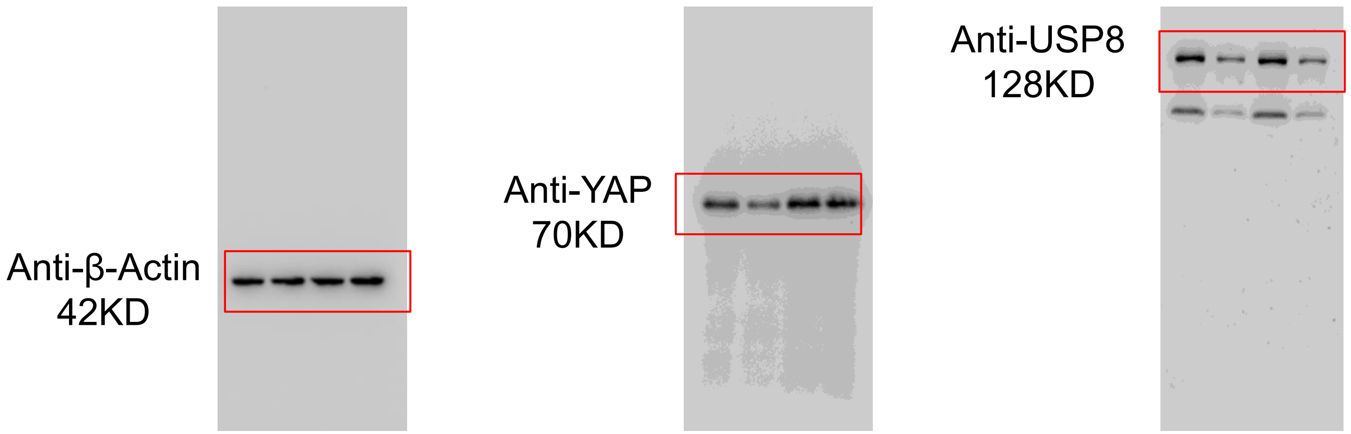

Figure 6J

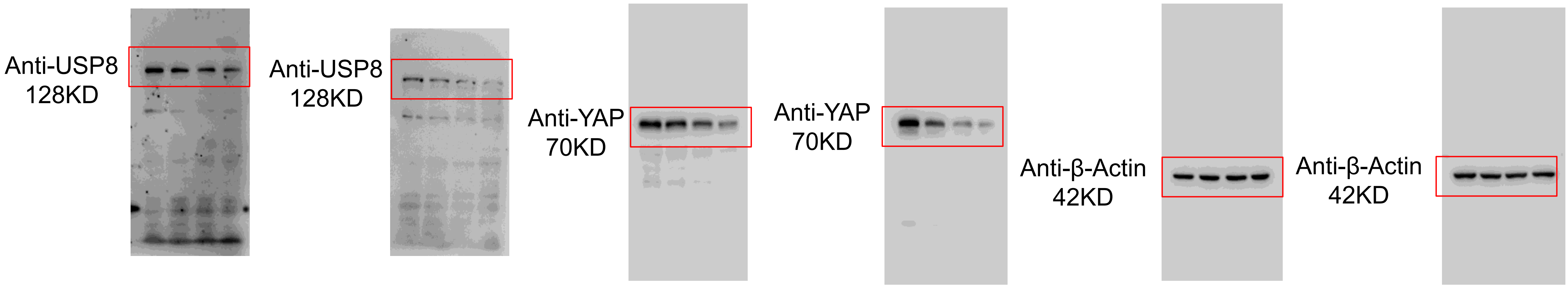

Figure 6L

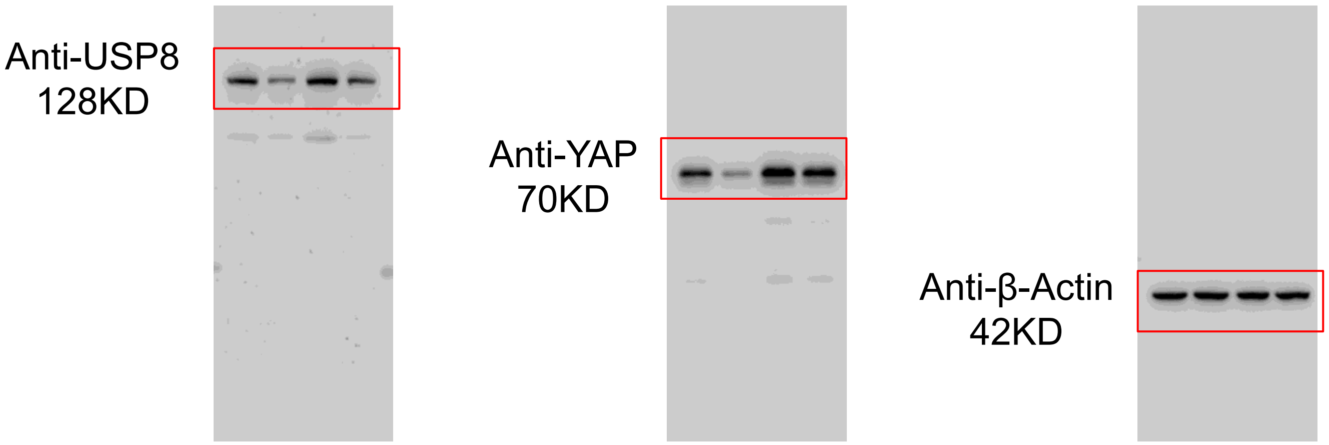

Figure 6M

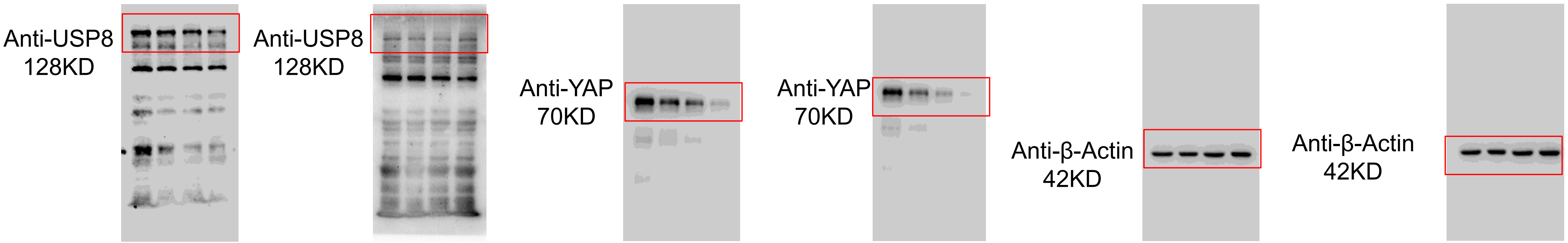

Figure 6O

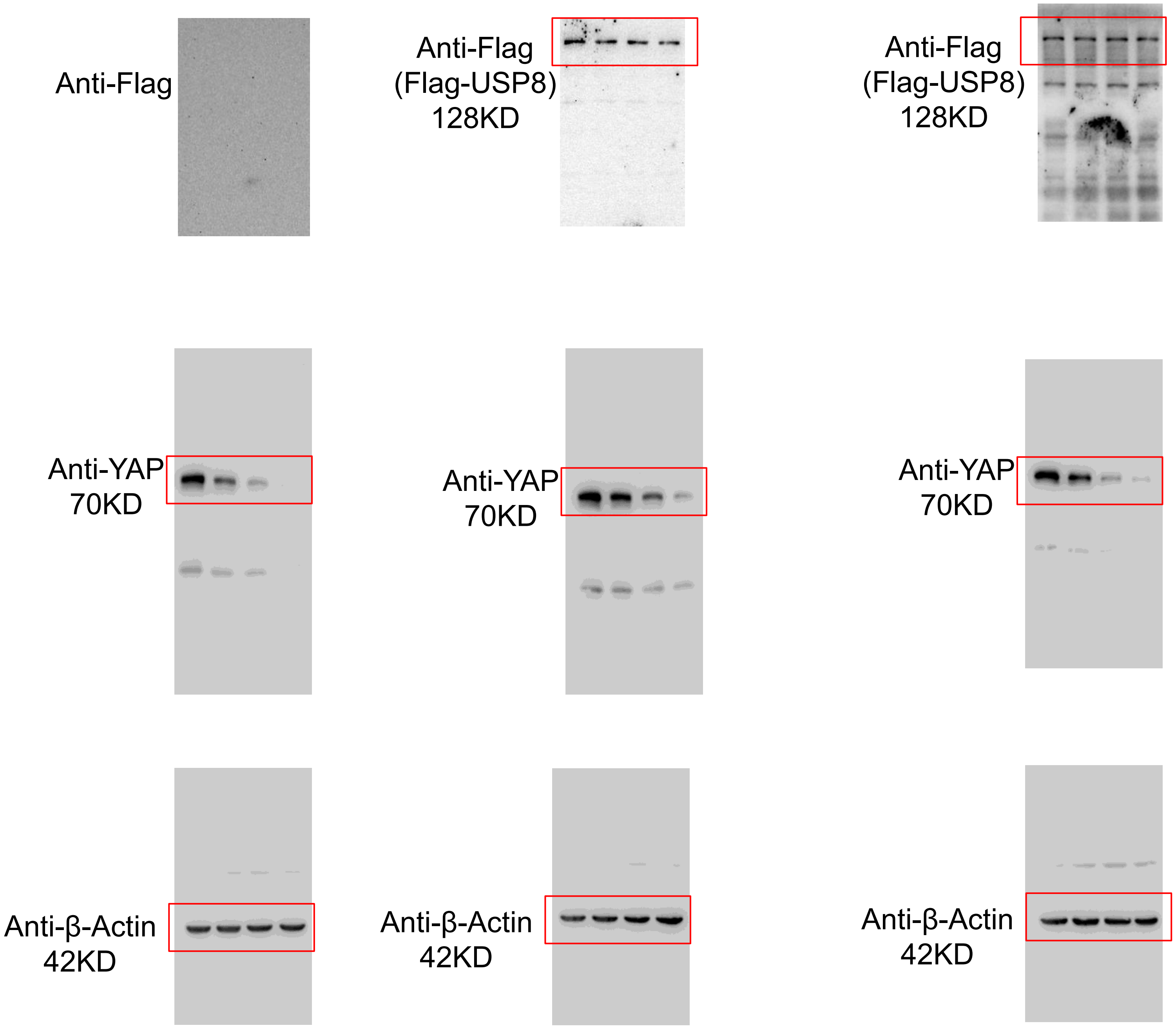

Anti-Flag
Anti-Flag
(Flag-USP8)
128KD
Anti-Flag
(Flag-USP8)
128KD
Anti-YAP
70KD
Anti-YAP
70KD
Anti-YAP
70KD
Anti-β-Actin
42KD
Anti-β-Actin
42KD
Anti-β-Actin
42KD

Figure 7A

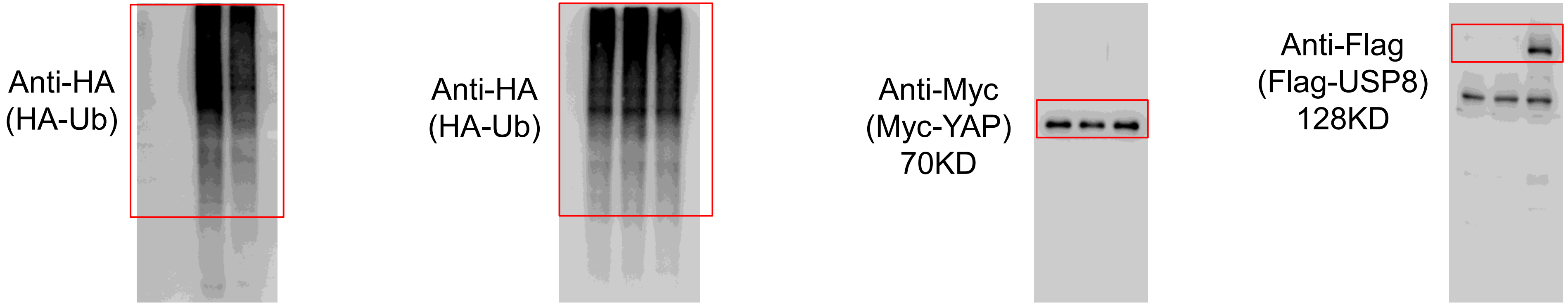

Anti-HA
(HA-Ub)
Anti-HA
(HA-Ub)
Anti-Myc
(Myc-YAP)
70KD
Anti-Flag
(Flag-USP8)
128KD

Figure 7B

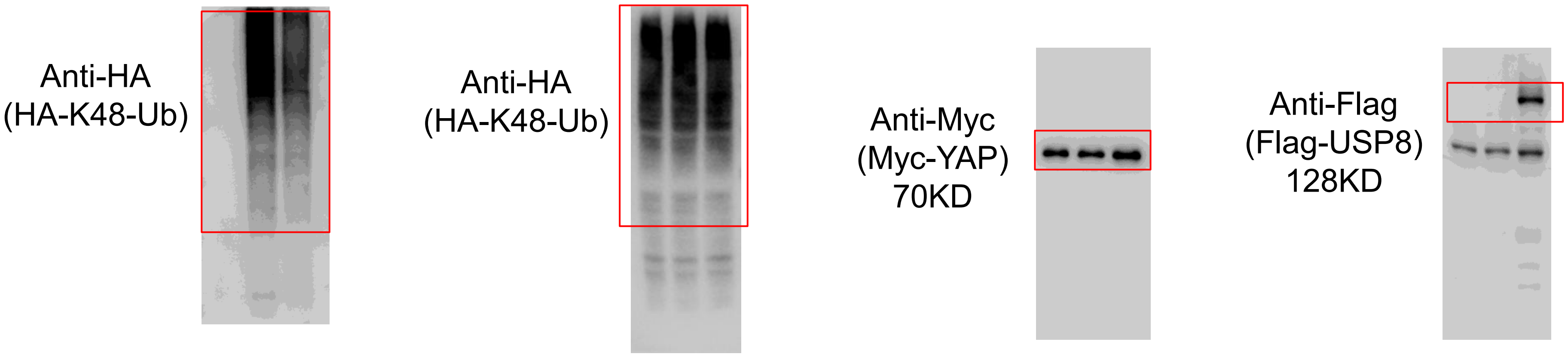

Anti-HA
(HA-K48-Ub)
Anti-HA
(HA-K48-Ub)
Anti-Myc
(Myc-YAP)
70KD
Anti-Flag
(Flag-USP8)
128KD

Figure 7C

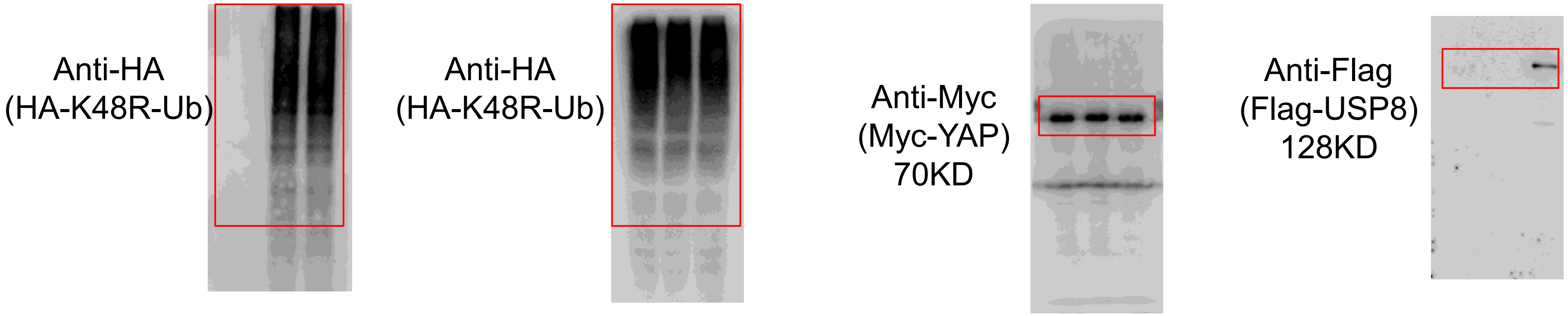
Anti-HA
(HA-K48R-Ub)
Anti-HA
(HA-K48R-Ub)
Anti-Myc
(Myc-YAP)
70KD
Anti-Flag
(Flag-USP8)
128KD

Figure 7D

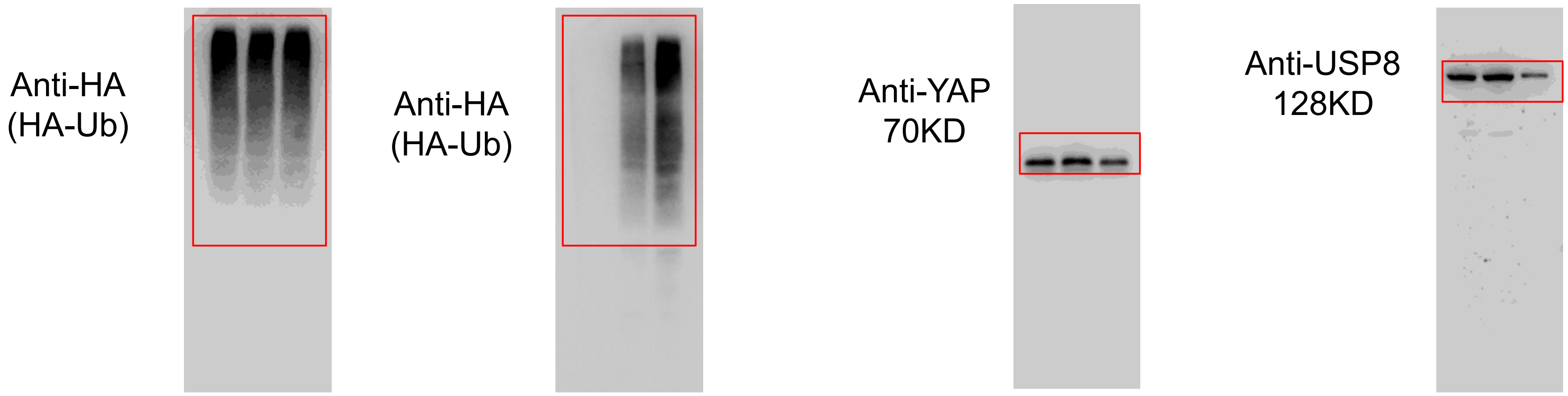

Figure 7E

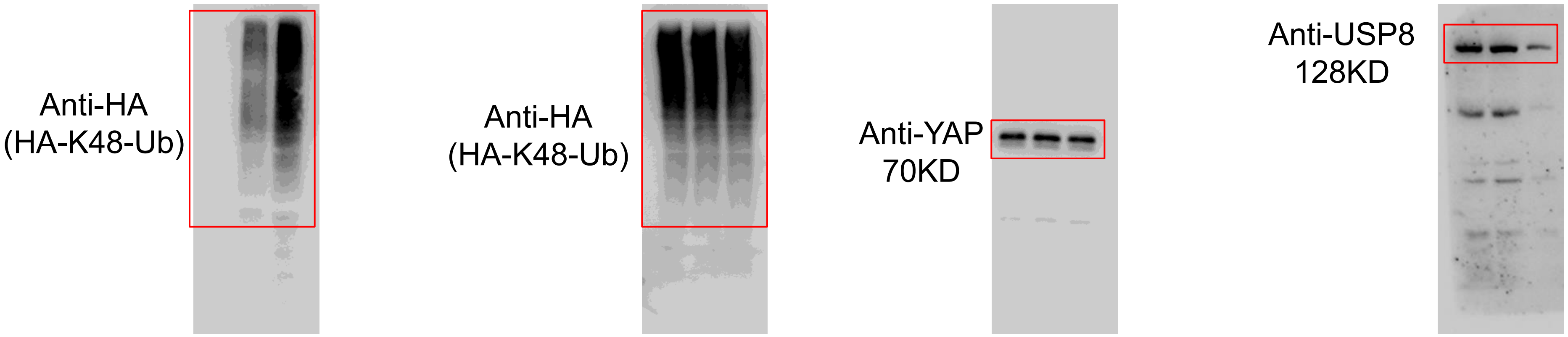
Anti-HA
(HA-K48-Ub)
Anti-HA
(HA-K48-Ub)
Anti-YAP
70KD
Anti-USP8
128KD

Figure 7F

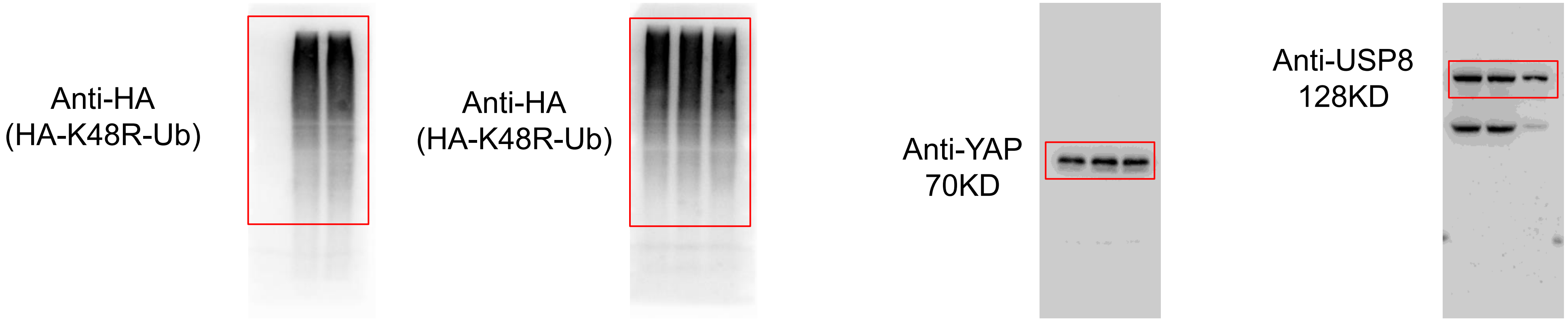
Anti-HA
(HA-K48R-Ub)
Anti-HA
(HA-K48R-Ub)
Anti-YAP
70KD
Anti-USP8
128KD

Figure 7G

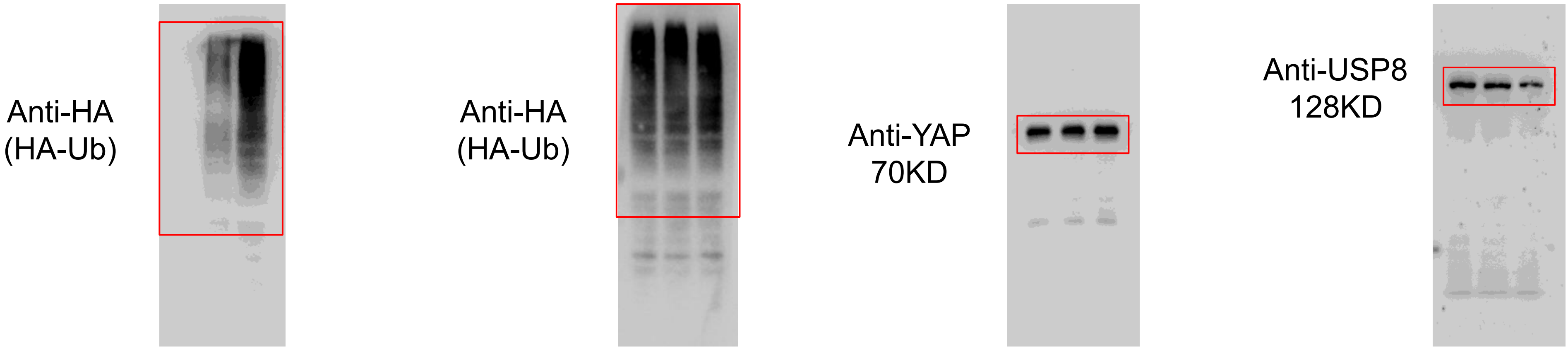

Figure 7H

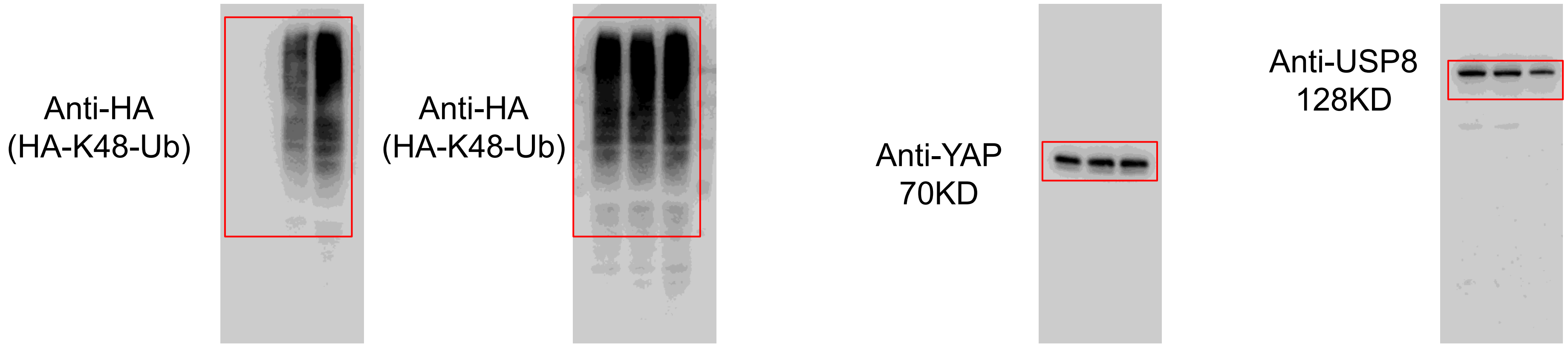
Anti-HA
(HA-K48-Ub)
Anti-HA
(HA-K48-Ub)
Anti-YAP
70KD
Anti-USP8
128KD

Figure 7I

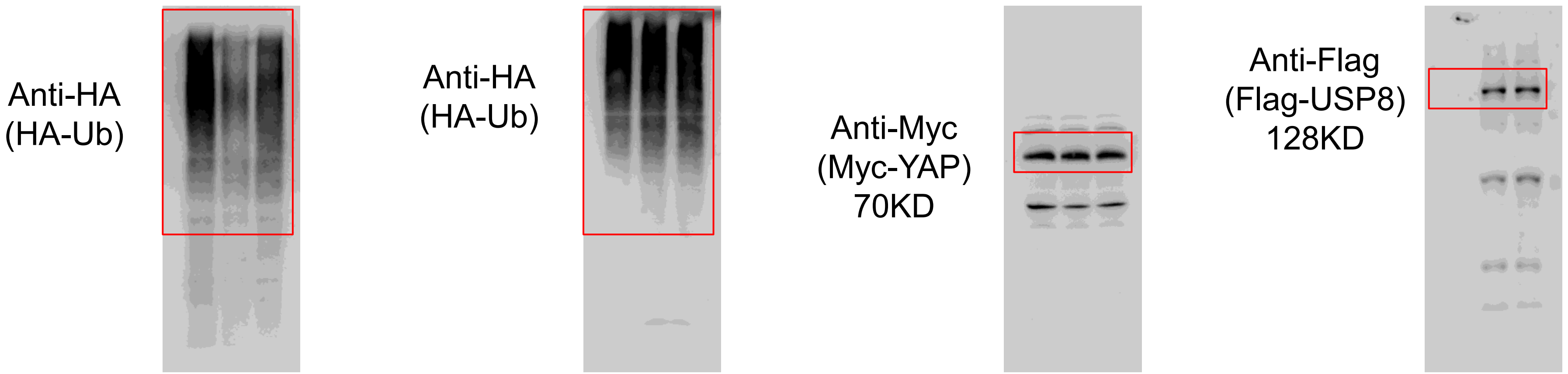
Anti-HA
(HA-Ub)
Anti-HA
(HA-Ub)
Anti-Myc
(Myc-YAP)
70KD
Anti-Flag
(Flag-USP8)
128KD

Figure 7J

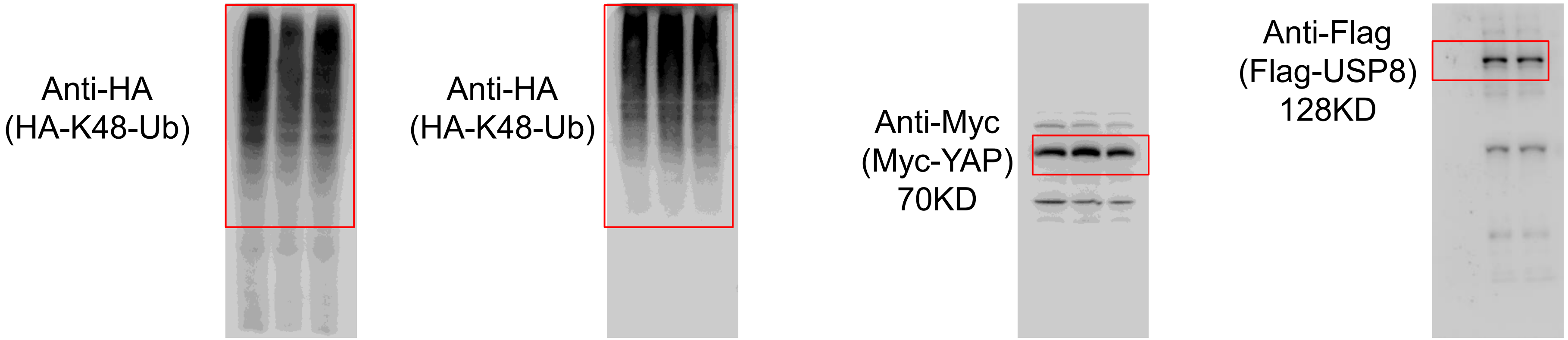
Anti-HA
(HA-K48-Ub)
Anti-HA
(HA-K48-Ub)
Anti-Myc
(Myc-YAP)
70KD
Anti-Flag
(Flag-USP8)
128KD

Figure 7K

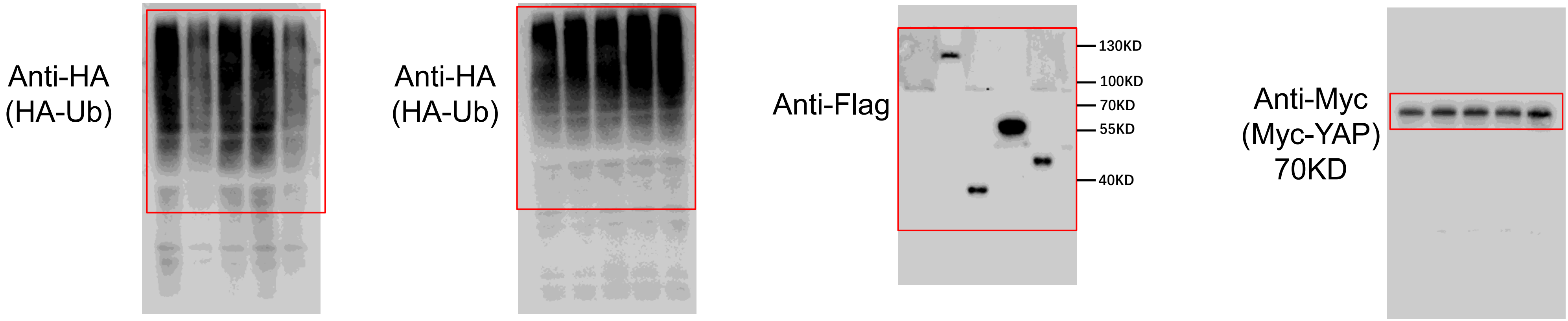
Anti-HA
(HA-Ub)
Anti-HA
(HA-Ub)
Anti-Flag
130KD
100KD
70KD
55KD
40KD
Anti-Myc
(Myc-YAP)
70KD

Figure 7L

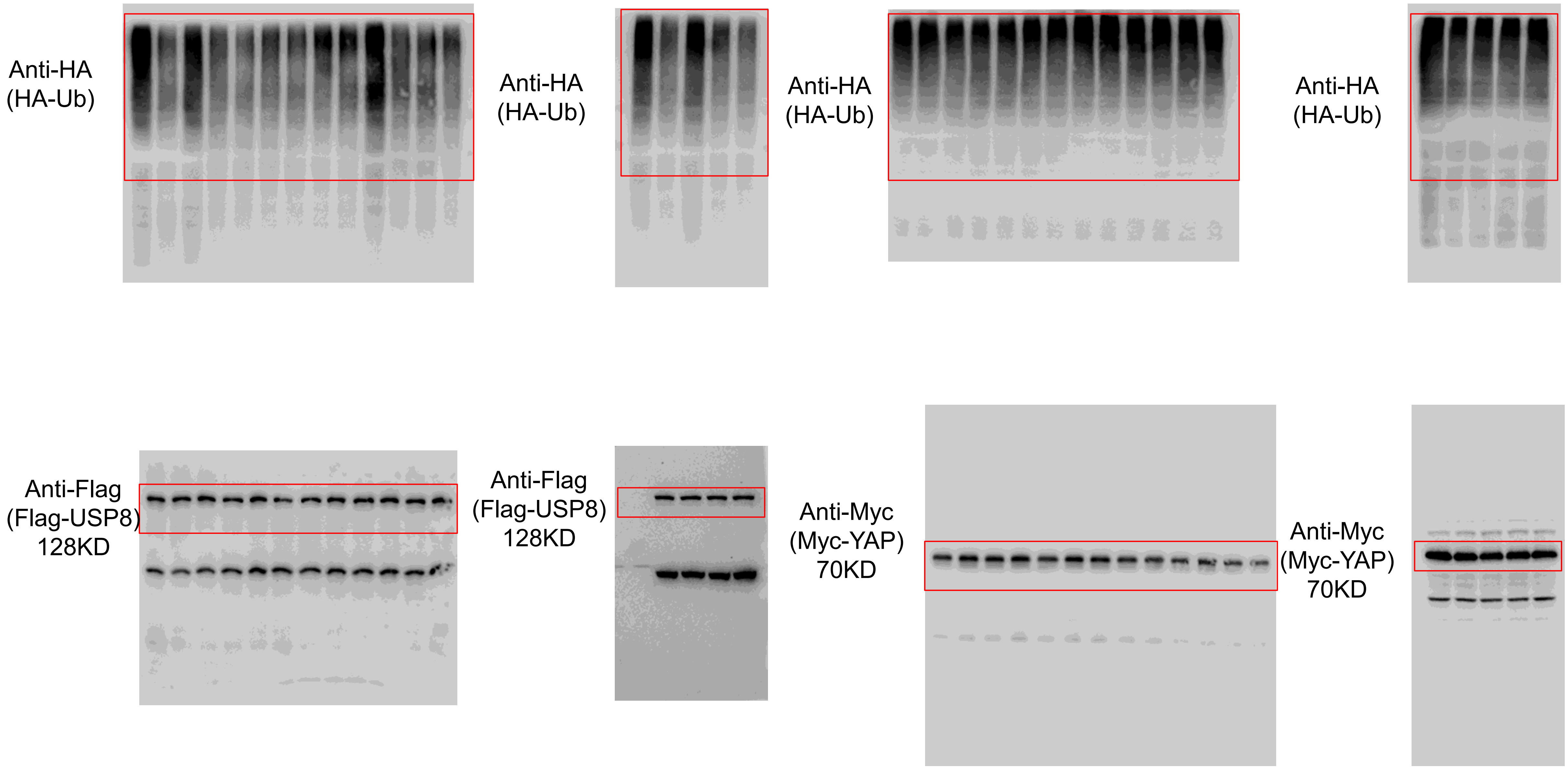
Anti-HA
(HA-Ub)
Anti-HA
(HA-Ub)
Anti-HA
(HA-Ub)
Anti-HA
(HA-Ub)
Anti-Flag
(Flag-USP8)
128KD
Anti-Flag
(Flag-USP8)
128KD
Anti-Myc
(Myc-YAP)
70KD
Anti-Myc
(Myc-YAP)
70KD

Figure 8H

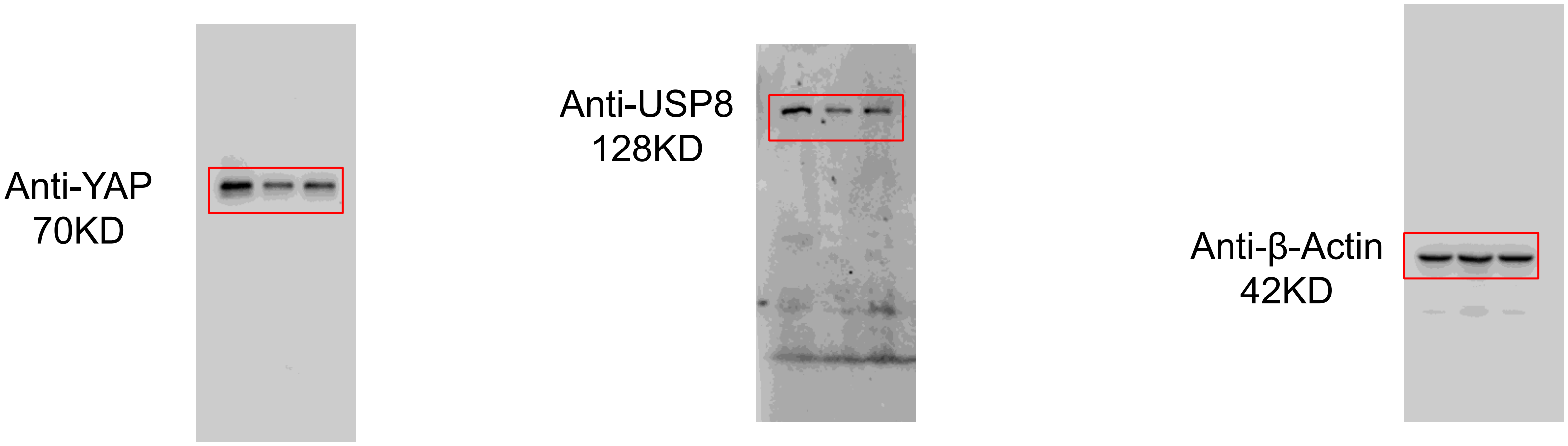

Figure 8I

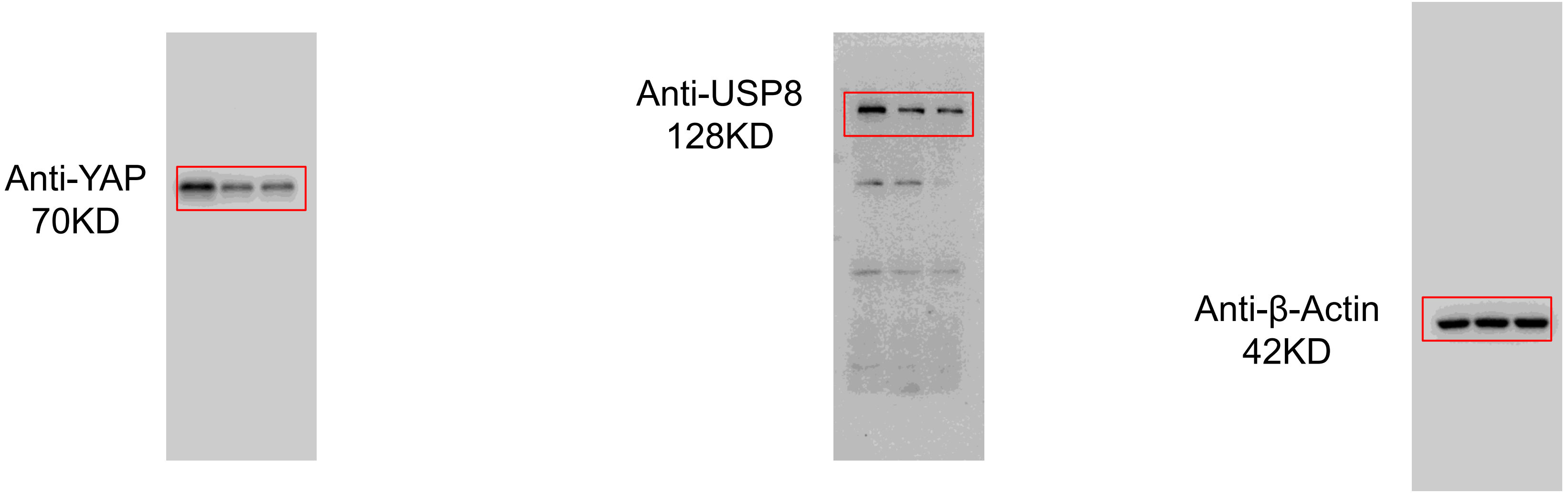

Figure 8P

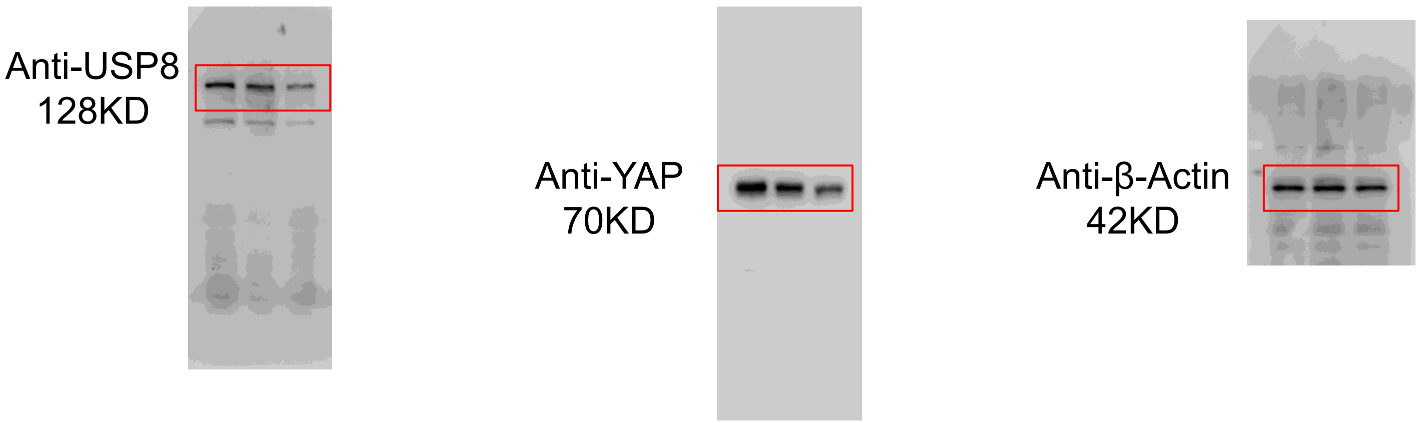

Figure 8Q

Anti-USP8
128KD

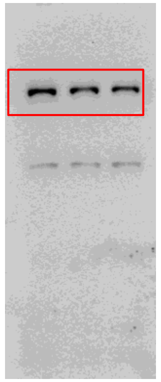

Anti-YAP
70KD

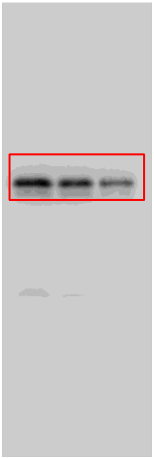

Anti-β-Actin
42KD

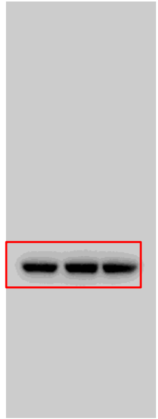

Supplementary Figure 3A

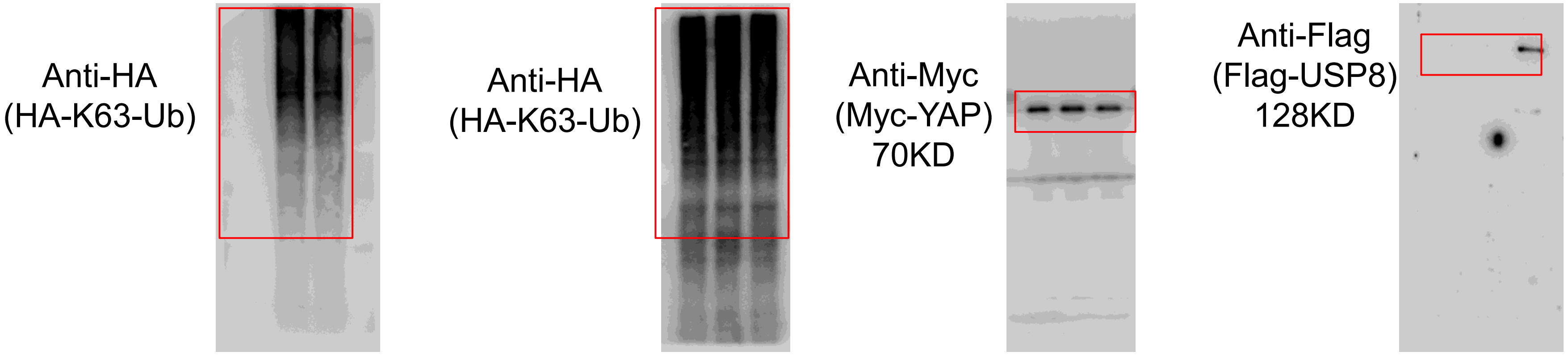

Supplementary Figure 3B

Anti-HA
(HA-K63R-Ub)

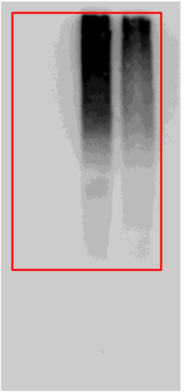

Anti-HA
(HA-K63R-Ub)

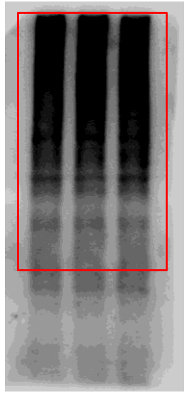

Anti-Myc
(Myc-YAP)
70KD

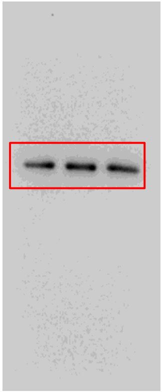

Anti-Flag
(Flag-USP8)
128KD

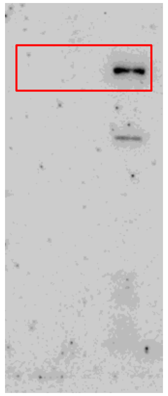

Supplementary Figure 3C

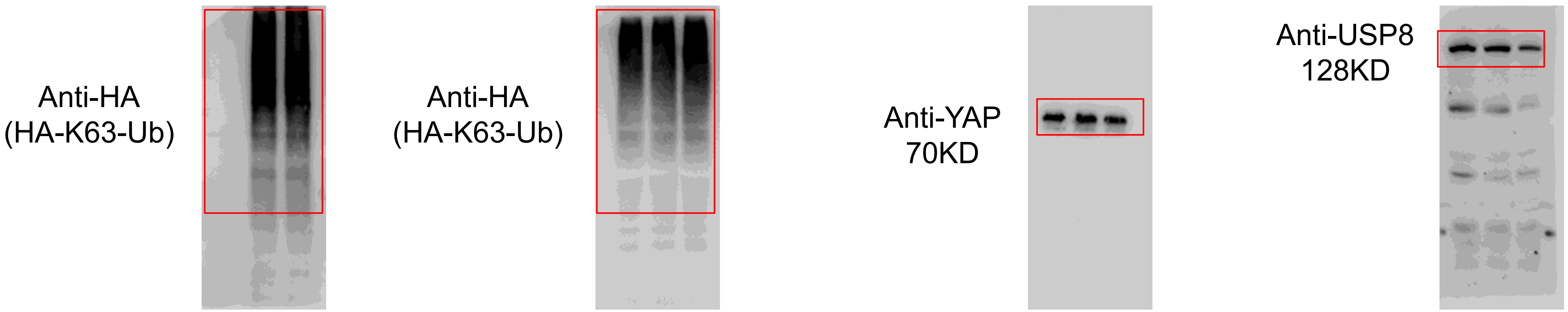

Supplementary Figure 3D

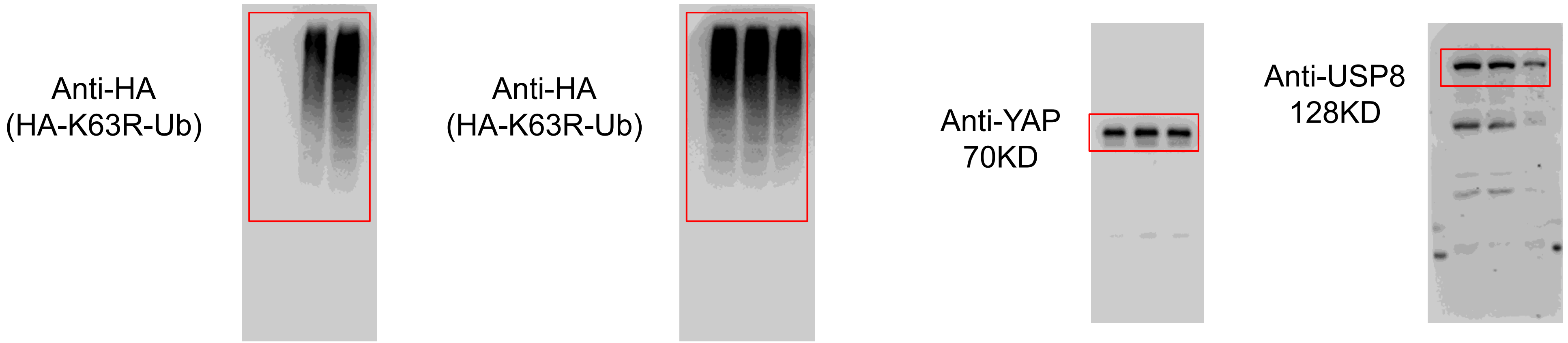

Supplementary Figure 3E

Anti-HA
(HA-K48R-Ub)

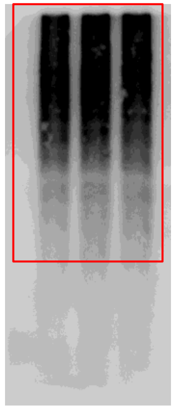

Anti-HA
(HA-K48R-Ub)

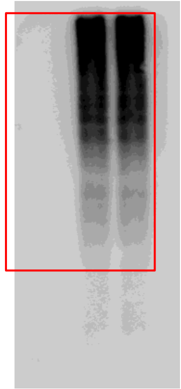

Anti-YAP
70KD

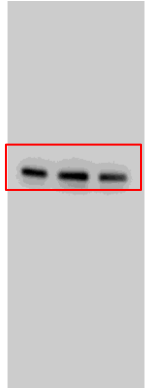

Anti-USP8
128KD

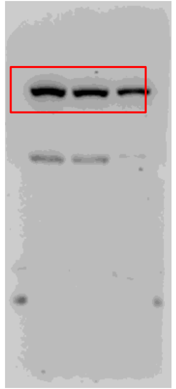

Supplementary Figure 3F

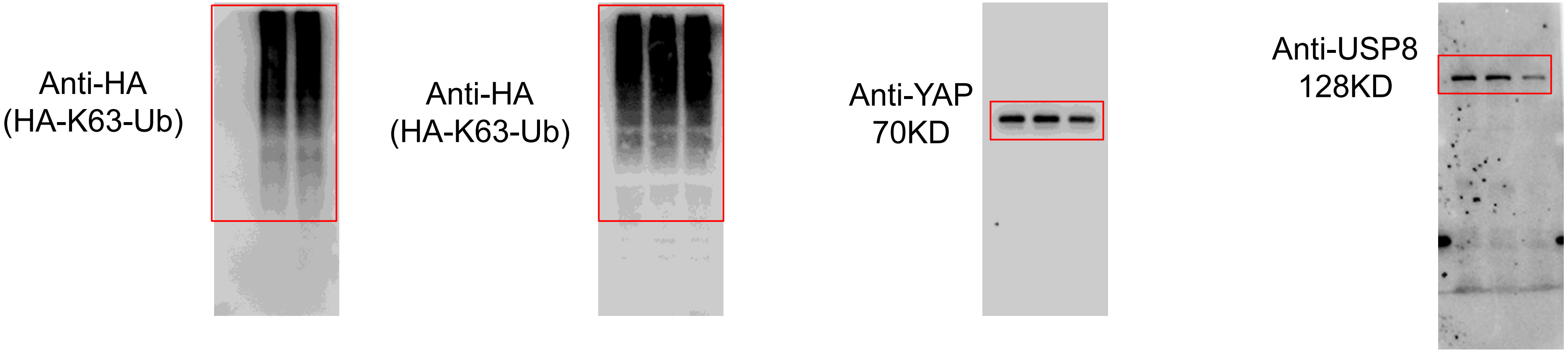

Supplementary Figure 3G

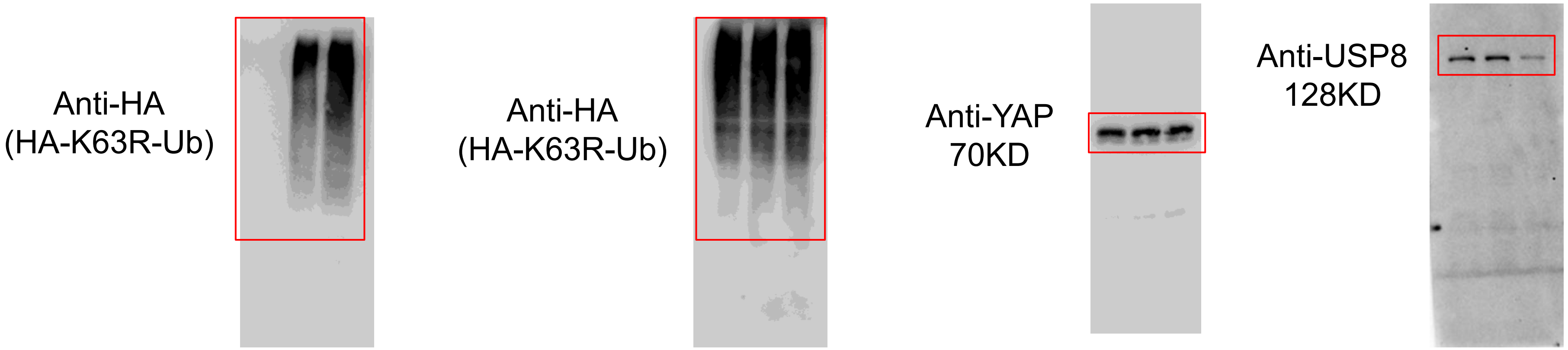

Supplementary Figure 3H

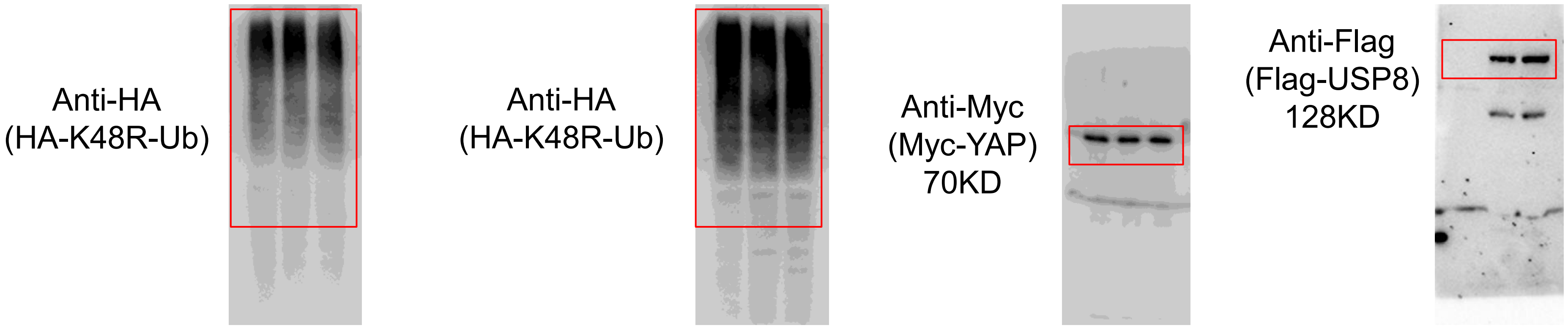

Supplementary Figure 3I

Anti-HA
(HA-K63-Ub)

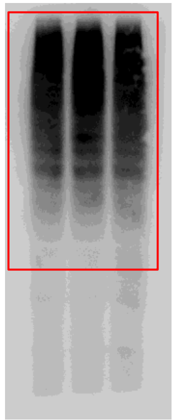

Anti-HA
(HA-K63-Ub)

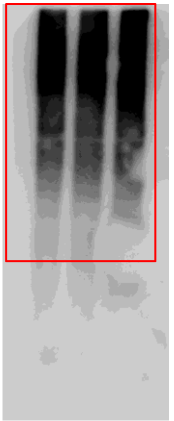

Anti-Myc
(Myc-YAP)
70KD

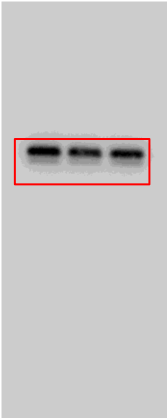

Anti-Flag
(Flag-USP8)
128KD

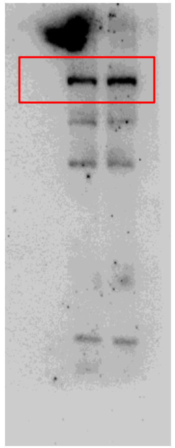

Supplementary Figure3J

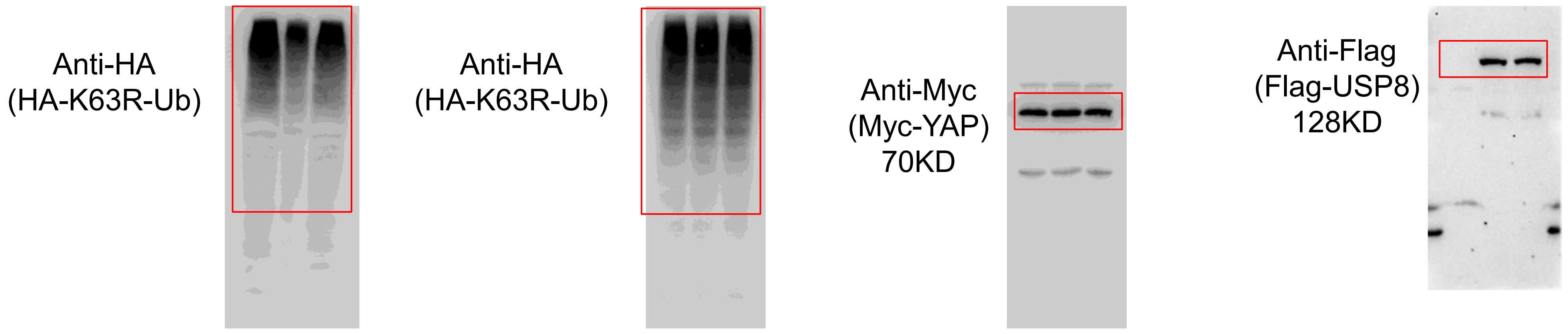

Supplementary Figure 3K

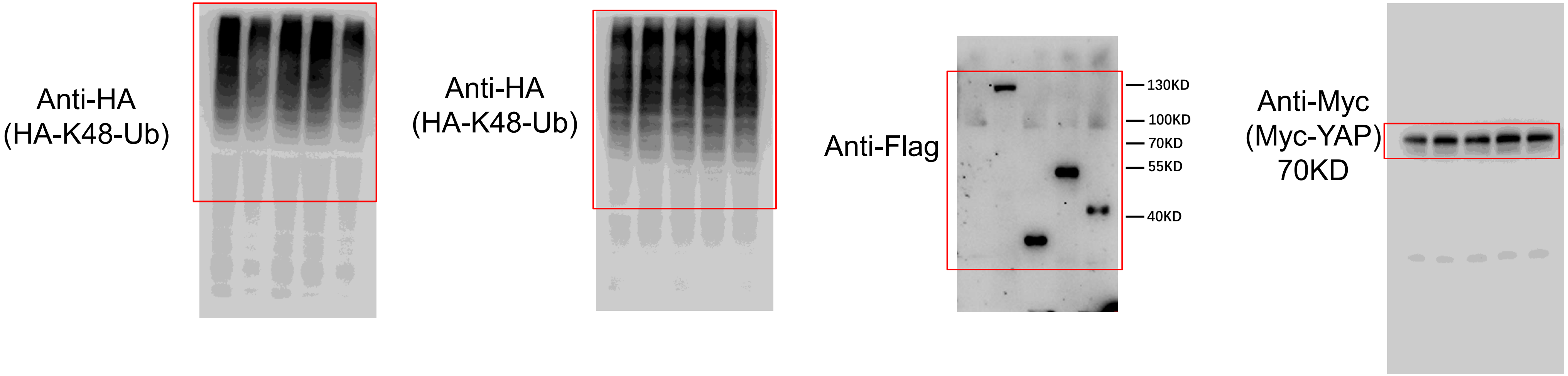

Supplementary Figure 3L

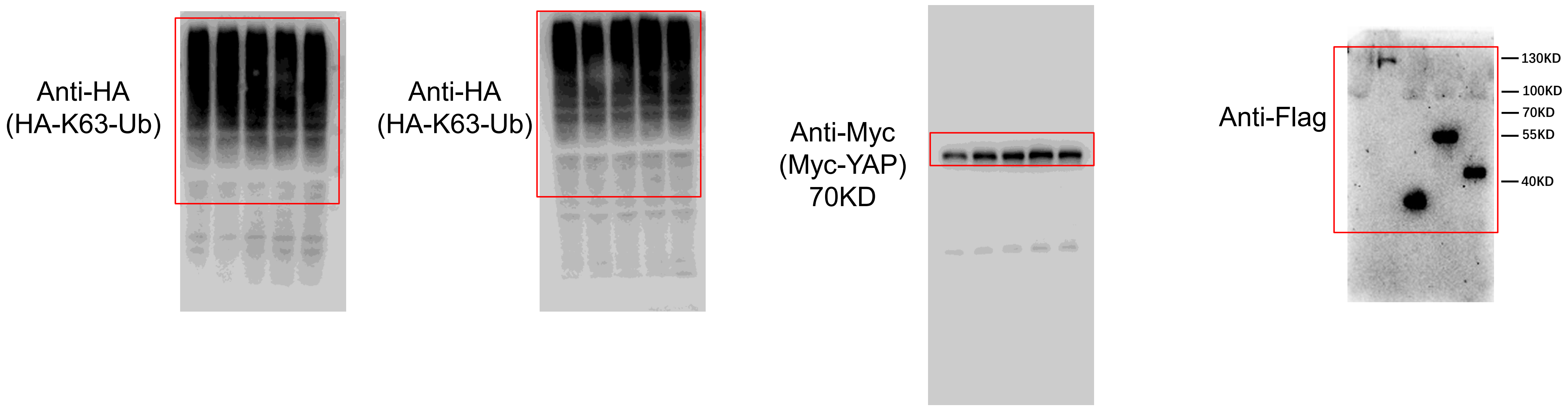

Supplement: Supplementary file 5 — Original Western Blot [file 41419_2025_8356_MOESM5_ESM.pdf]
