# Supplementary material for: Positive feedback regulation between USP8 and Hippo/YAP axis drives triple-negative breast cancer progression
Source: Cell Death Dis. 2026 Jan 21;17(1):98. doi: 10.1038/s41419-025-08356-8 (PMC12830590; doi:10.1038/s41419-025-08356-8)
Supplement: Supplementary file 6 — Original Data [file 41419_2025_8356_MOESM6_ESM.pdf]

Figure 2B

siControl			siUSP8#1			siUSP8#2		
1.088	0.973	0.939	0.052	0.093	0.105	0.120	0.158	0.103

siControl			siUSP8#1			siUSP8#2		
0.915	0.956	1.129	0.061	0.109	0.131	0.121	0.112	0.157

Figure 2C

Time(Days)	siControl			siUSP8#1			siUSP8#2		
0	1.1412	0.8809	0.9779	0.9745	0.9464	1.0790	1.1049	0.9232	0.9719
1	3.1881	2.5747	3.1623	2.6495	2.8664	2.8726	3.1320	3.2163	3.2621
2	5.3534	5.2598	4.9485	3.5918	3.2205	3.9905	4.4266	3.8044	3.4985
3	9.1590	9.1671	9.8836	4.5268	4.4394	5.4987	7.7286	6.7251	7.2867

Time(Days)	siControl			siUSP8#1			siUSP8#2		
0	1.0218	0.9935	0.9848	1.0222	0.9739	1.0038	1.1486	0.9491	0.9023
1	2.2489	2.3817	2.9122	1.7692	1.9348	1.7784	2.0345	2.0369	1.9557
2	4.7083	4.5389	4.9348	3.1664	3.4540	3.2791	3.3358	3.3678	3.2397
3	8.8491	8.6792	8.1176	6.4908	5.7232	6.0729	7.0213	7.7002	7.0952

Figure 2D

Time(h)	siControl			siUSP8#1			siUSP8#2		
0	0	0	0	0	0	0	0	0	0
24	0.500	0.520	0.550	0.250	0.230	0.300	0.375	0.380	0.350
48	0.875	0.900	0.950	0.375	0.350	0.420	0.625	0.600	0.650

Figure 2E

Time(h)	siControl			siUSP8#1			siUSP8#2		
0	0	0	0	0	0	0	0	0	0
24	0.419	0.372	0.436	0.284	0.256	0.255	0.316	0.335	0.370
48	0.970	0.843	0.913	0.398	0.456	0.452	0.563	0.630	0.515

Figure 2F

Migration

siControl			siUSP8#1			siUSP8#2		
0.987	0.969	1.043	0.310	0.289	0.324	0.388	0.415	0.431

Invasion

siControl			siUSP8#1			siUSP8#2		
1.030	0.974	0.996	0.310	0.276	0.282	0.487	0.446	0.432

Figure 2G

Migration

siControl			siUSP8#1			siUSP8#2		
0.961	0.984	1.055	0.385	0.358	0.328	0.458	0.430	0.471

Invasion

siControl			siUSP8#1			siUSP8#2		
0.997	1.025	0.978	0.337	0.309	0.286	0.490	0.466	0.490

Figure 2H

siControl	siUSP#1	siUSP#2
51.47	28.14	30.56
55.55	27.16	34.16
58.38	29.07	32.88

Figure 2I

siControl	siUSP8#1	siUSP8#2
34.14	17.3	28.79
35	18.7	27.5
34.2	18.5	26.5

Figure 2J

siControl			siUSP8#1			siUSP8#2		
7.5	8.13	7.3	13.21	12.15	11.17	11.01	10.74	9.48

Figure 2K

**siControl**			**siUSP8#1**			**siUSP8#2**		
4.89	4.98	4.36	8.27	8.36	8.51	8.16	8.2	8

Figure 2M

shControl					shUSP8				
0.7477	0.5633	0.6856	0.7571	0.5702	0.225	0.1674	0.2175	0.1391	0.2067

Figure 2N

Time(Days)	shControl					shUSP8				
0	0	0	0	0	0	0	0	0	0	0
7	0	0	0	0	0	0	0	0	0	0
14	120.753	77.55	99.805	111.975	62.049	50.999	31.9	38.597	16.576	25.959
21	204.274	241.824	282.517	250.825	251.593	127.309	64.587	92.984	48.146	93.412
28	439.497	482.472	414.362	490.377	497.369	151.301	91.682	168.131	120.99	149.64
35	820.378	698.714	737.065	946.85	818.727	263.757	149.335	248.261	188.225	263.103

Figure 2Q

shControl					shUSP8				
71.42	63.24	70.11	68.17	80.01	17.85	11.96	14.57	22.36	27.05

Figure 3B

DMSO			DUB-IN-2 (2 μM)			DUB-IN-2 (4 μM)		
1.036	1.137	0.827	0.450	0.418	0.469	0.337	0.364	0.326

DMSO			DUBs-IN-2 (2 μM)			DUBs-IN-2 (4 μM)		
1.007	1.019	0.974	0.452	0.491	0.559	0.197	0.223	0.196

Figure 3C

Time (Days)	DMSO			DUBs-IN-2 (2 μM)			DUBs-IN-2 (4 μM)		
0	1.0591	0.9693	0.9717	1.0211	1.0096	0.9693	1.1614	0.9351	0.9035
1	2.3228	2.4787	2.7669	2.2854	2.5010	2.3630	1.5799	1.5385	1.5093
2	4.1142	4.7047	4.3693	3.8812	4.1600	3.9157	2.5904	2.1632	2.6415
3	9.7032	9.6890	8.9992	6.7203	7.0881	7.3841	4.5929	3.7590	3.7447

Time (Days)	DMSO			DUBs-IN-2 (2 μM)			DUBs-IN-2 (4 μM)		
0	1.0136	0.9656	1.0208	0.9686	0.9455	1.0860	0.9133	1.0408	1.0460
1	4.2483	4.1616	4.7847	2.2942	1.9253	2.7522	1.3032	1.1542	1.9669
2	8.1612	8.6168	8.8554	5.2679	5.9352	5.5588	3.2578	3.9818	3.9911
3	10.1381	10.9901	10.6709	7.4265	7.1572	7.9238	5.9444	5.8948	5.1154

Figure 3F

Migration

DMSO			DUBs-IN-2 (2 μM)			DUBs-IN-2 (4 μM)		
1.040	0.976	0.984	0.701	0.662	0.684	0.434	0.429	0.401

Invasion

DMSO			DUBs-IN-2 (2 μM)			DUBs-IN-2 (4 μM)		
1.054	0.944	1.002	0.564	0.529	0.556	0.215	0.238	0.260

Figure 3G

Migration

DMSO			DUBs-IN-2 (2 μM)			DUBs-IN-2 (4 μM)		
1.035	0.970	0.996	0.487	0.461	0.409	0.266	0.243	0.230

Invasion

DMSO			DUBs-IN-2 (2 μM)			DUBs-IN-2 (4 μM)		
0.965	1.007	1.028	0.411	0.420	0.438	0.227	0.237	0.247

Figure 3H

DMSO	**DUB-IN-2 (2 µM)**	**DUB-IN-2 (4 µM)**
51.97	36.96	29.07
54.77	37.38	27.9
52.26	42.73	27.5

Figure 3I

**DMSO**	**DUB-IN-2 (2 µM)**	**DUB-IN-2 (4 µM)**
30.47	26.53	18.22
31.29	25.36	17.29
33.14	22.7	16.18

Figure 3D

Time(h)	DMSO			DUBs-IN-2(2 μM)			DUBs-IN-2(4 μM)		
0	0	0	0	0	0	0	0	0	0
24	0.300	0.350	0.330	0.250	0.230	0.270	0.210	0.230	0.235
48	0.800	0.850	0.810	0.430	0.460	0.480	0.330	0.350	0.390

Figure 3E

Time(h)	DMSO			**DUB-IN-2(2 μM)**			**DUB-IN-2(4 μM)**		
0	0	0	0	0	0	0	0	0	0
24	0.604	0.650	0.623	0.265	0.306	0.359	0.091	0.147	0.110
48	0.959	0.926	0.911	0.441	0.486	0.487	0.303	0.338	0.347

Figure 3J

DMSO			DUB-IN-2(2 μM)			DUB-IN-2(4 μM)		
16.95	15.76	17.82	25.71	22.75	23.67	38	33.22	35.63

Figure 3K

DMSO			DUB-IN-2 (2 μM)			DUB-IN-2 (4 μM)		
4.81	4.5	4.67	6.39	6.55	6.75	8.36	8.53	8.61

Figure 3M

DMSO					DUB-IN-2				
0.7102	0.8349	0.5764	0.7230	0.5332	0.1707	0.2945	0.1558	0.2819	0.1840

Figure 3N

Time(Days)	DMSO					DUB-IN-2				
0	0	0	0	0	0	0	0	0	0	0
7	0	0	0	0	0	0	0	0	0	0
14	68.447	123.191	82.681	82.454	87.373	28.945	88.519	35.833	60.171	28.976
21	207.512	276.488	228.500	223.723	223.538	75.563	110.198	70.378	119.098	92.571
28	404.020	458.635	403.575	404.083	400.105	109.445	210.323	103.312	260.171	169.677
35	802.419	853.523	826.745	818.414	626.899	251.142	370.384	251.297	328.591	256.423

Figure 3O

DMSO					DUB-IN-2				
56.01	58.75	78.85	76.40	73.82	27.58	24.64	23.37	19.94	25.75

Figure 4C

siControl			siUSP8#1			siUSP8#2		
0.938	1.015	1.047	0.956	1.137	1.021	1.032	1.033	0.903

Figure 4D

siControl			siUSP8#1			siUSP8#2		
1.144	0.874	0.982	1.009	0.865	0.986	0.859	0.945	0.890

Figure 4E

	siControl			siUSP8#1			siUSP8#2		
CTGF	0.978	1.014	1.008	0.415	0.417	0.379	0.382	0.464	0.423
CYR61	1.007	1.039	0.954	0.325	0.368	0.340	0.523	0.359	0.456

Figure 4F

	siControl			siUSP8#1			siUSP8#2		
CTGF	0.919	1.033	1.047	0.414	0.497	0.397	0.694	0.631	0.627
CYR61	0.985	1.118	0.897	0.482	0.411	0.470	0.600	0.545	0.566

Figure 4G

siControl			siUSP8#1			siUSP8#2		
1.133	0.995	0.871	0.473	0.453	0.552	0.494	0.616	0.578

Figure 4H

siControl			siUSP8#1			siUSP8#2		
1.080	1.031	0.890	0.457	0.439	0.506	0.473	0.425	0.462

Figure 4K

DMSO			DUBs-IN-2 (2 μM)			DUBs-IN-2 (4 μM)		
0.932	1.135	0.934	0.805	0.902	0.806	0.881	0.935	0.911

Figure 4L

DMSO			DUBs-IN-2 (2 μM)			DUBs-IN-2 (4 μM)		
1.113	0.980	0.907	1.125	1.184	1.196	1.198	1.110	0.939

Figure 4M

	DMSO			DUBs-IN-2 (2 μM)			DUBs-IN-2 (4 μM)		
CTGF	0.894	1.072	1.034	0.211	0.223	0.192	0.165	0.163	0.181
CYR61	0.956	1.102	0.943	0.311	0.342	0.288	0.233	0.270	0.205

Figure 4N

	DMSO			DUBs-IN-2 (2 μM)			DUBs-IN-2 (4 μM)		
CTGF	0.956	1.079	0.965	0.593	0.620	0.675	0.334	0.310	0.245
CYR61	1.087	0.878	1.034	0.543	0.601	0.638	0.326	0.333	0.277

Figure 4O

DMSO			DUBs-IN-2 (2 μM)			DUBs-IN-2 (4 μM)		
1.087	0.975	0.938	0.608	0.631	0.640	0.408	0.416	0.351

Figure 4P

DMSO			DUBs-IN-2 (2 μM)			DUBs-IN-2 (4 μM)		
1.120	0.992	0.888	0.576	0.603	0.594	0.408	0.437	0.386

Figure 5B

	siControl			siUSP8			siUSP8+Myc-YAP		
CTGF	0.985	1.118	0.897	0.373	0.468	0.513	0.845	0.756	0.733
CYR61	0.919	1.033	1.047	0.338	0.460	0.400	0.804	0.625	0.667

Figure 5C

siControl			siUSP8			siUSP8+Myc-YAP		
1.134	0.931	0.935	0.473	0.426	0.454	0.762	0.857	0.798

Figure 5D

Time(Days)	siControl			siUSP8			siUSP8+Myc-YAP		
0	0.9791	1.0678	0.9531	1.0343	0.9520	1.0137	1.0111	1.0007	0.9882
1	3.7289	3.9257	4.2286	2.3498	2.1914	2.6745	3.4247	3.6185	3.0457
2	7.6128	6.9402	7.1456	3.6578	4.3162	3.3512	5.7495	5.7224	5.1767
3	8.5573	9.4441	8.3107	5.4081	5.6612	6.1859	7.3539	7.6163	7.1041

Figure 5E

siControl	siUSP8	siUSP8+Myc-YAP
55.56	21.14	46.83
51.47	15.56	45.03
54.46	19.46	43.96

Figure 5F

Time(h)	siControl			siUSP8			siUSP8+Myc-YAP		
0	0	0	0	0	0	0	0	0	0
24	0.500	0.518	0.589	0.193	0.123	0.211	0.411	0.339	0.375
48	0.786	0.768	0.821	0.474	0.456	0.386	0.643	0.625	0.696

Figure 5G

Migration

siControl			siUSP8			siUSP8+Myc-YAP		
0.923	0.983	1.094	0.350	0.362	0.394	0.715	0.715	0.741

Invasion

siControl			siUSP8			siUSP8+Myc-YAP		
1.029	1.003	0.968	0.405	0.422	0.482	0.753	0.736	0.713

Figure 5I

shControl					shUSP8					shUSP8+YAP				
0.4532	0.4724	0.5489	0.4996	0.3823	0.1392	0.1148	0.1261	0.0948	0.1672	0.3505	0.3723	0.3828	0.3900	0.3860

Figure 5J

Time(Days)	shControl					shUSP8					shUSP8+YAP				
0	0	0	0	0	0	0	0	0	0	0	0	0	0	0	0
7	0	0	0	0	0	0	0	0	0	0	0	0	0	0	0
14	75.850	83.463	87.032	119.660	81.268	69.184	18.894	23.481	16.516	20.072	65.523	68.174	69.485	65.282	67.774
21	245.263	209.013	207.744	236.622	218.112	109.691	35.729	56.031	47.141	70.412	125.639	127.587	164.237	126.624	122.820
28	421.387	471.618	414.383	410.391	409.521	141.010	65.404	103.633	96.440	97.338	215.793	228.745	255.161	210.265	216.746
35	756.618	795.108	653.396	667.144	686.071	205.441	109.418	168.631	173.285	146.970	351.563	439.071	361.735	352.276	427.372

Figure 5L

**shControl**					**shUSP8**					**shUSP8+YAP**				
75.44	78.75	62.45	79.29	80.74	23.93	27.37	26.94	31.56	33.62	45.15	46.43	46.99	41.16	43.23

Figure 6K

CHX(Hours)	**siControl**			**siUSP8**		
0	1.006	1.008	0.986	0.996	1.000	1.005
3	0.813	0.806	0.764	0.544	0.563	0.537
6	0.492	0.545	0.555	0.273	0.319	0.265
9	0.307	0.263	0.318	0.157	0.148	0.152

Figure 6N

CHX(Hours)	**siControl**			**siUSP8**		
0	0.996	1.009	0.995	0.990	1.001	1.009
3	0.678	0.618	0.624	0.538	0.498	0.473
6	0.433	0.403	0.459	0.250	0.190	0.204
9	0.139	0.120	0.170	0.065	0.068	0.045

Figure 6P

CHX(Hours)	**Flag**			**Flag-USP8**			**Flag-USP8^{C786A}**		
0	1.010	0.980	1.009	0.986	1.005	1.009	1.000	0.991	1.009
3	0.492	0.535	0.456	0.790	0.822	0.766	0.697	0.700	0.694
6	0.214	0.180	0.165	0.395	0.457	0.396	0.194	0.211	0.236
9	0.042	0.063	0.098	0.217	0.253	0.197	0.059	0.079	0.099

Figure 8B

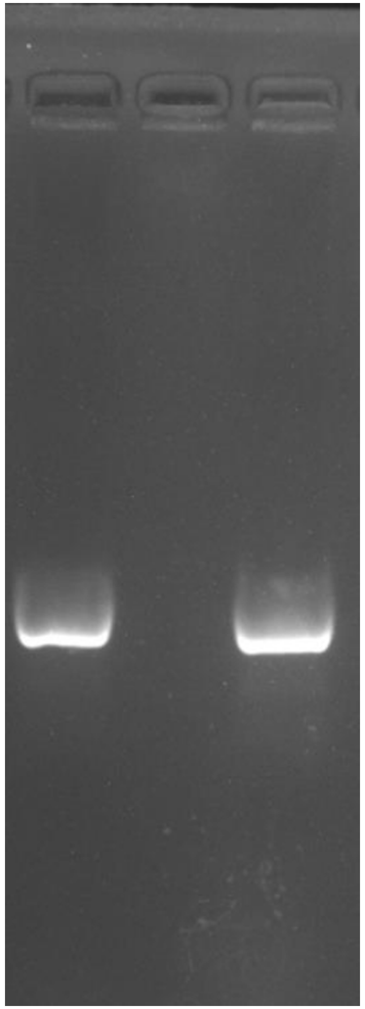

Figure 8E

	IgG			anti-YAP		
site1	0.982	1.064	0.955	27.307	26.357	28.973
site2	0.878	0.927	1.194	1.247	0.901	0.851
site3	1.147	0.912	0.941	0.944	1.168	0.888

Figure 8G

	**WT**			**siYAP#1**			**siYAP#2**		
WT-promoter	0.983	1.034	0.983	0.373	0.491	0.389	0.481	0.463	0.390
Mut-promoter	1.034	0.956	1.010	0.959	0.953	0.891	0.969	0.925	0.936

Figure 8J

siControl			siYAP#1			siYAP#2		
1.104	0.982	0.913	0.359	0.383	0.343	0.404	0.472	0.433

Figure 8K

siControl			siYAP#1			siYAP#2		
1.039	1.052	0.909	0.403	0.367	0.365	0.435	0.381	0.421

Figure 8L

	siControl			siYAP#1			siYAP#2		
**CTGF**	1.027	0.985	0.988	0.464	0.494	0.464	0.540	0.582	0.555
**USP8**	1.017	0.969	1.015	0.480	0.423	0.479	0.466	0.519	0.511

Figure 8M

	siControl			siYAP#1			siYAP#2		
CTGF	1.079	1.018	0.903	0.541	0.519	0.561	0.536	0.572	0.570
USP8	0.965	1.125	0.909	0.402	0.453	0.409	0.397	0.517	0.491

Figure 8N

	siControl			siYAP		
IgG	0.922	0.939	1.139	0.995	0.954	1.050
Anti-YAP	15.642	14.532	14.449	4.265	5.200	4.234

Figure 8O

	siControl			siYAP		
IgG	0.911	1.168	0.921	0.893	0.975	1.132
Anti-YAP	17.676	17.373	18.690	5.884	6.559	6.695

Figure 8R

**DMSO**			**Verteporfin(3 μM)**			**Verteporfin(6 μM)**		
1.126	0.939	0.935	0.474	0.426	0.502	0.230	0.218	0.277

Figure 8S

**Verteporfin(0 μM)**			**Verteporfin(3 μM)**			**Verteporfin(6 μM)**		
0.991	0.914	1.095	0.479	0.640	0.525	0.517	0.385	0.355

Figure 8T

	**Verteporfin(0 μM)**			**Verteporfin(3 μM)**			**Verteporfin(6 μM)**		
**CTGF**	1.070	0.979	0.952	0.355	0.392	0.370	0.171	0.145	0.146
**USP8**	1.061	0.932	1.007	0.240	0.263	0.397	0.092	0.124	0.113

Figure 8U

	**Verteporfin(0 μM)**			**Verteporfin(3 μM)**			**Verteporfin(6 μM)**		
**CTGF**	1.076	0.833	1.092	0.307	0.335	0.375	0.144	0.170	0.154
**USP8**	0.992	0.936	1.072	0.448	0.494	0.448	0.271	0.288	0.257

Figure 8V

	DMSO			Verteporfin(3 μM)		
IgG	0.931	0.921	1.148	0.956	0.975	1.069
Anti-YAP	16.337	16.205	14.457	8.318	8.036	7.919

Figure 8W

	DMSO			Verteporfin(3 μM)		
IgG	0.932	0.967	1.101	0.956	0.973	1.071
Anti-YAP	13.642	14.333	14.808	7.530	6.952	6.463

Figure 9A

	PTX			PTX+DUB-IN-2		
0	100	100	100	100	100	100
5	92.82	97.96	98.31	98.84	98.41	98.32
10	90.59	96.66	95.37	80.75	86.56	85.15
15	83.21	90.42	88.11	50.6	58.21	52.44
20	69.97	75.59	70.53	31.43	39.24	33.91
25	38.68	45.92	40.43	20.61	27.57	23.16
30	24.32	30.54	25.94	11.74	18.43	14.19
35	9.27	15.26	10.56	6.28	12.46	9.29
40	4.45	9.48	4.06	4.23	7.26	5.15

Figure 9B

	PTX			PTX+DUB-IN-2		
0	100	100	100	100	100	100
5	93.00	97.29	99.67	99.04	98.79	99.70
10	92.08	96.55	98.31	91.81	96.49	93.83
15	90.89	93.07	97.64	86.48	89.18	81.43
20	88.54	83.56	90.43	78.56	74.66	71.74
25	80.31	82.17	75.20	41.73	45.70	38.53
30	55.20	58.67	62.35	27.13	31.31	24.86
35	40.26	43.72	35.71	16.79	19.99	12.44
40	26.61	28.93	21.41	13.56	9.62	7.13

Figure 9C

Time(Days)	DMSO			**PTX**			**PTX+DUB-IN-2**		
0	0.9145	1.0777	1.0078	0.9549	0.9724	1.0726	1.0549	1.0727	0.8724
1	2.0435	2.2135	2.5478	1.8294	1.8078	1.9467	1.2879	1.2449	1.2986
2	4.8826	4.5077	4.2715	3.7358	3.4777	3.2130	2.0136	1.9495	1.9937
3	9.5161	9.0590	9.8414	5.9171	6.6375	6.3658	2.7070	2.5703	2.4114

Figure 9D

Time(Days)	**DMSO**			**PTX**			**PTX+DUB-IN-2**		
0	0.9791	1.0678	0.9531	1.0343	0.9520	1.0137	1.0111	1.0007	0.9882
1	1.7823	1.7628	1.8493	1.5267	1.3683	1.3457	1.3428	1.3366	1.3720
2	5.2336	4.5609	4.7664	3.8635	3.9355	3.9685	3.6676	3.6405	3.4948
3	9.2062	9.4441	9.9922	5.8196	6.0727	6.3916	4.4393	4.6017	4.0895

Figure 9E

Time(h)	DMSO			**PTX**			**PTX+DUB-IN-2**		
0	0	0	0	0	0	0	0	0	0
24	0.3750	0.3333	0.4571	0.2333	0.2500	0.3088	0.1515	0.1563	0.1112
48	0.9063	0.9142	0.9529	0.5967	0.5375	0.5441	0.2933	0.3138	0.2765

Figure 9F

Time(h)	**DMSO**			**PTX**			**PTX+DUB-IN-2**		
0	0	0	0	0	0	0	0	0	0
24	0.3968	0.4230	0.3870	0.2500	0.2300	0.2217	0.0952	0.1570	0.1240
48	0.9206	0.9349	0.8413	0.3750	0.4688	0.4063	0.2305	0.2522	0.2210

Figure 9G

Migration

DMSO			**PTX**			**PTX+DUB-IN-2**		
0.984	1.026	0.990	0.698	0.664	0.625	0.161	0.219	0.187

Invasion

**DMSO**			**PTX**			**PTX+DUB-IN-2**		
0.966	1.029	1.006	0.617	0.567	0.611	0.088	0.128	0.131

Figure 9H

Migration

DMSO			PTX			PTX+DUB-IN-2		
1.032	0.975	0.993	0.584	0.619	0.601	0.143	0.122	0.158

Invasion

DMSO			PTX			PTX+DUB-IN-2		
1.017	0.989	0.995	0.457	0.479	0.401	0.142	0.176	0.153

Figure 9I

DMSO	PTX	PTX+DUB-IN-2
42.15	30.69	21.76
46.83	34.96	19.46
48.03	31.85	22.14

Figure 9J

DMSO	PTX	PTX+DUB-IN-2
36.33	29.51	11.55
34.21	27.61	15.43
37.49	25.43	14.71

Figure 9L

DMSO					PTX					**PTX+DUB-IN-2**				
0.6609	0.4925	0.6092	0.4931	0.6676	0.3112	0.2672	0.1464	0.1902	0.1236	0.0466	0.0691	0.0806	0.0686	0.0503

Figure 9M

Time(Days)	DMSO					PTX					**PTX+DUB-IN-2**				
0	0	0	0	0	0	0	0	0	0	0	0	0	0	0	0
7	0	0	0	0	0	0	0	0	0	0	0	0	0	0	0
14	92.485	173.232	131.487	122.707	132.410	115.969	87.438	66.422	68.992	40.864	15.167	16.769	22.934	13.150	12.225
21	271.912	241.100	220.586	209.114	281.270	184.667	135.796	106.314	143.560	99.424	25.586	26.145	37.296	28.691	26.394
28	507.278	517.166	570.942	502.105	504.406	242.014	287.031	199.215	215.339	162.367	34.741	37.760	47.181	48.936	41.891
35	1229.256	715.255	1004.102	864.697	834.319	355.227	317.765	272.168	303.823	200.815	51.255	45.057	64.322	76.250	53.237

Figure 9N

DMSO					**PTX**					**PTX+DUB-IN-2**				
51.380	54.020	58.820	54.720	49.530	32.620	26.478	31.449	35.063	29.662	11.620	14.090	12.136	13.088	15.801
