# Supplementary material for: Positive feedback regulation between USP8 and Hippo/YAP axis drives triple-negative breast cancer progression
Source: Cell Death Dis. 2026 Jan 21;17(1):98. doi: 10.1038/s41419-025-08356-8 (PMC12830590; doi:10.1038/s41419-025-08356-8)
Supplement: Supplementary file 7 — Supplementary Materials [file 41419_2025_8356_MOESM7_ESM.docx]

**Supplemental information**

**Supplementary Table 1.** Sequences of siUSP8, siYAP,shUSP8

| **Gene** | **Sequences** |
| --- | --- |
| siUSP8-1 | 5’-GGA CCA CUG AAU AUA UCA ATT-3’  5’-UUG AUA UAU UCA GUG GUC CTT-3’ |
| siUSP8-2 | 5’-GGG CCU AUG UAC UAU AUA UTT-3’  5’-AUA UAU AGU ACA UAG GCC CTT-3’ |
| siControl | 5’-UUC UCC GAA CGU GUC ACG UTT-3’  5’-ACG UGA CAC GUU CGG AGA ATT-3’ |
| siYAP-1 | 5’-GUC AGA GAU ACU UCU UAA ATT-3’  5’-UUU AAG AAG UAU CUC UGA CTT-3’ |
| siYAP-2 | 5’-GUC UCA GGA AUU GAG AAC ATT-3’  5’-UGU UCU CAA UUC CUG AGA CTT-3’ |
| shUSP8 | F:5’-CCGGCCACAGATTGATCGTACTAAACTCGAGTTTA  GTACGATCAATCTGTGGTTTTTG-3’  F:5’-AATTCAAAAACCACAGATTGATCGTACTAAACTCG  AGTTTAGTACGATCAATCTGTGG-3’ |

**Supplementary Table 2.** Primer sequences for qRT-PCR

| **Gene** | **Sequences** |
| --- | --- |
| 36B4 | F:5’-GCAGCATCTACAACCCTGAAG-3’  R:5’-CACTGGCAACATTGCGGAC-3’ |
| USP8 | F:5’-AAGGAGCAATCACAGCAAAGG-3’  R:5’-CTGCATTCTTCGAGCATCCATTA-3’ |
| YAP | F:5’-CAAGAAAGCAGGCTCACAGAA -3’  R:5’- GCTGGGTGTTAGGGCTTCG-3’ |
| CTGF | F:5’-ACCGACTGGAAGACACGTTTG-3’  R:5’- CCAGGTCAGCTTCGCAAGG-3’ |
| CYR61 | F:5’- GGTCAAAGTTACCGGGCAGT-3’  R:5’- GGAGGCATCGAATCCCAGC-3’ |

**Abbreviations:** F, forward; R, reverse.

**Supplementary Table 3.** Primer sequences for ChIP-qPCR

| **Gene** | **Sequences** |
| --- | --- |
| USP8 | F:5’-AGAGGAAAAGGGTGCAACAGC-3’  R:5’-AGAACCCAGGTTTCCTGATTCC-3’ |
| USP8-site1 | F:5’- AGCTGTCCTTATGTCTCTTACCA-3’  R:5’- TCCATAGAATCTAAAACTCCCCAA-3’ |
| USP8-site2 | F:5’-CCTCGCTAGCCAGATTTCCC-3’  R:5’TTGGGTGGAGCTTTAGGCAG-3’ |
| USP8-site3 | F:5’- GCCAGAAATAGAGGGGCGTT-3’  R:5’- CTGGGCCTTGTTGTGGAAGA-3’ |

**Abbreviations:** F, forward; R, reverse.

**Supplementary Table 4.** Antibodies used in the present study.

| **Target** | **Source** | **No. of Catalogue** |
| --- | --- | --- |
| Anti-β-Actin | Proteintech | 20536-1-AP |
| Anti-USP8 | Proteintech | 27791-1-AP |
| Anti-USP8 | Cell Signaling Technology | 8782S |
| Anti-YAP | Cell Signaling Technology | 14074 |
| Anti-YAP | Santa Cruz Biotechnology | sc-101199 |
| Anti-Flag | Sigma-Aldrich | F9291 |
| Anti-Flag | Cell Signaling Technology | 14793S |
| Anti-Myc | Proteintech | 16286-1-AP |
| Anti-HA | Cell Signaling Technology | 3724 |
| Anti-HA | Biolegend | 90513 |
| Rabbit IgG | Beyotime | A7016 |
| Mouse IgG | Beyotime | A7028 |
| HRP-labeled Goat  Anti-Rabbit IgG(H+L) | Beyotime | A0208 |
| HRP-labeled Goat  Anti-Mouse IgG(H+L) | Beyotime | A0216 |
| Goat Anti-Rabbit IgG | Abways | AB0141 |
| Goat Anti-Mouse IgG | Abways | AB0152 |
| Protein A+G Agarose | Beyotime | P2028 |

**Supplementary Table 5.** Results of molecular docking

Receptor Ligand Binding energy Interface area (Å^2)^ Hydrogen bonds

(YAP:USP8)

SER 257:GLN965

YAP USP8 -28.4 kcal/mol 5586.9

THR255:ARG969
